# Role of circulating T follicular helper subsets following Ty21a immunization and oral challenge with wild type *S*. Typhi in humans

**DOI:** 10.3389/fimmu.2024.1384642

**Published:** 2024-09-12

**Authors:** Jayaum S. Booth, Rekha R. Rapaka, Monica A. McArthur, Stephanie Fresnay, Thomas C. Darton, Christoph J. Blohmke, Claire Jones, Claire S. Waddington, Myron M. Levine, Andrew J. Pollard, Marcelo B. Sztein

**Affiliations:** ^1^ Center for Vaccine Development and Global Health, University of Maryland School of Medicine, Baltimore, MD, United States; ^2^ Department of Pediatrics, University of Maryland School of Medicine, Baltimore, MD, United States; ^3^ Department of Medicine, University of Maryland School of Medicine, Baltimore, MD, United States; ^4^ Global Clinical Development, Sanofi, Swiftwater, PA, United States; ^5^ Rockville Center for Vaccine Research, GlaxsoSmithKline (GSK), Rockville, MD, United States; ^6^ Oxford Vaccine Group, Department of Pediatrics, University of Oxford, and the National Institute for Health and Care Research (NIHR), Oxford Biomedical Research Centre, Oxford, United Kingdom; ^7^ Clinical Infection Research Group, Division of Clinical Medicine, School of Medicine and Population Health, University of Sheffield, and the National Institute for Health and Care Research (NIHR), Sheffield Biomedical Research Centre, Sheffield, United Kingdom; ^8^ GlaxsoSmithKline (GSK) Vaccines, London, United Kingdom; ^9^ Department of Infection, Imperial College Healthcare, National Health Service (NHS) Trust, London, United Kingdom; ^10^ Department of Medicine, Imperial College London, London, United Kingdom; ^11^ Tumor Immunology and Immunotherapy Program, University of Maryland Marlene and Stewart Greenebaum Comprehensive Cancer Center, Baltimore, MD, United States

**Keywords:** cTfh, circulating follicular helper T cells, typhoid fever, CHIM, *S.* Typhi

## Abstract

Despite decades of intense research, our understanding of the correlates of protection against *Salmonella* Typhi (*S*. Typhi) infection and disease remains incomplete. T follicular helper cells (T_FH_), an important link between cellular and humoral immunity, play an important role in the development and production of high affinity antibodies. While traditional T_FH_ cells reside in germinal centers, circulating T_FH_ (cT_FH_) (a memory subset of T_FH_) are present in blood. We used specimens from a typhoid controlled human infection model whereby participants were immunized with Ty21a live attenuated *S*. Typhi vaccine and then challenged with virulent *S*. Typhi. Some participants developed typhoid disease (TD) and some did not (NoTD), which allowed us to assess the association of cT_FH_ subsets in the development and prevention of typhoid disease. Of note, the frequencies of cT_FH_ were higher in NoTD than in TD participants, particularly 7 days after challenge. Furthermore, the frequencies of cT_FH_2 and cT_FH_17, but not cT_FH_1 subsets were higher in NoTD than TD participants. However, we observed that ex-vivo expression of activation and homing markers were higher in TD than in NoTD participants, particularly after challenge. Moreover, cT_FH_ subsets produced higher levels of *S*. Typhi-specific responses (cytokines/chemokines) in both the immunization and challenge phases. Interestingly, unsupervised analysis revealed unique clusters with distinct signatures for each cT_FH_ subset that may play a role in either the development or prevention of typhoid disease. Importantly, we observed associations between frequencies of defined cT_FH_ subsets and anti-*S.* Typhi antibodies. Taken together, our results suggest that circulating T_FH_2 and T_FH_17 subsets might play an important role in the development or prevention of typhoid disease. The contribution of these clusters was found to be distinct in the immunization and/or challenge phases. These results have important implications for vaccines aimed at inducing long-lived protective T cell and antibody responses.

## 1 Introduction

Immunity against enteric bacterial pathogens such as *Salmonella enterica serovar* Typhi (*S*. Typhi) is complex and involves both the innate and adaptive immune systems. Humoral and cell mediated immune responses (CMI) to *S*. Typhi during infection and vaccination have been studied extensively in humans (1, 2). However, the link between these two interrelated arms of the adaptive system has not been studied in *S*. Typhi immunity. T follicular helper (T_FH_) cells are a specialized subset of CD4^+^ T cells that provide vital help to B cells within the germinal centers (GC) of secondary lymphoid organs resulting in the generation of high affinity memory B cells (3). Bonafide T_FH_ cells were first observed in human tonsillar GC and subsequently showed to be present in GC in secondary lymphoid organs (3, 4). T_FH_ express the chemokine receptor CXCR5 (which guides T_FH_ into B cell follicles) and provide critical signals to B cells, including co-stimulatory molecules and cytokines (5). For example, production of interleukin-21 (IL-21) promotes differentiation and class-switching of B cells (5, 6). Furthermore, binding of CD40L (CD154), present on activated T_FH_ cells, to CD40 on B cells triggers a cascade of intracellular signaling events that enhance B cell activation, proliferation, and survival (5, 7). Thus, T_FH_ cells play a pivotal role in facilitating B cell activation, survival, proliferation, maturation, hypermutation, immunoglobulin class switching and plasma cell differentiation, shaping the humoral immune responses against pathogens.

Recent studies have shown that there are substantial numbers (about 15-25% of CD4^+^) of circulating memory T_FH_ cells (cT_FH_) composed of phenotypically and functionally distinct subsets (8, 9). It is widely accepted that circulating T_FH_ in humans exhibit a CD3^+^ CD4^+^ CXCR5^+^ CD45RA^−^ phenotype (8, 10–14). As described and reviewed before, cT_FH_ can be classified into three main subsets, namely cT_FH_1, cT_FH_2, cT_FH_17, based on the expression of CXCR3 and CCR6 markers on the cell surface (15, 16). These cT_FH_ subsets have been shown to have discreet functions. For example, the cT_FH_1 subset lacks the capacity to help naïve B cells but secretes cytokines such as interferon (IFN-)γ, whereas cT_FH_2 cells promote IgG and IgE production and secrete cytokines such as interleukin (IL)-4 and IL-13 (8, 17, 18). cT_FH_17 cells, on the other hand, have been shown to promote efficiently the production of IgG, and particularly IgA, and secrete IL-17A (8). Thus, it is widely accepted that T_FH_2 and T_FH_17 are more efficient helpers than T_FH_1. These three subsets can be further divided into 9 subsets by defining their state of activation. For example, an efficient subset (T_FH_2 or T_FH_17) can be in a quiescent or activated state depending on the expression of markers such as the inducible co-stimulator (ICOS), programmed cell death 1 (PD-1) and C-C chemokine receptor 7 (CCR7) (9). Heretofore it has not been known how cT_FH_ subsets are induced and respond following immunization of humans with oral live attenuated typhoid vaccine Ty21a, and wild type (wt) *S*. Typhi infection.

Infection caused by enteric pathogenic bacteria, particularly those that are human-restricted (e.g., *S*. Typhi) remains a major health problem worldwide, especially in low- and middle-income countries (LMIC). *S.* Typhi, the causative agent of typhoid fever, is an invasive bacteria which causes over 10.9 million cases of typhoid fever leading to around 120,000 fatalities yearly worldwide (19). In addition, *S.* Paratyphi, the causative agent of paratyphoid fever, caused 3-4 million cases resulting between 20-40,000 deaths per year (19). Two distinct types of FDA-licensed typhoid vaccines are available in the United States. Attenuated oral vaccine strain Ty21a generates modest humoral immunogenicity but confers a moderate level of long-lived protection (~60–80%, 5–7 years), depending on the formulation, number of doses administered, and spacing between doses (20–22). Purified unconjugated Vi capsular polysaccharide vaccine is also well tolerated but elicits relatively short-lived protection (2-3 years) (23). The emergence and spread of multi-drug resistant (24, 25) and extensively drug-resistant (XDR) *S*. Typhi strains (26, 27) has renewed interest in existing typhoid vaccines and in the development of new ones that may provide long-lasting protection against *S.* Typhi in endemic areas and for travelers. However, the development of improved typhoid vaccines has been hampered by an incomplete understanding of the immune effector and memory responses responsible for protection (correlates of protection - CoP) from *S.* Typhi infection.

Controlled human infection model (CHIM) studies in which healthy adult participants are intentionally infected with wild-type pathogens to test drugs and vaccines are a particularly relevant model for *Salmonella* infection. In the late 1950s pioneered by Dr. Theodore E. Woodward and continuing through the mid-1970s, investigators at the University of Maryland School of Medicine conducted clinical studies wherein consenting adult participants were experimentally challenged with various strains of *S*. Typhi to study pathogenesis, human immune responses, and to assess the efficacy of various typhoid vaccines. Volunteer challenge studies in the early 1970s first identified that the protection conferred by ingestion of multiple oral doses of freshly-harvested formulations of Ty21a conferred a higher level of protection than had been observed with any previously tested typhoid vaccine. In contrast, oral doses of inactivated typhoid bacilli (28) and of streptomycin-dependent attenuated *S*. Typhi vaccine (29) were far less protective. The early observations in experimental challenge studies in participants led to a clinical development path for Ty21a resulting in its initial licensure and further improvement of the vaccine’s formulation (29). More recently, Dr. Pollard’s group has shown that by using small inocula of virulent *S*. Typhi [~10^3^ or ~10^4^ colony-forming units (CFU)] administered following ingestion of a bicarbonate solution, challenge can be performed safely, with attack rates in excess of 50% (30). The re-establishment of the human challenge model by Oxford Vaccine Group (OVG), UK (30) with the same virulent *S*. Typhi strain as used in the earlier Maryland typhoid challenges provides a unique opportunity to investigate the immune responses following exposure to this pathogen in vaccinated and unvaccinated subjects. In this study, we used peripheral blood mononuclear cells (PBMC) samples obtained from an Oxford study (31) in which participants were vaccinated with Ty21a followed by wt *S*. Typhi challenge (typhoid CHIM) to determine the role of cT_FH_ in *S*. Typhi vaccination and infection.

## 2 Materials and methods

### 2.1 Ethics statement

Participants with no history of typhoid fever and no typhoid vaccination were enrolled in the Oxford University campus. The National Research Ethics Service (NRES), Oxfordshire Research Ethics Committee A (11/SC/0302) approved the protocol for blood collection in the wild-type *S.* Typhi challenge model (31). This study was carried out following the ethical standards laid down in the 1964 Declaration of Helsinki and the principles of the International Conference on Harmonization Good Clinical Practice guidelines (32). Participants were informed about the purpose and risks of the study and written informed consent was obtained from the participants before participation in the study. All blood specimens were processed within 4 h of obtaining the samples.

### 2.2 Participants and challenge

**Figure 1:**
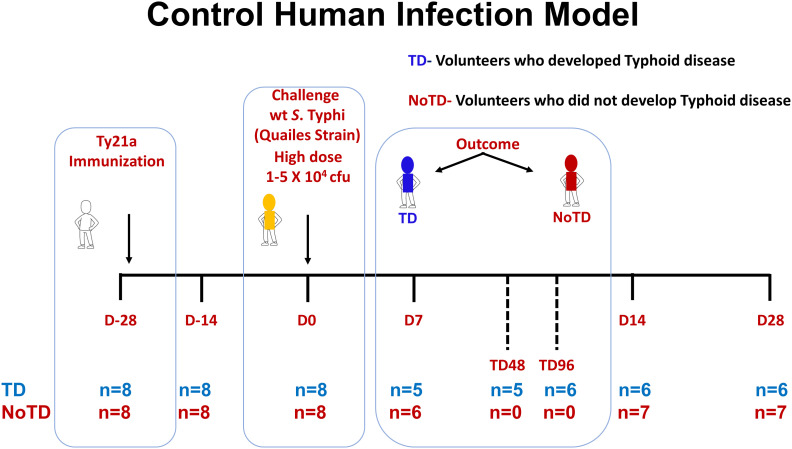
Control human infection model. Schematic of a typhoid control human infection model (CHIM). Participants were recruited and immunized with three doses of the live oral attenuated typhoid vaccine, Ty21a, at days minus 28 (D-28), D-26 and D-24. Participants were then challenged on day 0 (D0) with wt *S*. Typhi (Quailes Strain) at a dose of 1-5 X 10^4^ CFU. At around day 7, some of the participants developed typhoid disease (TD) (blue) while others did not (NoTD) (red). TD48 and TD96 denote PBMC collected 48 or 96 hours after TD diagnosis. On day 14, all participants (TD and NoTD) received antibiotics (Abx). PBMC were collected from multiple time points (shown in red) from baseline (D-28) up to 28 days after challenge. The number of participants studied for each time point in the TD and NoTD groups are shown (n=x).

Sixteen healthy participants aged 18–46 years were screened and recruited by the Oxford Vaccine Group, UK as described before (31). Briefly, participants who had previously received typhoid vaccination, or resided for over 6 months in typhoid-endemic areas or were previously diagnosed with typhoid infection were excluded from this study. Participants were first vaccinated with 3 doses of the live oral attenuated typhoid vaccine, Ty21a and then challenged orally with 1–5 × 10^4^ CFU of wt *S*. Typhi (Quailes strain, an antibiotic susceptible strain) administered after neutralization of gastric acid with NaHCO_3_ as previously described (31). As previously explained, following challenge, some participants developed typhoid disease (TD) as determined by blood culture-confirmed *S*. Typhi bacteremia or development of a fever of ≥38°C for ≥12 h, whilst some participants did not developed typhoid disease (NoTD) (31). Peripheral blood mononuclear cells (PBMC) were obtained from all participants enrolled in this study at various time points as described in **Figure 1**. PBMC were isolated from blood by density gradient centrifugation and cryopreserved in liquid nitrogen following standard techniques (33). PBMC collected before and up to 28 days after challenge were evaluated in the studies included in this manuscript (**Figure 1**).

### 2.3 Generation of autologous target cells

Using each participant PBMC, autologous Epstein–Barr virus (EBV)-transformed lymphoblastoid cell line (B-EBV cells) were generated as previously described (34, 35). Briefly, B-EBV cells were generated by infection of PBMC with EBV particles [supernatant from the B95-8 cell line (ATCC CRL1612)] and cyclosporine (0.5 μg/ml; Sigma-Aldrich, Saint-Louis, MO, USA) for 15–30 days.

### 2.4 *S*. Typhi infection of autologous target cells

Autologous B-EBV cells were incubated with wt *S*. Typhi strain ISP1820 at a multiplicity of infection (MOI) of 7:1 (bacteria:target ratio) for 3 h at 37°C in RPMI free of antibiotics. Cells were washed extensively after the infection with cRMPI and cultured overnight in cRPMI supplemented with gentamicin (150 μg/ml). The efficiency of the *S*. Typhi infection was confirmed by flow cytometry after staining with anti-*Salmonella* common structural Ag (Kierkegaard & Perry, Gaithersburg, MD, USA) as previously described (36).

### 2.5 Stimulation of PBMC

As described previously, PBMC were thawed and rested in cRPMI overnight before stimulation with *S*. Typhi-infected target cells (37–39). For negative and positive controls, uninfected target cells and Staphylococcus enterotoxin B (SEB; 10 μg/ml) were used, respectively. Targets cells were γ-irradiated (6,000 rad) and incubated with PBMC at an effector:stimulator ratio of 7:1 for 2 h in the presence of anti-CD107a (metal conjugated, Fluidigm) monoclonal antibody (mAb). After two hours of incubation, Golgi Stop (0.5 μl; Monensin, BD) and Golgi Plug (0.5 μl, Brefeldin A, BD) were added and the cultures continued overnight at 37°C in 5% CO_2_.

### 2.6 Surface and intracellular staining

After an overnight stimulation, PBMC were stained for mass cytometry analysis as reported before (33, 40, 41). Briefly, cells were first barcoded using CD45 tagged with 141Pr, 154Gd, 156Dy for uninfected, *S*. Typhi-infected EBV stimulated samples and SEB. The samples were then stained for live/dead cell with cisplatin (194/195 Pt), followed by 30 min-incubation with human Fc receptor blocking IgG. Cells were then stained for surface markers and fixed, permeabilized and intracellular staining performed as previously described using the 28-marker panel of anti-human metal-labeled mAbs shown in **Supplementary Table S1**. Finally, within 48 hr of sample labeling, they were stained with an Ir^191/193^ DNA intercalator for cell detection and re-suspended in EQ4 normalization beads (Fluidigm). Data acquisition was performed using a Helios mass cytometer (Fluidigm). Mass cytometry experiments were performed at the Flow Cytometry and Mass Cytometry Core Facility of the University of Maryland School of Medicine Center for Innovative Biomedical Resources (CIBR), Baltimore, Maryland.

### 2.7 ELISA

ELISAs were performed to measure the level of immunoglobulin G (IgG), IgA, and IgM isotype responses to O9:LPS and to H (flagellar antigen) in serum as previously described (30, 42, 43). ELISAs were performed in a set of serum samples obtained at multiple time points (pre-vaccination -D-28-, pre-challenge -D0-, and post-challenge day 28 -D28-) corresponding to the participants (TD *n* = 8, NoTD *n* = 8) in whom the cT_FH_ subsets were evaluated.

### 2.8 Serum bactericidal antibody assay

Serum bactericidal antibody (SBA) assay was performed as described before (43). SBA assays were performed in a set of serum samples obtained at multiple time points (pre-vaccination -D-28-, pre-challenge -D0-, and post-challenge day 28 -D28-) corresponding to the participants (TD *n* = 8, NoTD *n* = 8) in whom the cT_FH_ subsets were evaluated. Briefly, serum samples were de-complemented by heat inactivation and diluted before addition of 200 CFU of log phase *S.* Typhi Quailes strain. *S.* Typhi-specific antibody depleted human complement serum was added to a final complement concentration of 25% and bacteria incubated for 1 h at 37°C with shaking before plating on tryptic soya agar (TSA) plates (Oxoid Ltd., UK) (43). SBA titers were correlated with the mass cytometry measurements for the various cT_FH_ subsets frequencies.

### 2.9 Data analysis

#### 2.9.1 Unsupervised data analysis

**Figure 2:**
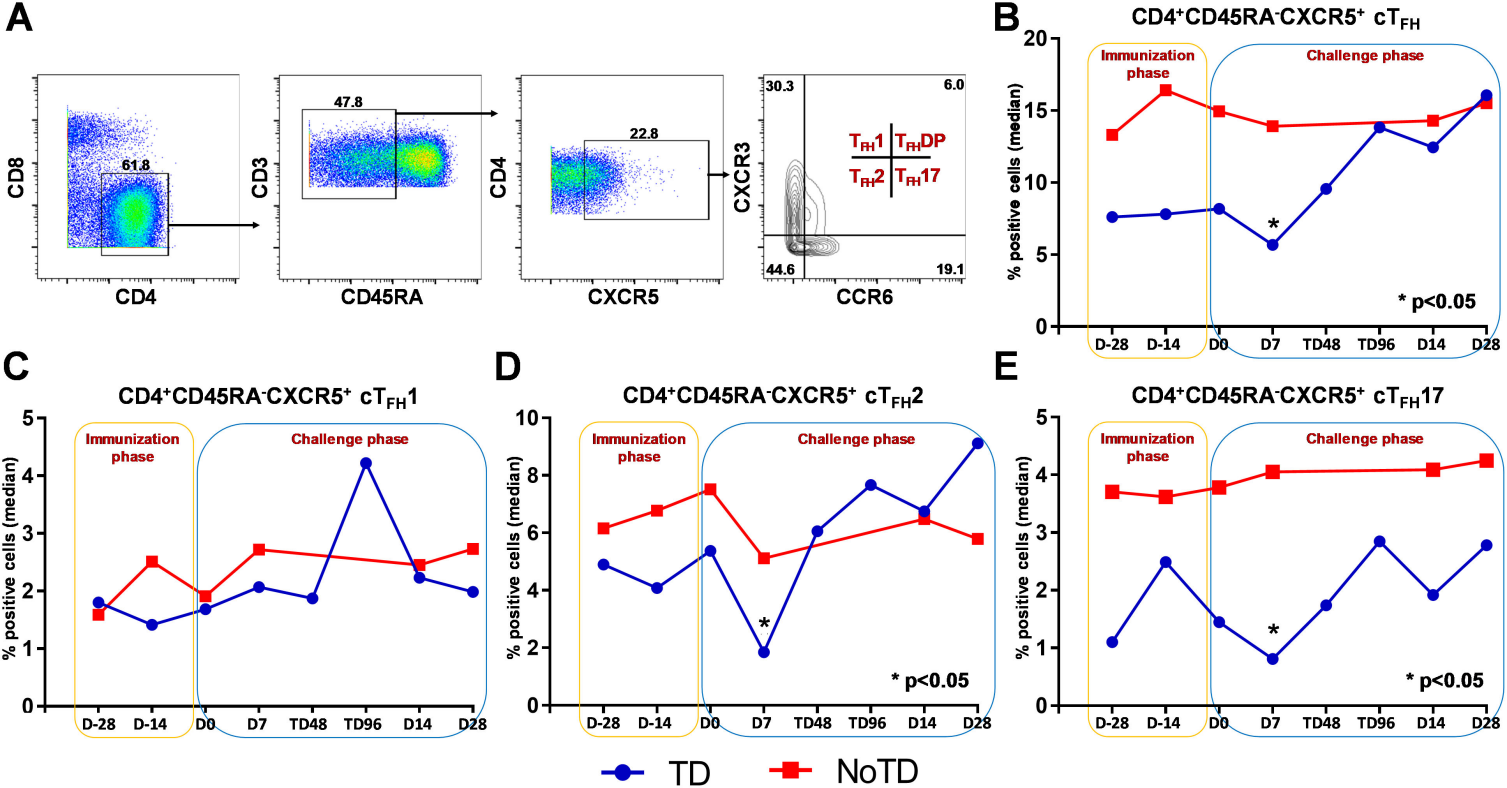
Gating strategy and frequencies of circulating T follicular helper cells (cT_FH_) and their subsets following Ty21a immunization and wt *S.* Typhi challenge. **(A)** Gating strategy showing cT_FH_ (CD4^+^CD45RA^-^CXCR5^+^) in PBMC CD4+ T cells in a representative participant based on expression of CXCR5 and lack of expression of CD45RA. cT_FH_ subsets (cT_FH_1, cT_FH_2, cT_FH_17 and cT_FH_DP) were characterized based on the expression of CXCR3 and/or CCR6 molecules. The frequencies of **(B)** total cTFH, **(C)** cT_FH_1, **(D)** cT_FH_2 and **(E)** cT_FH_17 subsets were measured in the immunization and challenge phases and compared between TD (blue lines) and NoTD (red lines) participants. *Represents significant (p<0.05) differences in frequencies between TD and NoTD at the indicated time points.

All mass cytometry data analyses were performed with FlowJo (version 10.8.1) and its plug-ins such as PeacoQC (version 1.5), UMAP (version 3.1) and PhenoGraph (version 2.5). To ensure maximal data quality, the concatenated data was gated as shown in **Supplementary Figure 1A** to remove doublets, debris and calibration beads. Briefly, the onboard CyTOF software was used to normalize signals and convert data into the Flow cytometry standard (FCS) 3.0 format. FCS files were debarcoded and imported into FlowJo and transformed to arcsinh. Peak Extraction and Cleaning Oriented Quality Control (PeacoQC) plugin (FlowJo) was used to perform quality control on the data in order to evaluate the sample signal for regions of irregularity. PeacoQC Good Events (95.6%) was used for further analysis. The mean absolute deviation (MAD) for all markers was less than 3.1% (**Supplementary Figure S1B**). Gates were generated to detect cT_FH_ and its subsets and to determine their activation status, homing potential and cytokine responses (**Figure 2**). CD3^+^ CD4^+^ CD45RA- CXCR5+ single events were down sampled according to standard guidelines for the assembly of datasets for multidimensional reduction analyses (44, 45) to 3000 events for each volunteers/time points and culture conditions to ensure equal contribution of each sample followed by file concatenation using FlowJo. Then, UMAP (Uniform Manifold Approximation and Projection; version 3.1) was used to perform dimensionality reduction as previously described (45) according to standard guidelines for dimensional reduction analysis (45, 46). UMAPs were created in FlowJo using the plugin UMAP v3.1 (Nearest Neighbors (NN) = 45; Minimal distance = 0.1). Unsupervised clustering was performed using PhenoGraph v2.5 according to standard guidelines to determine the optimal K value (K=180) for clustering analysis of high-dimensional data (47, 48). PhenoGraph clusters were then visualized on the initial UMAP to create a reference map of all automatically detected cT_FH_. The initial UMAP was used for embedding the cT_FH_ subsets to illustrate the distribution of the clusters within the cT_FH_ and to visualize the surface expression of markers in each PhenoGraph cluster. ClusterExplorer (v3.0, FlowJo plug-in) was used to generate heatmaps and perform hierarchical clustering for each cluster at each time points in TD and NoTD participants.

#### 2.9.2 Supervised data analysis

Data were analysed using FlowJO version 10.8.1 after exclusion of doublets, debris and calibration beads and QC by using the PeacoQC plug in (**Supplementary Figures S1A, B**). Singlet CD3^+^ CD4^+^ CD45RA^-^ CXCR5^+^ T cells were evaluated for expression of CXCR3 and CCR6 to determine cT_FH_1, cT_FH_2 and CT_FH_17 and cT_FH_-DP. Subsequently, expression of PD1, ICOS, integrin α4β7, CD62L, CCR7 and CD154 were determined. Cytokines were assessed as shown in the gating strategy (**Supplementary Figure S2A**). *S.* Typhi-specific responses were expressed as net percentage of positive cells (background after culture with uninfected cells were subtracted from the values obtained following culture with *S.* Typhi-infected targets). Boolean gating was performed on CD3^+^ CD4^+^ CD45RA^-^ CXCR5^+^ cT_FH_ for CD107a, Granzyme B, IFNγ, IL-17A and TNFα co-expression and results were graphed as shown (**Supplementary Figures S2B-F**).

#### 2.9.3 Statistical analysis

Data were analyzed using the statistical software GraphPad Prism™ version 7.0 (Graphpad, San Diego, CA, USA) package. Statistical differences in median values between two groups (e.g., TD vs NoTD, D0 vs D7, cluster 1 TD vs NoTD) were determined using Mann–Whitney tests. *P* values < 0.05 were considered significant. Correlation between the frequencies of cT_FH_ subsets and levels of IgG, IgM and IgA antibodies to O9:LPS and to H (flagellar antigen) and SBA were performed using Spearman’s correlation analysis. Consistent with recent recommendations by the American Statistical Association (ASA) (49–51), particularly when analyzing data sets with relatively low numbers of participants, we also indicate trends in the expression of markers or cytokine responses when the statistical analyses yielded values of p ≤ 0.1.

## 3 Results

### 3.1 Total cT_FH_ levels are different in TD and NoTD participants after Ty21a vaccination and wt *S*. Typhi challenge

We determined the role of cT_FH_ in *S*. Typhi infection by using specimens obtained following Ty21a vaccination and a typhoid CHIM. We first characterized cT_FH_ (CXCR5^+^CD45RA^-^CD4^+^) as previously reported (8, 10–14) in PBMC isolated from TD and NoTD participants using the gating strategy shown in **Figure 2A**. Next, we determined and compared the frequencies of total cT_FH_ between TD and NoTD participants (**Figure 2B**). We observed that at baseline (D-28), the frequencies of total cT_FH_ (median %) was higher in NoTD than TD participants (**Figure 2B**). Following Ty21a vaccination, the level of cT_FH_ (median %) remained elevated in NoTD as compared with TD participants at D-14, although these differences were not statistically significant (**Figure 2B**). Following wt *S*. Typhi challenge (D7), total cT_FH_ were significantly (p<0.05) higher in NoTD than in TD participants (**Figure 2B**). However, at later time points, the total cT_FH_ frequencies were not different between NoTD and TD participants (**Figure 2B**).

### 3.2 cT_FH_2 and cT_FH_17 subsets frequencies are higher in NoTD than TD participants

It is widely accepted that cT_FH_ can be classified into well defined, distinct subsets, based on expression of CXCR3 and CCR6 as follows: cT_FH_1 (CXCR3^+^CCR6^−^), cT_FH_2 (CXCR3^−^CCR6^−^), and cT_FH_17 (CXCR3^−^CCR6^+^) (**Figure 2A**) with each having distinct capacities in supporting B cells differentiation and maturation to produce antibodies (8, 9). Thus, we explored whether the frequencies of these specialized cT_FH_ subsets are influenced by Ty21a vaccination and wt *S*. Typhi challenge. Interestingly, no significant differences were noted for cT_FH_1 frequencies between TD and NoTD at baseline (D-28), after Ty21a vaccination (D-14) or following challenge (D7) (**Figure 2C**). In contrast, cT_FH_2 frequencies appear to be higher at baseline (D-28) in NoTD than in TD participants and increased following Ty21a immunization (D-14 and D0) (**Figure 2D**). Of note, after challenge (D7), cT_FH_2 frequencies from NoTD were significantly (p<0.05) higher than those in TD participants (**Figure 2D**). cT_FH_17 frequencies appear to be higher at baseline (D-28), and after Ty21a vaccination (D-14, D0) in the NoTD participants compared with the TD participants (**Figure 2E**). However following challenge (D7), significantly (p<0.05) higher frequencies of cT_FH_17 frequencies were observed in NoTD than in TD participants (**Figure 2E**). To confirm these observations, we performed area under the curve analyses (AUC) to determine whether there were any significant increases in frequencies of the subsets during the vaccination and/or challenge phases. The cumulative AUC data for cT_FH_1 indicate that there were no significant differences between NoTD and TD participants in the vaccination or challenge phases (**Supplementary Figure S3A**). However, cT_FH_2 showed significantly higher (p<0.05) frequencies in NoTD than in TD participants during the vaccination phase, as well as a trend (p ≤ 0.1) to show increases in the challenge phase as measured by AUC (**Supplementary Figure S3B**). Similarly, we noted a trend (p ≤ 0.1) to show significant increases in AUC of cT_FH_17 in NoTD as compared to TD participants in the challenge phase (**Supplementary Figure S3C**). These results suggest that cT_FH_2 and cT_FH_17 might play a role in protection from *S*. Typhi infection.

To assess the effect of vaccination and challenge, we next compared the frequencies of cT_FH_ subsets at D-28 to D-14 for vaccination and D0 to D7 for challenge. No significant differences in frequencies of cT_FH_1, 2, and 17 were detected following Ty21a vaccination, specifically between D-28 and D-14 (**Supplementary Figures S4A-C**). However, we noted that following challenge (D0 to D7), both cT_FH_2 and cT_FH_17 frequencies were lower in TD than in NoTD participants but this did not reach statistical significance. When comparing the levels of cT_FH_2 and cT_FH_17 at D7 after challenge between NoTD and TD, we observed significantly (p<0.05) higher levels of cT_FH_2 and cT_FH_17 in NoTD participants (**Supplementary Figure S4F-G**). No significant differences in cT_FH_1 frequencies were noted following challenge in either TD or NoTD participants (**Supplementary Figure S4E**).

Previous studies have reported that cT_FH_1 is less efficient in the induction of B cells than cT_FH_2 and cT_FH_17 (8). The ratio of cT_FH_2+cT_FH_17 to cT_FH_1 is generally considered an indication of whether there is a shift of cT_FH_ subsets to those that support antibody responses during infection (52). Thus, we determined the effect of Ty21a vaccination and wt *S*. Typhi challenge on the induction of these subsets by comparing D-28 to D-14 for vaccination and D0 to D7 for challenge. We observed that following Ty21a immunization, the ratio of cT_FH_2+cT_FH_17:cT_FH_1 did not show any differences between NoTD and TD participants between D-28 and D-14 (**Supplementary Figure S4D**). However, following wt *S*. Typhi challenge, the ratio of cT_FH_2+cT_FH_17:cT_FH_1 shows a significant (p<0.05) decrease in TD compared with NoTD participants (**Supplementary Figure S4G**). These results suggest that following exposure to wt *S*. Typhi there was a decrease of the percentages of defined cT_FH_ subsets (cT_FH_2 and 17) in TD participants.

### 3.3 cT_FH_ subsets have the potential to home to the gut following Ty21a vaccination and wt *S*. Typhi exposure in TD participants

**Figure 3:**
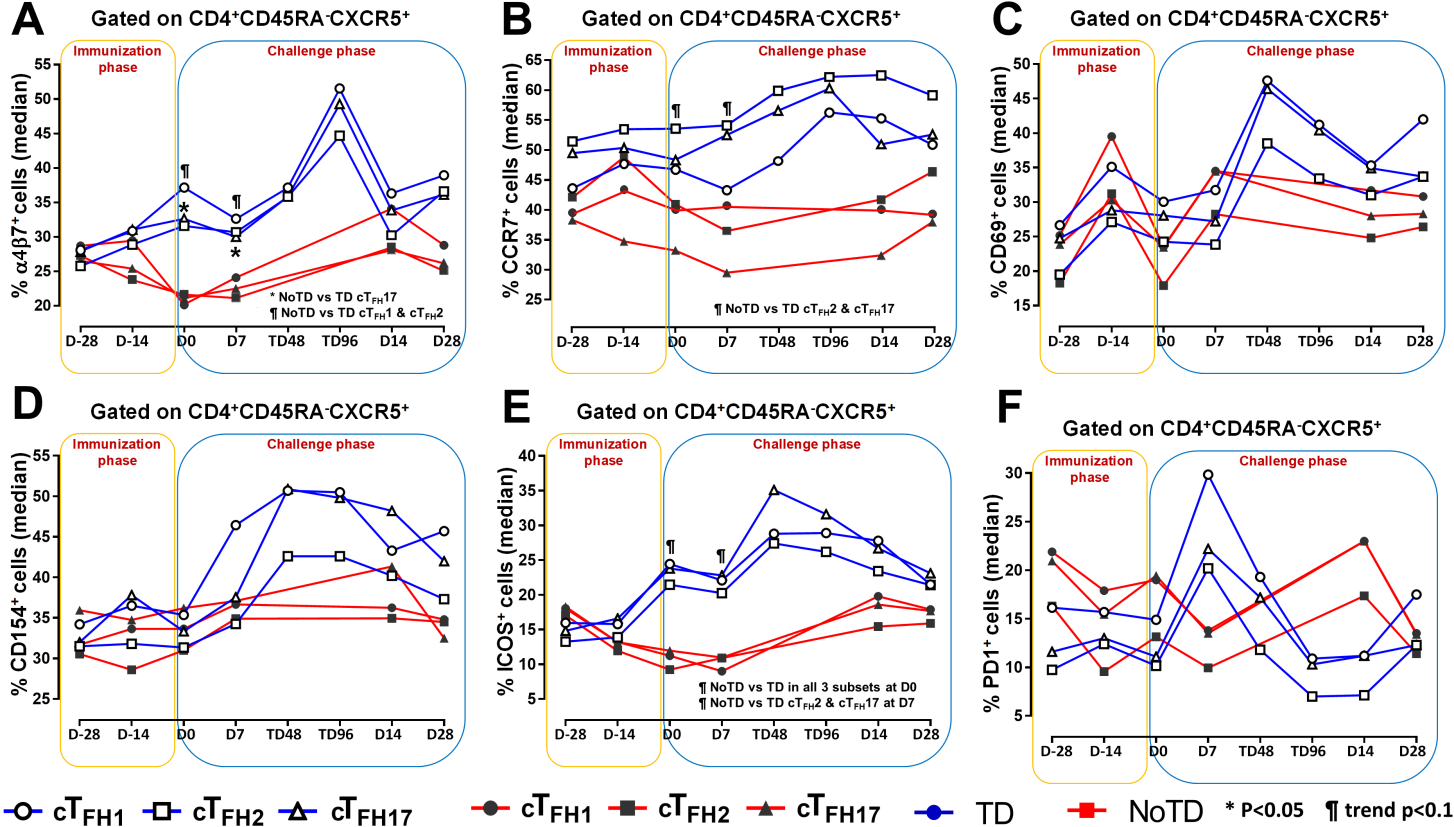
Homing and activation of cT_FH_ subsets following Ty21a immunization and wt *S*. Typhi challenge. Ex-vivo expression of homing markers **(A)** integrin α4β7 and **(B)** CCR7 were measured and compared between cT_FH_ subsets (cT_FH_1, cT_FH_2 and cT_FH_17) in TD (Blue lines) and NoTD (red lines) participants following immunization and wt *S*. Typhi challenge. Similarly, the ex-vivo expression of activation markers, **(C)** CD69, **(D)** CD154 (CD40L), **(E)** ICOS and **(F)** PD1 were assessed and compared between cT_FH_ subsets in TD and NoTD participants following immunization and wt *S*. Typhi challenge. Significant differences between TD and NoTD participants for each subset are represented by *p<0.05. ^¶^ symbols indicate trends (p ≤ 0.1) to show differential responses between TD and NoTD groups for each cT_FH_ subsets.

**Figure 4:**
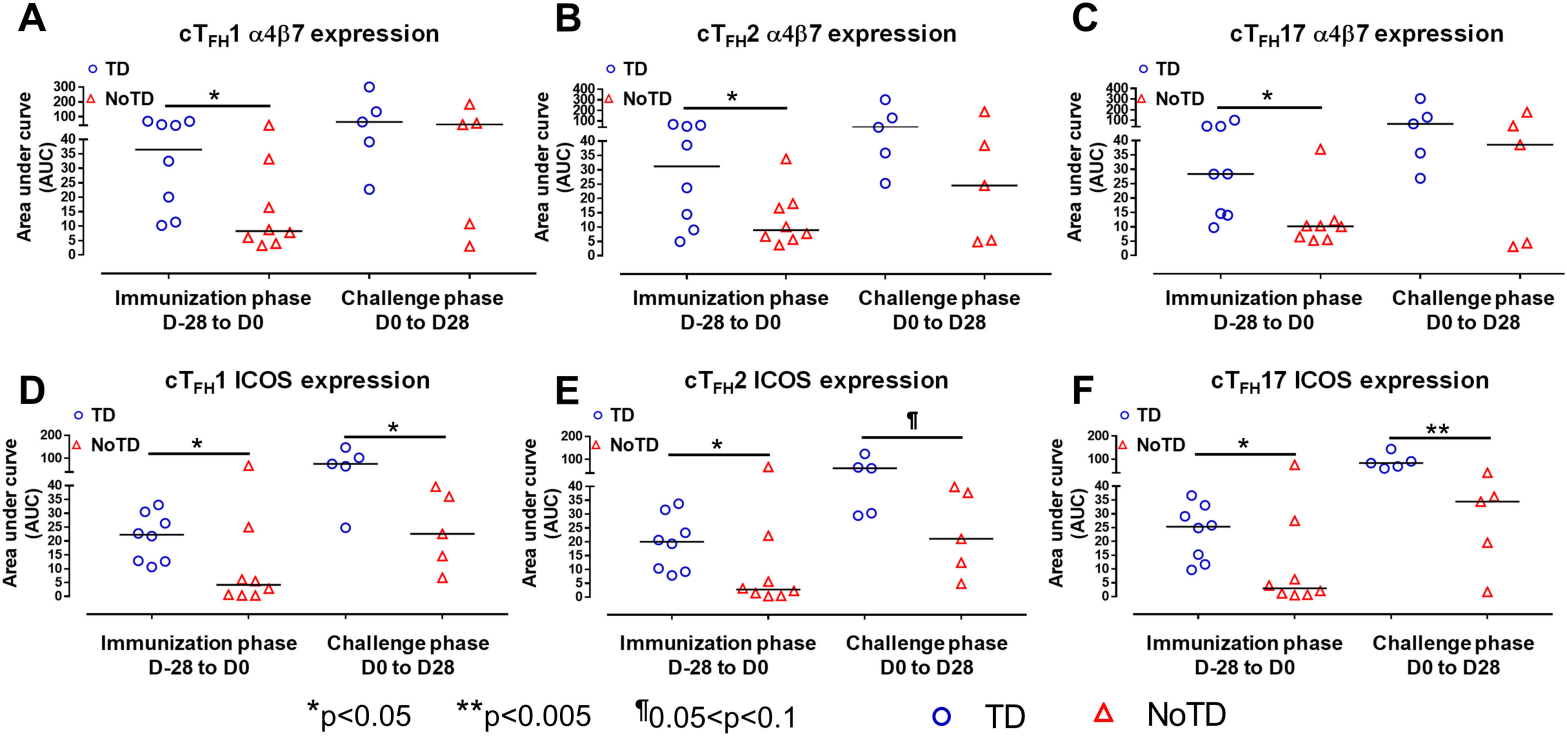
cT_FH_ subsets have increased expression of gut homing and activation markers in TD participants. The areas under the curve (AUC) for each participant was calculated for the immunization phase (D-28 to D0) and for the challenge phase (D0 to D28) for integrin α4β7 expression for **(A)** cT_FH_1, **(B)** cT_FH_2 and **(C)** cT_FH_17. Similarly, AUC were calculated for the ICOS expression for the immunization phase (D-28 to D0) and for the challenge phase (D0 to D28) in **(D)** cT_FH_1, **(E)** cT_FH_2 and **(F)** cT_FH_17. Significant differences between TD and NoTD are represented by *p<0.05 and **p<0.005 respectively. ^¶^ Trends to show significant differences (p ≤ 0.1) between TD and NoTD groups.

To facilitate the interaction between cT_FH_ and B cells, CXCR5 is highly expressed on cT_FH_ to promote the homing of cT_FH_ to lymphoid follicles. Similarly, for cT_FH_ to be effective in other tissues, expression of various homing markers such as integrin α4β7 (promoting migration to intestinal mucosa) (53, 54) and CCR7 (promoting migration to secondary lymphoid tissues) (55) are needed on their cell surfaces. Given that *S*. Typhi invade the intestinal epithelium and disseminate in the lamina propria, we evaluated the capacity of cT_FH_ subsets to home to the intestine and other secondary lymphoid tissues by determining the expression of integrin α4β7 and CCR7, respectively. Interestingly, we observed that integrin α4β7 expression at baseline (D-28) was similar for all subsets regardless of disease status (TD and NoTD) (**Figure 3A**). Following Ty21a immunization, we observed a dichotomy in the expression of integrin α4β7 with higher levels in TD participants than in NoTD, particularly at D0, with significantly (p<0.05) higher levels of integrin α4β7 in cT_FH_17 in TD than in NoTD participants (**Figure 3A**). Moreover, a trend (p ≤ 0.1) to show increases in the percentages of integrin α4β7 was observed in cT_FH_1 and cT_FH_2 in TD compared with NoTD participants at D0 (**Figure 3A**). Following challenge (D7), expression of integrin α4β7 remained significantly (p<0.05) higher in TD than in NoTD for cT_FH_17 and a trend (p ≤ 0.1) to show increases was also observed in TD compared to NoTD participants for the other two cT_FH_ subsets (**Figure 3A**; **Supplementary Tables S2**-**4**). Further analysis of the data by AUC revealed that during the immunization phase, the expression of integrin α4β7 in all three cT_FH_ subsets was significantly (p<0.05) higher in TD than in NoTD participants (**Figures 4A-C**). However, in the challenge phase of the study, no significant differences in integrin α4β7 expression were detected in any of the three cT_FH_ subsets (**Figures 4A-C**). Next, we assessed CCR7 expression on the three cT_FH_ subsets and found that cT_FH_2 and cT_FH_17 appear to express higher levels of CCR7 in TD than in NoTD participants at baseline (**Figure 3B**). Following Ty21a immunization, a trend (p ≤ 0.1) to show increases of CCR7 expression on cT_FH_2 and cT_FH_17 was observed in TD compared with NoTD participants at D0 (**Figure 3B**). Following challenge (D7), trends (p ≤ 0.1) to show increases in the expression of CCR7 were found on cT_FH_2 and cT_FH_17 (**Figure 3B**). Further analysis of the data by AUC revealed that during the immunization phase, the expression of CCR7 showed a trend (p ≤ 0.1) to increase in cT_FH_2 but not cT_FH_1 or cT_FH_17 in TD compared with NoTD participants (**Supplementary Figures S5A-C**). However, during the challenge phase of the study, trends (p ≤ 0.1) to show increases in the expression of CCR7 were observed in TD compared with NoTD participants for the three cT_FH_ subsets (**Supplementary Figure S5A-C**).

### 3.4 cT_FH_ subsets are activated differently following Ty21a vaccination and wt *S*. Typhi challenge

Having established the frequencies and homing potential of cT_FH_ subsets in TD and NoTD participants, we next investigated the activation status of these cT_FH_ subsets following Ty21a vaccination and challenge with wt *S*. Typhi. While both activation markers, CD69 and CD154 (CD40L) were induced in all cT_FH_ subsets following Ty21a vaccination and/or wt *S*. Typhi challenge, no significant differences between TD and NoTD participants were noted at any of the time points (**Figures 3C, D**). To further investigate this phenomenon, we performed AUC analyses. We did not observe any significant differences in the expression of CD69 and CD154 between TD and NoTD participants during the immunization phase (D-28 to D0) for any of three cT_FH_ subsets (**Supplementary Figures S6A-F**). However, during the challenge phase (D0 to D28), we observed trends (p ≤ 0.1) in TD participants to exhibit higher levels of CD69 (cT_FH_2 and cT_FH_17) and CD154 (cT_FH_1 and cT_FH_2) (**Supplementary Figures S6A-F**).

We next determined the level of co-stimulatory molecules (e.g., ICOS and PD1) on the three cT_FH_ subsets in TD and NoTD participants. We observed a clear dichotomy in the expression of ICOS between TD and NoTD participants (**Figure 3E**). At baseline, no differences were observed in ICOS expression between TD and NoTD in the three subsets (**Figure 3E**). However, following Ty21a vaccination, we observed increases in ICOS expression in all three cT_FH_ subsets in TD but not in NoTD participants with a trend (p ≤ 0.1) to exhibit increases at D0 (**Figure 3E**). After challenge (D7), a trend (p ≤ 0.1) to show increases of ICOS expression were found in cT_FH_2 and cT_FH_17 subsets in TD compared with NoTD participants (**Figure 3E**; **Supplementary Tables S2**-**4**). We then performed AUC and observed that during the immunization phase there were significantly (p<0.05) higher levels of ICOS expression in TD than in NoTD participants in all three subsets (**Figures 4D-F**). In the challenge phase, ICOS expression was also higher in TD than in NoTD participants in cT_FH_1 (p<0.05), cT_FH_2 (p ≤ 0.1) and cT_FH_17 (p<0.005) (**Figures 4D-F**). The expression of PD1, however, seems to be higher (not statistically significant) in NoTD than TD in the cT_FH_1 (p ≤ 0.1) and cT_FH_17 (p ≤ 0.1) subsets at baseline (**Figure 3F**). Following Ty21a vaccination, the level of PD1 decreases in NoTD but remains steady in TD participants at D-14 and D0 (**Figure 3F**). Following challenge, PD1 expression was increased in TD but not in NoTD participants (**Figure 3F**). Analysis by AUC showed that there were no significant differences in PD1 expression between TD and NoTD in either the immunization or challenge phases for any of the three cT_FH_ subsets, except for cT_FH_1 in the challenge phase which displayed a trend (p ≤ 0.1) to show higher responses in TD compared with NoTD participants (**Supplementary Figures S5D-F**). Furthermore, we determined the level of CD27, a costimulatory receptor important in T cell function, and CD62L, homing marker to lymphoid tissues, present on cT_FH_ subsets. We observed that CD27 is highly expressed (70-90%) on cT_FH_ subsets at all time points (D-28 to D28) but no significant difference was observed between TD and NoTD participants (**Supplementary Figure S7A**). Similarly, the expression level of CD62L (~50-70%) was high on cT_FH_ subsets at all time points (D-28 to D28), with no significant differences in CD62L expression noted between TD and NoTD participants (**Supplementary Figure S7B**).

### 3.5 Distinct cT_FH_ subsets produced *S*. Typhi specific cytokines following Ty21a vaccination and *S*. Typhi challenge

**Figure 5:**
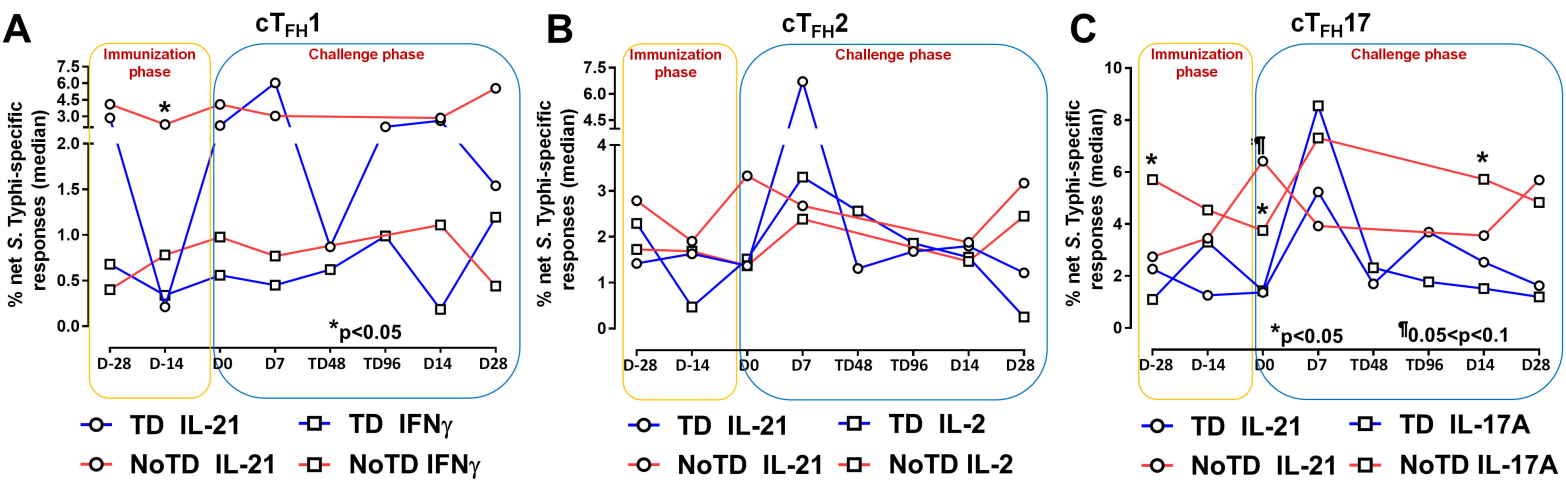
*S*. Typhi-specific responses induced in cT_FH_ subsets following Ty21a immunization and wt *S*. Typhi challenge. *S*. Typhi responses were determined by stimulation of cT_FH_ with **(i)** *S*. Typhi-infected (ST) or **(ii)** non-infected (NI) autologous EBV-B. Net *S*. Typhi responses were calculated as the difference of ST minus NI in the immunization and challenge phases in participants in the TD and NoTD groups. **(A)** Net IL-21 and IFNγ *S*. Typhi responses were measured in cT_FH_1. **(B)** Net IL-21 and IL-2 *S*. Typhi responses were measured in cT_FH_2. **(C)** net IL-21 and IL-17A *S.* Typhi responses were measured in cT_FH_17. Significant differences between TD and NoTD groups are represented by *p<0.05. ^¶^ Trends to show significant differences (p ≤ 0.1) between TD and NoTD groups.

It is well established that cT_FH_ are driven and skewed by an array of cytokines and chemokines that allow for the control of infectious pathogens (56). For example, IL-21 is highly expressed by T_FH_ cell subsets and this cytokine has been shown to play a role in accelerating the development of plasmablasts (3). However, each cT_FH_ subset can be skewed by their secreted set of cytokines. For example, cT_FH_1 secrete mostly Th1 cytokines such as IFN-γ while cT_FH_2 secrete Th2 cytokines such as IL-4 and T_FH_17 secrete mostly IL-17A. Differentiated cT_FH_ cells produced copious amount of IL-2 but, rather than being induced by it, they are inhibited (57). Since cytokine-skewed cT_FH_ can influence the magnitude and quality of humoral responses, we determined *S*. Typhi-specific cytokine responses of cT_FH_ subsets following Ty21a immunization and wt *S*. Typhi challenge. For cT_FH_1 subsets, we first evaluated IL-21 and IFN-γ responses in TD and NoTD participants and observed that at baseline (D-28), no significant differences were observed in IL-21 and IFN-γ levels between TD and NoTD participants in the cT_FH_1 subset following *in vitro* exposure to *S.* Typhi-infected autologous targets (**Figure 5A**). Following Ty21a immunization (D-14), cT_FH_1 subset secrete significantly (p<0.05) lower levels of IL-21 in TD than in NoTD participants. No significant difference in IFN-γ production was observed between TD and NoTD participants (**Figure 5A**). We also determined the level of *S*. Typhi-specific macrophage inflammatory protein (MIP)-1β, tumor necrosis factor (TNF)-α, granzyme B and CD107A responses in cT_FH_1 (**Supplementary Figures S8A, B**). No significant differences in the production of these cytokines or expression of CD107A responses were noted between TD and NoTD participants in the cT_FH_1 subset at baseline (D-28). However, following Ty21a vaccination (D-14), there were significantly (p<0.05) lower levels in MIP-1β (**Supplementary Figure S8A**) and CD107a (**Supplementary Figure S8B**) in TD than in NoTD participants. At D0, there were significantly (p<0.05) lower levels of TNF-α in the cT_FH_1 subset in TD than in NoTD participants (**Supplementary Figure S8A**; **Supplementary Table S2**). Following wt *S*. Typhi challenge (D7), there was an increase in the production of MIP-1β, TNF-α, and granzyme B in the TD group compared with the NoTD group, while CD107a was lower in TD than in NoTD participants (**Supplementary Figures S8A, B**) but none of these responses were statistically significant. Thus, cT_FH_1 subsets produced cytokines important for modulating their environment and influencing B cells to produce distinct antibody isotypes. Similarly, we examined cT_FH_2 *S*. Typhi-specific responses and found no significant differences in *S*. Typhi-specific IL-21 and IL-2 between TD and NoTD participants (**Figure 5B**). Of note, following challenge (D7), the levels of IL-21 were higher in TD than in NoTD participants but did not reach statistical significance (**Figure 5B**). We also determined the levels of *S*. Typhi-specific MIP-1β, TNF-α, granzyme B and expression of CD107A responses in cT_FH_2 of TD and NoTD participants (**Supplementary Figures S8C, D**). No significant differences in the production of these responses were noted between TD and NoTD in cT_FH_2 at baseline (D-28) except for a trend (p ≤ 0.1) to exhibit higher CD107a expression in NoTD than in TD participants (**Supplementary Figures S8C. D**). However, following Ty21a vaccination (D-14), there was a significantly (p<0.05) higher production of cT_FH_2 *S*. Typhi-specific MIP-1β,and TNF-α (**Supplementary Figure S8C**) and CD107a (**Supplementary Figure S8D**) in TD than in NoTD participants. At D0, a trend (p ≤ 0.1) to exhibit higher levels of TNFα (**Supplementary Figure S8C**) and CD107a expression (**Supplementary Figure S8D**) were observed in NoTD compared to TD participants. Following wt *S*. Typhi challenge (D7), we observed trends (p ≤ 0.1) to show increases in the production of MIP-1β, TNF-α, and granzyme B in TD than in NoTD participants (**Supplementary Figures S8C, D**; **Supplementary Table S3**). Next, we investigated cT_FH_17 *S*. Typhi-specific responses and observed that at baseline, significantly (p<0.05) higher production of IL-17A, but not IL-21, was present in NoTD than in TD participants (**Figure 5C**). Following Ty21a vaccination, cT_FH_17 produced higher levels of IL-21 (trend; p ≤ 0.1) and IL-17A (significant; p<0.05) in NoTD than in TD participants at D0 (**Figure 5C**). Following wt *S*. Typhi challenge (D7), we noted an increase in both the production of IL-21 and IL-17A in cT_FH_17 in TD participants, as well as an increase in IL-17 production in NoTD participants (**Figure 5C**). Interestingly, there was significantly (p<0.05) higher production of *S*. Typhi-specific IL-17A in NoTD than TD participants D14 days after challenge (**Figure 5C**). We also determined the level of *S*. Typhi-specific MIP-1β, TNF-α, granzyme B and CD107A responses in cT_FH_17 of TD and NoTD participants (**Supplementary Figures S8E, F**; **Supplementary Table S4**). No significant differences in the production of these responses were noted between TD and NoTD in cT_FH_17 at baseline (D-28), except for a significantly (p<0.05) higher production of *S.* Typhi-specific MIP-1β in NoTD than in the TD group (**Supplementary Figures S8E, F**). However, following Ty21a vaccination (D-14 and D0), there were trends (p ≤ 0.1) to show increases in *S*. Typhi-specific TNF-α production (**Supplementary Figure S8E**) and significantly (p<0.05) higher expression of cT_FH_17 *S.* Typhi-specific CD107a in TD than in NoTD participants (**Supplementary Figure S8F**). Following wt *S*. Typhi challenge, there were no significant differences in cytokine production in cT_FH_17 between TD and NoTD participants (**Supplementary Figures S8E, F**). Thus, like we observed in the cT_FH_1 and cT_FH_2 subsets, cT_FH_17 subsets produced cytokines specifically when exposed to *S.* Typhi antigens following immunization and challenge.

**Figure 6:**
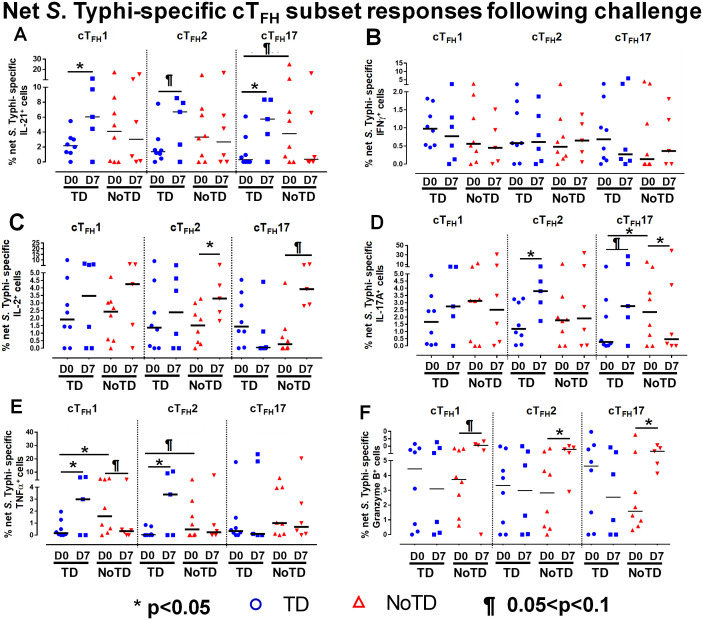
Effect of wt *S*. Typhi challenge on net *S*. Typhi-specific responses elicited by cT_FH_ subsets. Net *S*. Typhi responses of cT_FH_ subsets following wt *S*. Typhi challenge was determined by stimulation of cT_FH_ with **(i)** *S*. Typhi-infected (ST) or **(ii)** non-infected (NI) autologous EBV-B. Net *S*. Typhi responses were calculated by the difference of ST minus NI in PBMC samples from participants in both TD and NoTD groups at D0 (before challenge) and D7 (7 days following challenge). Symbols are individual participants. Net *S*. Typhi responses in **(A)** IL-21, **(B)** IFNγ, **(C)** IL-2, **(D)** IL-17A, **(E)** TNFα and **(F)** granzyme B were determined and compared between days 0 and 7 after challenge and between TD and NoTD groups as indicated by the horizontal bars. Significant differences between days 0 and 7 or between TD and NoTD participants for each subset are represented by *p<0.05. ^¶^ Trends to show significant differences (p ≤ 0.1) between days 0 and 7 and between TD and NoTD groups for each cT_FH_ subset.

To investigate these phenomena in further detail, we focused on the changes of *S*. Typhi-specific responses in cT_FH_ subsets following wt *S*. Typhi challenge by comparing responses at D0 (pre-challenge) and D7 (post challenge) in TD and NoTD participants. Interestingly, in TD participants, there was an increase of IL-21 production in cT_FH_1 (significant, p<0.05), cT_FH_2 (trend, p ≤ 0.1) and cT_FH_17 (significant, p<0.05) from D0 to D7 (**Figure 6A**). No significant differences in IL-21 production were detected from D0 to D7 in any cT_FH_ subset in NoTD participants (**Figure 6A**). It is worth noting that at D0, IL-21 production in cT_FH_ subsets was higher in NoTD than TD participants with a trend (p ≤ 0.1) to show increases observed in cT_FH_17 (**Figure 6A**). No significant differences were detected in IFN-γ production between D0 and D7 in cT_FH_ subsets from either TD or NoTD participants (**Figure 6B**). Similarly, we determined and compared *S*. Typhi-specific IL-2 production between D0 and D7 to evaluate the effect of the *S.* Typhi challenge on cT_FH_ subsets. Interestingly, while cT_FH_1 produced IL-2, there were no significant differences between D0 and D7 regardless of disease status (**Figure 6C**). However, the production of IL-2 was increased from D0 to D7 in cT_FH_2 (significant, p<0.05) and cT_FH_17 (trend, p ≤ 0.1) in NoTD participants (**Figure 6C**). We also determined the production of *S*. Typhi-specific IL-17A at D0 and D7 and found that there were no significant increases in cT_FH_1 regardless of disease status (**Figure 6D**). In contrast, production of *S*. Typhi-specific IL-17A was significantly (p<0.05) higher between D0 and D7 in cT_FH_2 of TD participants (**Figure 6D**). Interestingly, we detected similar trends (p ≤ 0.1) in IL-17A between D0 and D7 in cT_FH_17 of TD participants (**Figure 6D**). In contrast, the production of IL-17A between D0 and D7 was significantly (p<0.05) decreased in cT_FH_17 of NoTD participants (**Figure 6D**). *S*. Typhi-specific TNF-α production was significantly (p<0.05) higher between D0 and D7 in cT_FH_1 and cT_FH_2 of TD but not in NoTD (**Figure 6E**). Interestingly, we noted a trend (p ≤ 0.1) to show decreased TNF-α production between D0 and D7 in cT_FH_1 of NoTD participants (**Figure 6E**). Finally, the production of granzyme B was determined and compared between D0 and D7. We observed higher levels of *S*. Typhi-specific granzyme B production in D0 than D7 in cT_FH_1 (trend, p ≤ 0.1), cT_FH_2 (significant, p<0.05) and cT_FH_17 (significant, p<0.05) in NoTD but not in TD participants (**Figure 6F**). In sum, wt *S*. Typhi challenge alters the production of *S*. Typhi-specific responses and elicited distinct signatures for each cT_FH_ subset in relation to disease status (TD vs NoTD).

Of note, we determined whether *S*. Typhi-specific cT_FH_ responses (CD107a, GzB, IFNγ, IL-17A and TNFα) were either single-producing cells (S) or multifunctional cells (MF) (simultaneously producing two or more cytokine/chemokine). To address this issue, we used Boolean gating (FlowJo) to determine multifunctionality of the effector responses in CD3^+^ CD4^+^ CD45RA^-^ CXCR5^+^ cT_FH_. The results are displayed in **Supplementary Figures S2B-F**. Interestingly, we observed that *S*. Typhi-specific CD107a-associated responses of total cT_FH_ contain higher levels of MF than S in both TD and NoTD volunteers at all time points (**Supplementary Figure S2B**). Similar observations for *S*. Typhi-specific MF and S -associated responses were noted for IFNγ, IL-17A, and TNFα (**Supplementary Figures S2D-F**). However, for Granzyme B (GzB) responses, we observed that both *S*. Typhi-specific S and MF-associated responses display higher levels in TD than NoTD volunteers (**Supplementary Figure S2C**).

### 3.6 Unique cT_FH_ clusters are associated with the prevention or development of typhoid disease

**Figure 7:**
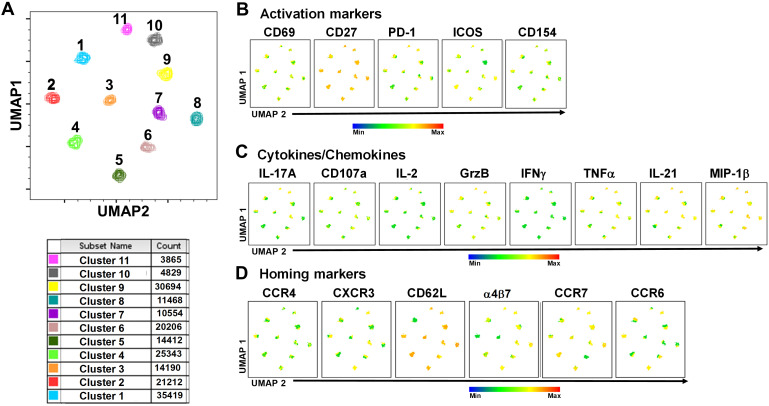
TD and NoTD cT_FH_ grouped into 11 clusters following unsupervised analysis. Concatenated TD and NoTD cT_FH_ at the various time points (D-28 to D28) (same number of events per participant per time point) were found to segregate into 11 clusters with varying levels of activation, homing and cytokines markers. **(A)** Uniform Manifold Approximation and Projection (UMAP) was used to perform dimensionality reductions and plots were generated as described previously (44). Unsupervised clustering was performed using PhenoGraph (57). PhenoGraph clusters were then visualized on UMAP to create a reference map of all automatically detected cT_FH_ subsets. The analyses showed 11 cT_FH_ clusters with different number of events as shown in the table. Based on the UMAP plots, the expression of **(B)** Activation markers (CD69, CD27, PD-1, ICOS, CD154), **(C)** Cytokines and Chemokines (IL-17A, CD107a, IL-2, GranzymeB (GrzB), IFNγ, TNFα, IL-21 and MIP1β), and **(D)** homing markers (CCR4, CXCR3, CD62L, integrin α4β7, CCR7 and CCR6) were evaluated in the 11 cT_FH_ clusters.

To study in further detail the associations between cT_FH_ subsets and typhoid disease we used unsupervised analysis approaches on the rich datasets generated by mass cytometry, by performing a phenotypic analysis on subpopulations gated on CD4+CD45RA-CXCR5+ cT_FH_ using concatenated files from TD and NoTD participants at all time points. First, a dimension reduction step was performed using UMAP (Uniform Manifold Approximation and Projection; version 3.1; FlowJo plugin (45)). Next, unsupervised clustering was performed using PhenoGraph (v2.5; FlowJo plugin) (version 2.7) (48) which resulted into 11 clusters. PhenoGraph clusters were then visualized on the initial UMAP to create a reference map of all automatically detected cT_FH_ subsets with the events numbers in the table below the UMAP for each cluster (**Figure 7A**). We next used this UMAP reference map to visualize and assess the distribution of activation markers (e.g., CD69, CD27, ICOS, PD1 and CD154) (**Figure 7B**), cytokines/chemokines (IL-17A, CD107a expression, IL-2, GzB, IFN-γ, TNFα, IL-21 and MIP-1β) (**Figure 7C**) and homing markers (e.g., CCR4, CXCR3, CD62L, integrin α4β7, CCR7 and CCR6 (**Figure 7D**). We observed that activation markers (CD69, ICOS, PD1, CD154) were distributed in unique patterns across the 11 UMAP clusters, whereas CD27 was expressed in all the clusters (**Figure 7B**). The distribution of cytokines across the 11 clusters also showed distinct patterns (**Figure 7C**). For example, IFN-γ and IL-2 were present in only a few clusters. In contrast, IL-21, MIP-1β, and TNF-α are present on most clusters (**Figure 7C**). Similarly, we noted that homing markers (e.g., integrin α4β7, CCR7, CCR4 and CD62L) are distributed in unique patterns across the 11 clusters. For example, CD62L was expressed in all clusters except for cluster 1 (**Figure 7D**), while CXCR3 and CCR6 markers are expressed in only some cT_FH_ subsets. These results suggest that cT_FH_ clusters have unique combinations of markers that could be important in either Ty21a vaccination or wt *S*. Typhi challenge.

**Figure 8:**
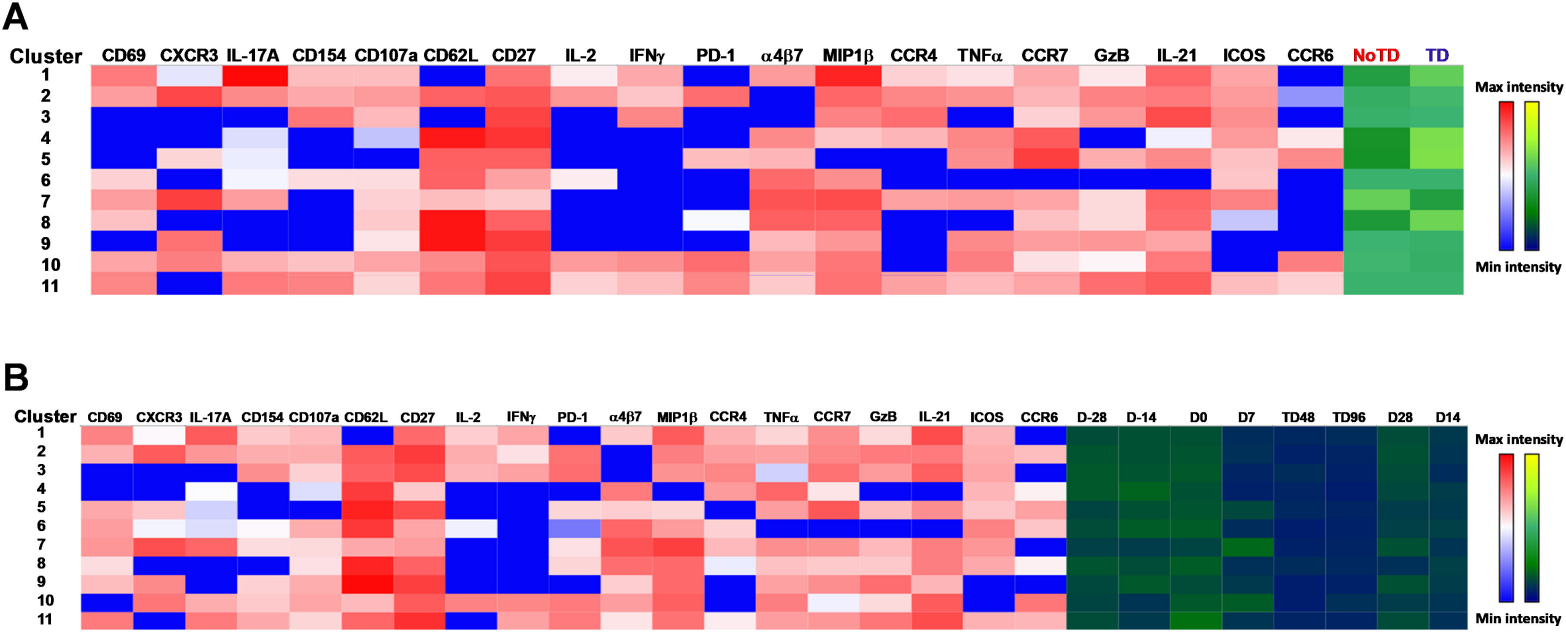
cT_FH_ clusters are present differentially in TD and NoTD participants and during Ty21a vaccination and *S*. Typhi challenge. **(A)** Clusters expressing various markers at different levels of expression as shown by a Red-Blue color scheme (red-maximum expression; blue-low/no expression). The frequencies of the clusters are compared between TD and NoTD participants and their intensity shown by a Yellow-Black/Dark Blue color scheme (Yellow-maximum frequency; black/dark blue-low/no frequency). **(B)** Comparison of the frequencies of the 11 clusters of cT_FH_ across the various time points (D-28, D-14, D0, D7, TD48, TD96, D14 and D28) as shown by a Yellow-Black/Dark Blue color scheme (Yellow-maximum frequency; black/dark blue-low/no frequency).

**Figure 9:**
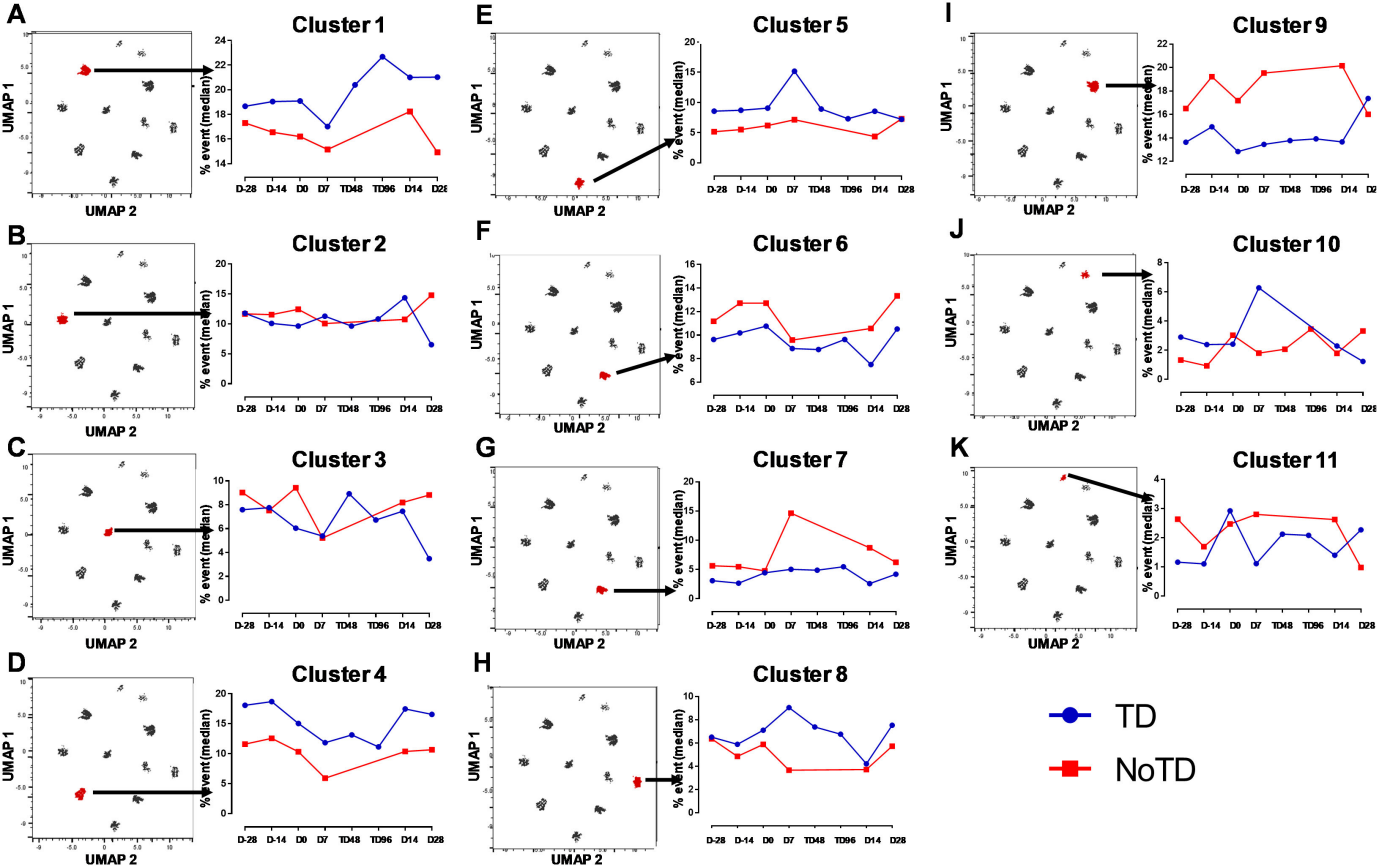
Distinct clusters are associated with the development of typhoid disease (TD) or lack of development of typhoid disease (NoTD). Each single cluster **(A-K)** (1–11) frequencies (% events) were evaluated and compared between TD (Blue line) and NoTD (red line) at all time points (D-28, D-14, D0, D7, TD48, TD96, D14 and D28). Arrows indicate the kinetics of which of the 11 cT_FH_ clusters is being evaluated in each panel.

Next, we compared the expression of phenotypic, homing and activation markers of each of the 11 clusters as shown by the red color for maximum expression and blue for minimum expression (**Figure 8A**). For example, cluster 1 was CD62L- CCR6- CXCR3 dim/- and positive for all other markers including IL-17A, MIP-1β (**Figure 8A**). Furthermore, we used the Markers Enrichment Model (MEM) (58) to determine the main phenotypes of the 11 clusters of cT_FH_ (**Supplementary Table S5**). We observed that each cluster has a unique phenotype and some of the markers are more abundant than others (**Supplementary Table S5**). We then determined the frequencies of each cluster in TD and NoTD participants and observed that there were various clusters that were present at higher frequencies in TD than in NoTD participants as shown by the yellow color intensity (**Figure 8A**). For example, clusters 1, 4, 5 and 8 were present at higher in frequencies in TD than in NoTD participants (**Figure 8A**). However, these differences in cluster frequencies includes all time points. Of note, we use individual histogram to show the expression of the various markers in TD and NoTD from a representative cluster (cluster 4 in **Figure 8A**) is shown in **Supplementary Figure S9**. Thus, we next determined the frequencies of each cluster at each time point (D-28, D-14, D0, D7, TD48, TD96, D14 and D28) (**Figure 8B**). We observed that each cluster is present in higher frequencies at particular time points (**Figure 8B**). For example, following Ty21a vaccination at D-14, clusters 4, 6 and 9 are present at higher frequencies (**Figure 8B**). However, these differences might be masked by the clinical status (TD and NoTD). Thus, to interpret how the clusters varies between TD and NoTD at the various time points, we determined and compared the frequencies of each cluster (1–11) gated on either TD (blue line) or NoTD (red line) at each time point (**Figures 9A-K**). Interestingly, we observed that there were some clusters that were higher in TD than NoTD (e.g., clusters 1, 4, 5, 8), while other clusters (e.g., clusters 6, 7, 9) were higher in NoTD than TD (**Figures 9A-K**). In addition, there were clusters that showed increases following wt *S*. Typhi challenge (e.g., clusters 7, 10) (**Figures 9A-K**). These results suggest that distinct clusters could be associated with either the development or prevention of typhoid disease. Furthermore, there may be distinct clusters which could be elicited following Ty21a immunization or wt *S*. Typhi challenge.

### 3.7 cT_FH_ subsets contained unique clusters that may be involved in the prevention or development of typhoid disease

**Figure 10:**
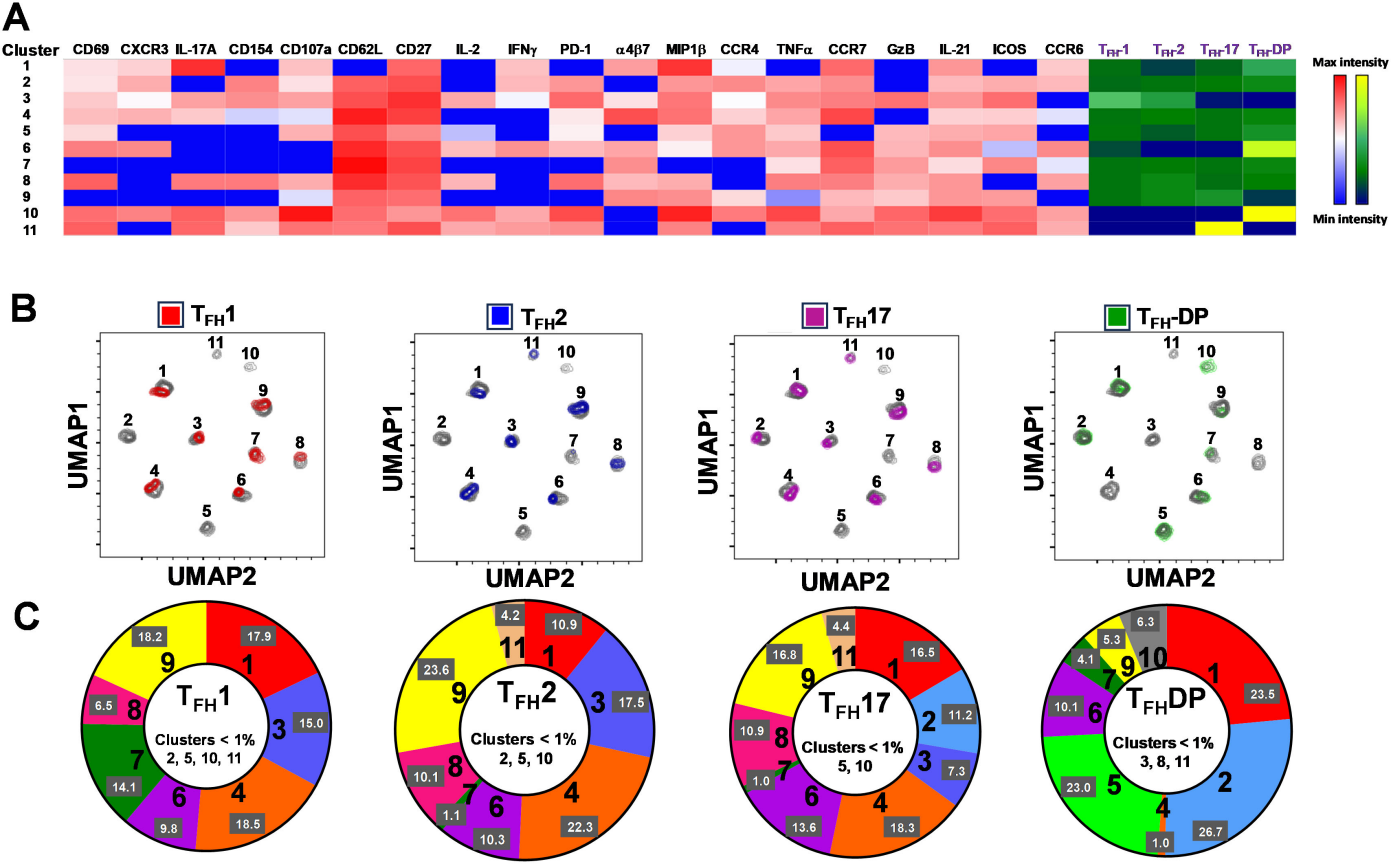
Clusters are differentially expressed, and in some cases present exclusively in individual cT_FH_ subsets. **(A)** Phenotypic markers in the individual clusters showed varying levels of expression of the various homing and activation markers as shown by a Red-Blue color scheme (red-maximum expression; blue-low/no expression). The frequencies of the clusters are compared between cT_FH_1, cT_FH_2, cT_FH_17 and cT_FH_-double positive (DP) subsets as shown by a Yellow-Black/Dark Blue color scheme (Yellow-maximum frequency; black/dark blue-low/no frequency). **(B)** Frequencies of the 11 clusters of cT_FH_ in each of the four cT_FH_ subsets, as defined by CXCR3 vs CCR6, were compared on UMAP plots. **(C)** Frequencies (% events) of the 11 clusters for each cT_FH_ subset are shown in a representative volunteer (Ox 2001).

**Figure 11:**
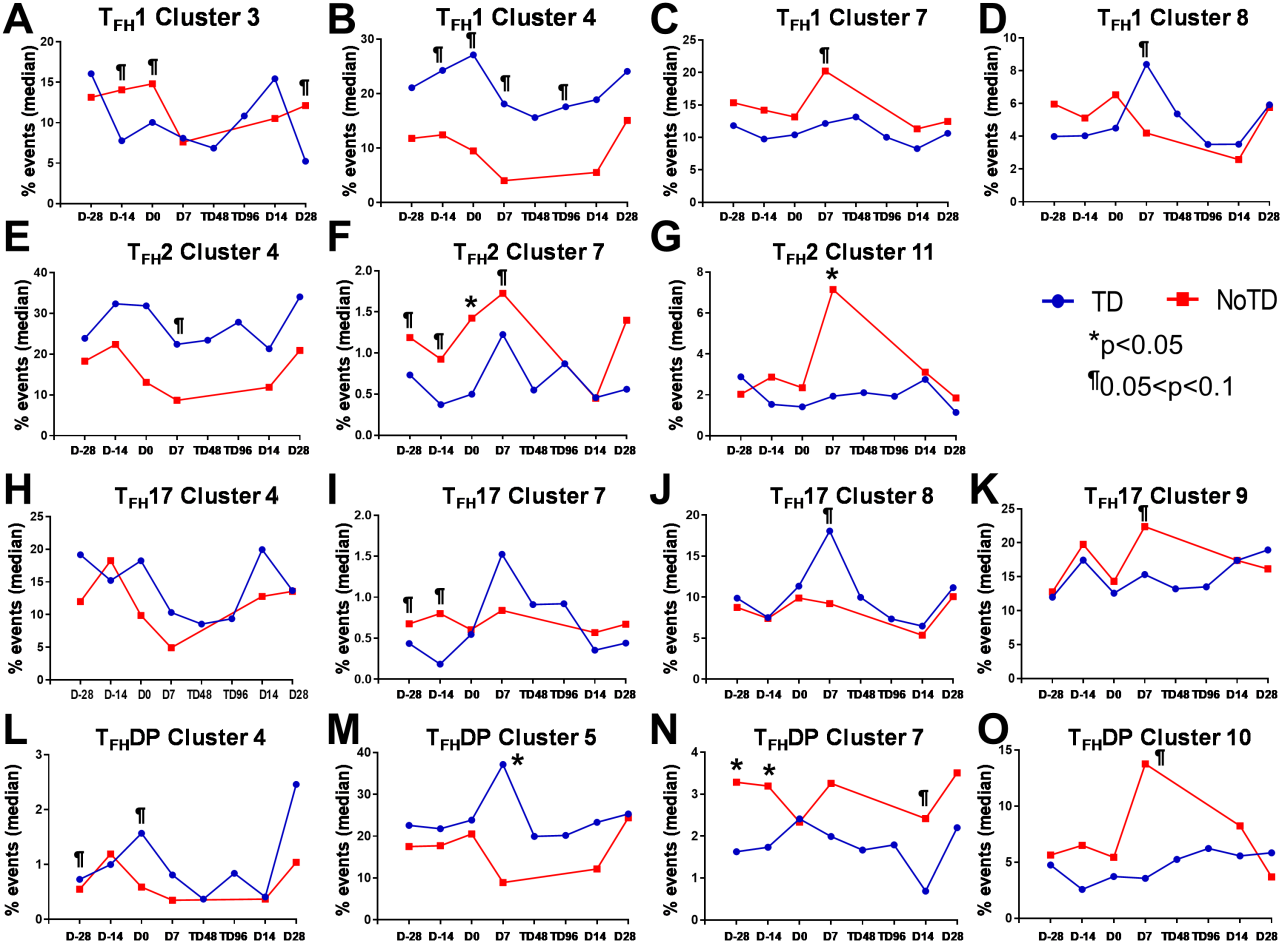
Each cT_FH_ subset has distinct clusters that are associated with TD or NoTD in the vaccination and/or challenge phases. For each cT_FH_ subset, the kinetics of cluster (1-11) frequencies (% events) were evaluated and compared between TD (blue lines) and NoTD (red lines) at all time points (D-28, D-14, D0, D7, TD48, TD96, D14 and D28). For cT_FH_1 subsets, **(A-D)** clusters 3, 4, 7, 8; T_FH_2 **(E-G)** clusters 4, 7, 11; T_FH_17 **(H-K)** clusters 5, 7, 8, 9; and T_FH_-DP **(L-O)** clusters 4, 5, 7 and 10 were evaluated for their frequencies in TD (blue) and NoTD (red) at all time points. Significant differences between TD and NoTD are indicated by *p<0.05. ^¶^ Trends to show significant differences (p ≤ 0.1) between TD and NoTD groups.

In **Figure 2A** we show, as widely reported in the literature (8, 9, 16), that cT_FH_ can be classified into four subsets based on the expression of CXCR3 and CCR6, i.e., cT_FH_1, cT_FH_2, CT_FH_17 and cT_FH_-DP (double positive). We applied this hierarchical analysis to the clustering analysis and determined that each cluster is present at defined frequencies among cT_FH_ subsets (**Figure 10A**). For example, cluster 11 was present mostly in cT_FH_17 as indicated by yellow coloring (**Figure 10A**). Similarly, clusters 6 and 10 were highly represented in the cT_FH_-DP subset (**Figure 10A**). However, these data represent TD and NoTD participants and all time points combined. Thus, we next visualized the cT_FH_ subsets by embedding them onto the UMAP reference map which shows that cT_FH_1 subset is present in clusters 1, 3, 4, 6, 7, 8 and 9, cT_FH_2 in clusters 1, 3, 4, 6, 8, 9 and 11, cT_FH_17 in clusters 1, 2, 3, 4, 6, 8, 9 and 11 and cT_FH_-DP in clusters 1, 2, 5, 6, 7, 9, and 10 (**Figure 10B**). We also observed that some clusters are unique to some of the subsets. For example, cluster 5 is unique to cT_FH_-DP while cluster 11 is composed exclusively of cT_FH_2 and cT_FH_17 (**Figure 10B**). In addition, we observed that the distribution of the 11 clusters varies within each cT_FH_ subset as shown by the pie chart for a representative volunteer (**Figure 10C**). We next deconvoluted the data that involves each cT_FH_ subset with the clusters of interest based on the results shown in **Figure 10** and apparent differences between the TD and NoTD groups to evaluate the effect of Ty21a vaccination and wt *S*. Typhi challenge. Note that clusters were excluded if their frequencies (median % events) were less than 1% at any time point following comparison between TD and NoTD participants. We observed that there were 4 clusters of interest (Clusters 3, 4, 7 and 8) for cT_FH_1 (**Figures 11A-D**). At baseline (D-28), no significant differences were observed in the frequencies of cT_FH_1cluster 3 between TD and NoTD participants, while following Ty21a immunization (D-14 and D0), we observed a trend (p ≤ 0.1) to show increases in the frequencies of cT_FH_1 in NoTD compared to TD participants (**Figure 11A**). Following challenge (D7), the frequency of cT_FH_1 cluster 3 in NoTD decreases and was not significantly different from TD participants (**Figure 11A**). At D28, however, we again observed a trend (p ≤ 0.1) to show increases in the frequencies of this cT_FH_1 cluster 3 in NoTD compared to TD participants (**Figure 11A**). Remarkably, cT_FH_1 cluster 4 was higher in frequency (almost twice) in TD than in NoTD participants at all time points (**Figure 11B**), with trends (p ≤ 0.1) observed after Ty21a immunization (D-14 and D0) and after wt *S*. Typhi challenge (D7 and TD96) (**Figure 11B**). In contrast, cT_FH_1 cluster 7 frequencies were higher in NoTD (red line) than in TD (blue line) at all time points (**Figure 11C**), with trends (p ≤ 0.1) observed in NoTD than TD following challenge (D7) (**Figure 11C**). cT_FH_1 cluster 8 exhibited higher frequencies at baseline and following Ty21a vaccination in NoTD than in TD participants but differences were not statistically significant (**Figure 11D**). However, following wt *S*. Typhi challenge (D7), cluster 8 showed a trend (p ≤ 0.1) to be higher in TD than in NoTD participants (**Figure 11D**). We also compared TD and NoTD participants for all remaining cT_FH_1 clusters and noted no significant differences in frequencies between the TD and NoTD groups in any phase of the study.

Next, we evaluated cT_FH_2 clusters and found that clusters 4, 7 and 11 were of interest (**Figures 11E–G**). cT_FH_2 cluster 4 exhibited higher frequencies in TD than in NoTD participants at all time points (**Figure 11E**), with a trend (p ≤ 0.1) to be higher in TD than in NoTD participants following challenge (D7) (**Figure 11E**). In contrast, cT_FH_2 cluster 7 frequencies were higher in NoTD than TD at most time points (D-28, D-14, D0, D7 and D28) (**Figure 11F**), showing a trend to be higher in NoTD than in TD participants at baseline (D-28; p ≤ 0.1), after Ty21a vaccination (D-14, p ≤ 0.1) and following challenge (D7, p ≤ 0.1) but with significant differences observed at D0 (p<0.05) (**Figure 11F**). No significant differences were observed in the frequencies of cT_FH_2 cluster 11 between TD and NoTD participants at baseline and after Ty21a vaccination (**Figure 11G**). However, following challenge, we found that there was a significant (p<0.05) increase in the frequencies of cT_FH_2 cluster 11 in NoTD compared to TD participants (**Figure 11G**). No other trends or statistically significant differences were observed in the remaining cT_FH_2 clusters.

We also examined cT_FH_17 clusters and found that clusters 4, 7, 8 and 9 were of interest (**Figures 11H-K**). No significant differences in cT_FH_17 cluster 4 frequencies between TD and NoTD participants were detected at any time points (**Figure 11H**). cT_FH_17 cluster 7 showed trends (p ≤ 0.1) to higher frequencies in NoTD than in TD participants at baseline (D-28) and following Ty21a immunization (D-14) (**Figure 11I**) but not following challenge (D7). A trend (p ≤ 0.1) to show increased frequencies in TD participants was observed in cluster 8 after challenge (D7) (**Figure 11J**). In contrast, a trend (p ≤ 0.1) to show increased frequencies in NoTD participants was observed in cT_FH_17 cluster 9 after challenge (D7) (**Figure 11K**). No significant differences were observed in the remaining cT_FH_17 clusters.

Finally, we investigated clusters associated with cT_FH_ CXCR3^+^CCR6^+^ (cT_FH_-DP) and found that clusters 4, 5, 7 and 10 were clusters of interest (**Figures 11L-O**). cT_FH_-DP cluster 4 showed a trend (p ≤ 0.1) to show higher frequencies in TD than in NoTD participants at baseline (D-28) and following Ty21a immunization (D0), but not after challenge (**Figure 11L**). For cT_FH_-DP cluster 5, higher frequencies were observed in TD participants, which became significant (p<0.05) after challenge (D7) (**Figure 11M**). However, for cT_FH_-DP cluster 7, we noted that its frequencies were significantly (p<0.05) higher in NoTD participants at baseline (D-28) and following Ty21a immunization (D-14), but with a trend (p ≤ 0.1) observed at D14 following challenge (**Figure 11N**). Similarly, for cT_FH_-DP cluster 10, we observed higher frequencies (no statistical significance) in NoTD than in TD participants at most time points, but with a trend (p ≤ 0.1) observed 7 days following challenge (D7) (**Figure 11O**). Thus, each cT_FH_ subset includes clusters of particular interest that are associated with either in protection or the development of typhoid disease.

### 3.8 Unique clusters have activated, homing, and cytokine signatures that are associated with the prevention or development of typhoid disease

**Figure 12:**
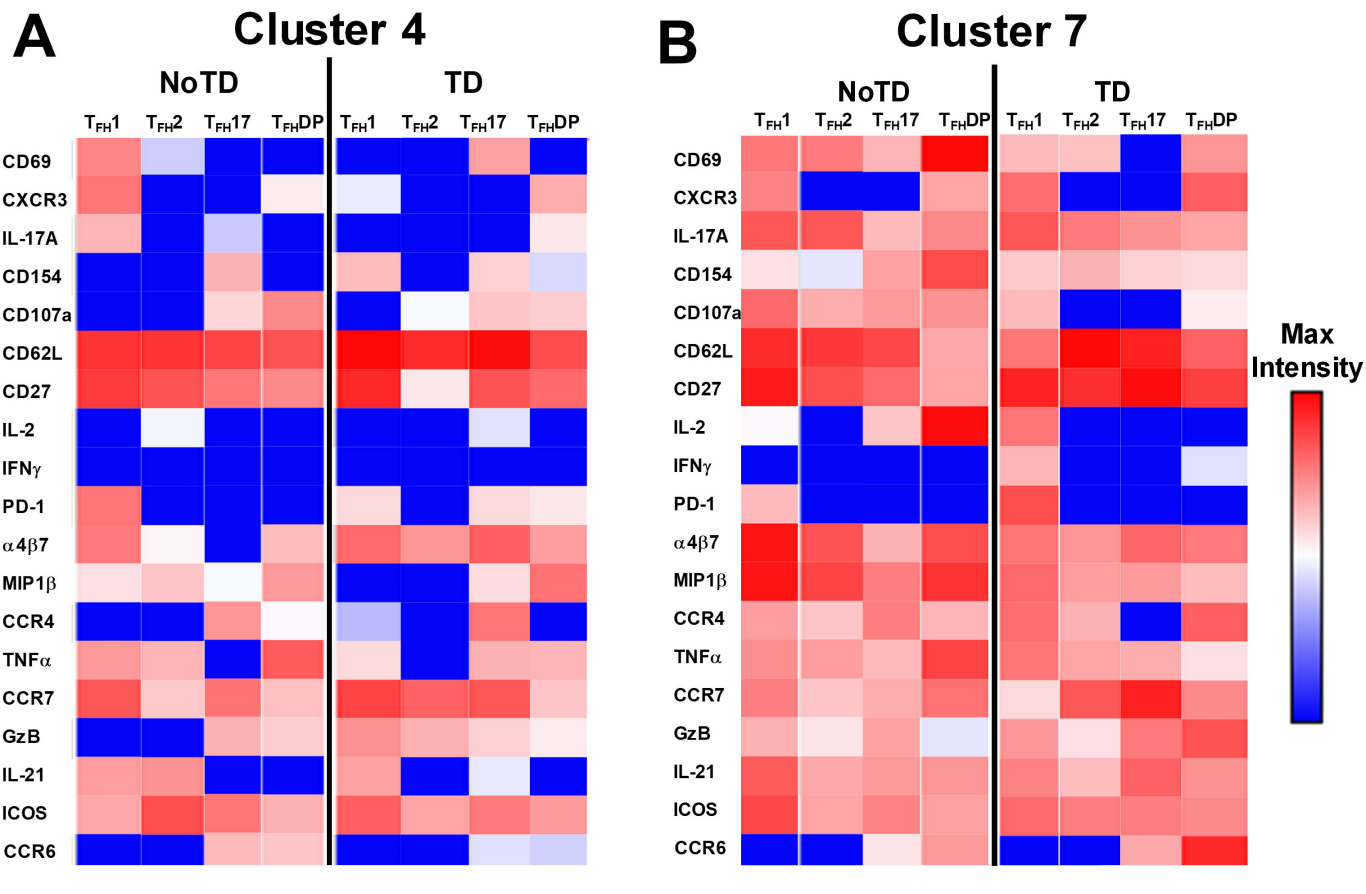
Clusters 4 and 7 are associated with the development or protection from typhoid disease and are present in all subsets. **(A)** Cluster 4 is present in T_FH_1, T_FH_2, T_FH_17 and T_FH_-DP and their phenotypic, homing, and functional markers are expressed in each cT_FH_ subsets at different levels as shown by the heatmap Red-Blue color scheme (Maximum level-red and minimum level-blue) in TD and NoTD. **(B)** Cluster 7 is present in T_FH_1, T_FH_2, T_FH_17 and T_FH_-DP and their phenotypic, homing, and functional markers are expressed in each cT_FH_ subset at various levels as shown by the Red-Blue color scheme (Maximum level-red and minimum level-blue) in TD and NoTD.

The phenotype of the clusters that might be involved in Ty21a vaccination and *S*. Typhi infection was examined in further depth in **Figures 12A, B**. Two clusters (4 and 7) have been shown above (**Figures 10**, **11**) to be present in all four subsets of cT_FH_ and may play a role in the development of typhoid disease. Interestingly, cluster 4 is present in all four subsets contributing major proportions to cT_FH_1 (18-30%) and cT_FH_2 (20-30%) and minor components to cT_FH_17 (5-20%) and cT_FH_-DP (1-2%). We observed that cluster 4 is present at higher frequencies in TD than in NoTD participants at all time points of the study (**Figure 11**) in the four cT_FH_ subsets. These results suggest that cluster 4 may be associated with the development of typhoid disease in participants who developed TD following wt *S.* Typhi challenge. Thus, we examined closely the phenotype of cluster 4 in TD and NoTD participants. Heatmaps showed that there were major differences between TD and NoTD participants (**Figure 12A**). In particular, as denoted by activation markers and cytokine production, we found that cT_FH_ subsets in TD participants cluster 4 (e.g., cT_FH_17 CD69+ PD-1+ ICOS+ IL-2+ TNF-α+ IL-21+ and cT_FH_1 CD69- CD154+ Granzyme B(GzB)+ PD-1+ ICOS+) were highly activated in TD as compared to NoTD participants (**Figure 12A**). Of note, the homing marker integrin α4β7 (gut), but not CCR4 (inflammatory) or CCR7 (lymph node), were expressed at higher levels in cT_FH_2 and cT_FH_17 subsets in TD compared to NoTD participants (**Figure 12A**). Interestingly, the cytokine patterns of cluster 4 in NoTD participants indicate more production of MIP-1β in cT_FH_1 and cT_FH_2 subsets and, importantly, IL-21 in the cT_FH_2 subset (**Figure 12A**).

In contrast, cluster 7, although present in all four cT_FH_ subsets, the frequencies were higher in cT_FH_1 (10-20%) with minor frequencies in cT_FH_2 (0.5-2%) and cT_FH_17 (0.5-2%) and cT_FH_-DP (1-5%). We have described above (**Figure 11**) that cluster 7 is present at higher in frequencies in NoTD than in TD participants at most time points of the study, suggesting that cluster 7 may be associated with the prevention of typhoid disease. Thus, we examined closely the phenotype of cluster 7 in TD and NoTD participants (**Figure 12B**). Heatmaps showed that there were minor differences between the TD and NoTD groups based on the expression of activation markers CD69, CD154, PD1, ICOS, and CD27 in cluster 7 of cT_FH_1 and cT_FH_-DP subsets (**Figure 12B**). However, there were some differences in the activation patterns in cluster 7 of cT_FH_2, cT_FH_17 and cT_FH_-DP (e.g., CD69^+^) in NoTD compared to TD participants (**Figure 12B**). The homing markers integrin α4β7 (gut), CCR4 (inflammatory) and CCR7 (lymph nodes) were highly expressed on all cT_FH_ subsets in both TD and NoTD participants, except for CCR4 in T_FH_17 in TD participants (**Figure 12B**). Interestingly, the cytokine patterns on cluster 7 shows that IL-17A, MIP-1β, TNF-α, granzyme B and IL-21 are highly expressed in both TD and NoTD participants (**Figure 12B**). However, IFN-γ expression was absent from all subsets associated with cluster 7 in NoTD participants while IFN-γ associated with cluster 7 in TD participants was expressed in cT_FH_1 and cT_FH_-DP (**Figure 12B**). Furthermore, IL-2 associated with cluster 7 was only present on cT_FH_1 of TD participants while in NoTD participants, cluster 7 in cT_FH_17 and cT_FH_-DP but not cT_FH_1 and cT_FH_ 2 produced IL-2 (**Figure 12B**). These contrasting phenotypes present in cluster 7 between TD and NoTD participants may account for its association with the absence of typhoid disease.

**Figure 13:**
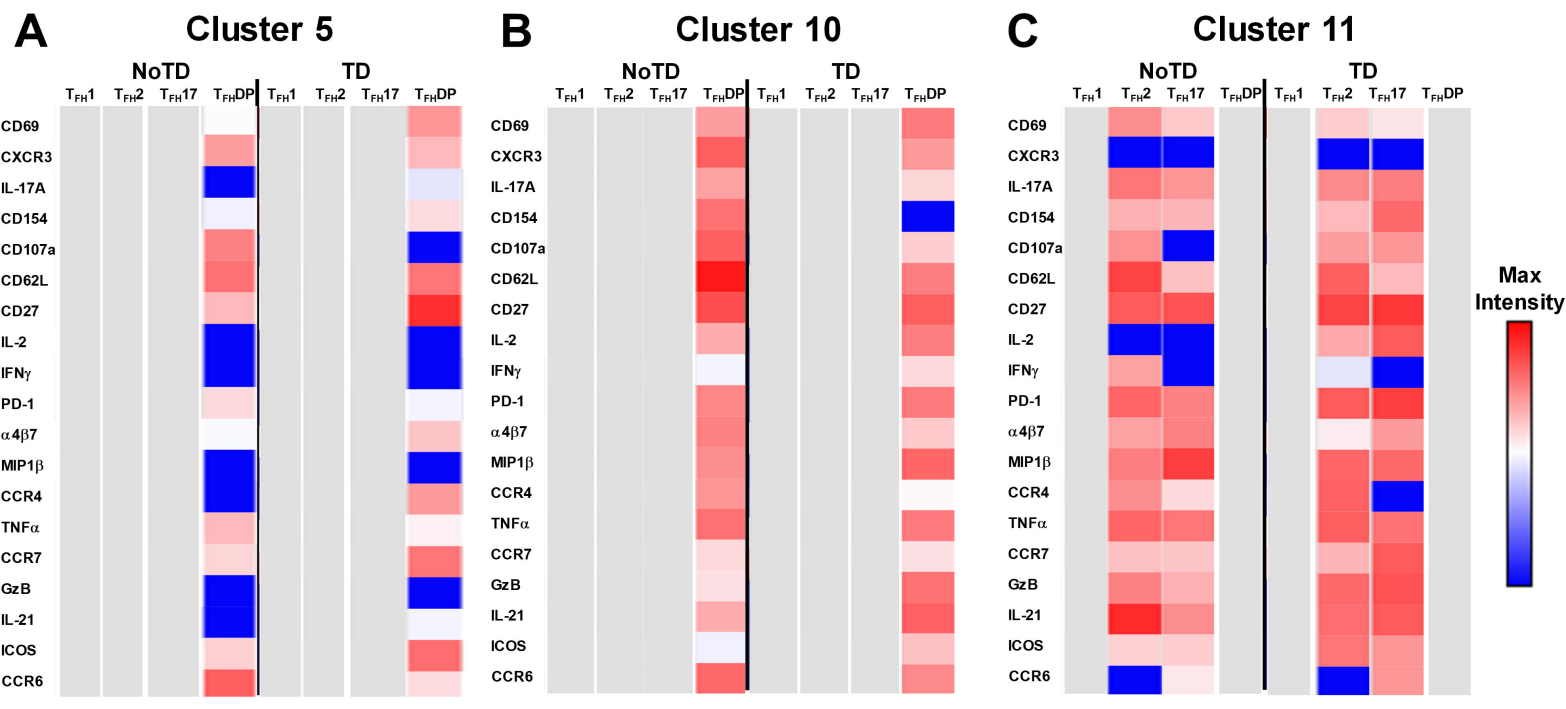
Unique clusters defined functional cT_FH_ subsets that are associated with the development of typhoid disease. **(A)** cluster 5 is mostly present in T_FH_-DP but with marked differences in the expression of phenotypic, homing, and functional markers. **(B)** Cluster 10 is unique to cT_FH_-DP subsets while cluster 11 **(C)** is observed in T_FH_2 and T_FH_17 subsets. The phenotypic, homing, and functional markers are expressed in each cT_FH_ subset at different levels as shown by the Red-Blue color scheme (Maximum level-red and minimum level-blue) in TD and NoTD.

Cluster 5 was present mostly in cT_FH_-DP (10-40%) (**Figure 10**) and was observed to be at higher frequencies in TD than NoTD participants at all time points but was significantly (p<0.05) higher in cT_FH_-DP after challenge (D7) (**Figure 11M**). These results suggested that cluster 5 may play a role in the development of typhoid disease. Thus, we examined closely the phenotype of cluster 5 in TD and NoTD participants using heatmaps (**Figure 13A**). Interestingly, cT_FH_-DP (CD69+ CD154+ PD1^low^ ICOS+ CD27+) cluster 5 seems to be more activated in TD than in NoTD participants as shown by the phenotype of cT_FH_-DP (CD69^low^ CD154^low^PD-1+ ICOS+ CD27+) (**Figure 13B**). The gut homing marker integrin α4β7 was expressed at higher levels on cT_FH_-DP cluster 5 in TD than in NoTD participants (**Figure 13B**). Remarkably, we observed higher TNF-α,production and CD107a expression in cluster 5 associated with cT_FH_-DP in NoTD which were absent in TD participants (**Figure 13B**).

Cluster 10 is unique to cT_FH_-DP and was observed to be higher in frequencies in NoTD than in TD participants at most time points with a trend (p ≤ 0.1) to show increases at D7 following challenge (**Figure 11O**). Cluster 10 seems to be activated and express similarly all the markers in both TD and NoTD participants except for CD154 (absent from TD), IFNγ (lower in NoTD), GzB (lower in NoTD) and ICOS (lower in NoTD) (**Figure 13B**). Cluster 10 expressed α4β7 and CCR7 in NoTD and TD participants while CCR4 was expressed only in NoTD participants (**Figure 13B**). Interestingly, cytokines (IL-17A, IL-2, MIP-1β, TNF-α, IL-21) and granzyme B were highly expressed on both TD and NoTD participants (**Figure 13B**).

Finally, cluster 11 was present in cT_FH_2 (2-8%) and cT_FH_17 (2-8%) (**Figure 10B**). cT_FH_2 cluster 11 was observed to be higher in frequency in NoTD than in TD participants following Ty21a immunization and after challenge (D7) (**Figure 11G**). These results suggest that cluster 11 may play a role in the prevention of typhoid disease. Thus, we examined closely the phenotype of cluster 11 in TD and NoTD participants using a heatmap (**Figure 13C**). Remarkably, activation markers (CD69, CD154, PD1, ICOS and CD27) in TD and NoTD participants were similarly expressed at high levels on cluster 11 of both cT_FH_2 and cT_FH_17 (**Figure 13C**). Next, we examined homing markers and noted that integrin α4β7, CCR4 and CCR7 were highly expressed on cT_FH_2 and cT_FH_17 in NoTD while in TD participants, the same pattern was present except for lower expression of integrin α4β7 on cT_FH_2 and no expression of CCR4 on cT_FH_17 cluster 11 (**Figure 13C**). Production of most cytokines (IL-17A, MIP-1β, TNF-α, IL-21) and granzyme B was present in cluster 11 in both subsets in NoTD and TD participants (**Figure 13C**). However, we observed that IL-2 expression was high in cT_FH_2 and cT_FH_17 cluster 11 in TD participants but absent from NoTD participants (**Figure 13C**). In addition, IFN-γ was expressed only on cT_FH_2 cluster 11 in NoTD (**Figure 13C**). Altogether, these data suggest that defined clusters appear to be important in the prevention or development of typhoid disease.

### 3.9 Distinct clusters are induced during each phase of the study (e.g., Baseline, Ty21a immunization and wt *S*. Typhi challenge)

**Figure 14:**
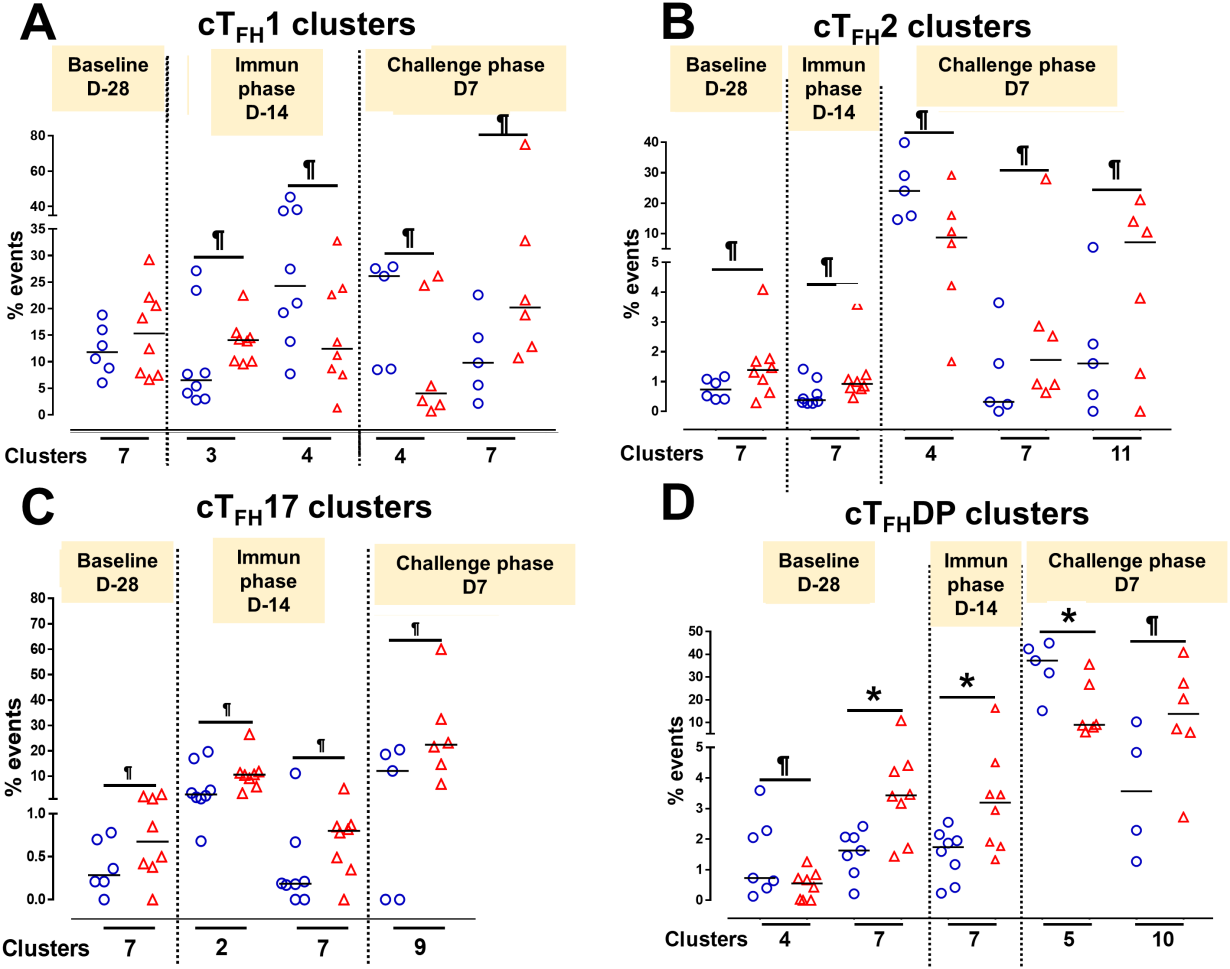
Defined clusters of each cT_FH_ subsets are associated with the development of typhoid disease. Clusters showing significant differences between the participants who developed, or not, typhoid disease were evaluated at each phase of the study, baseline (D-28), immunization (Immun) phase (D-14) and challenge phase (D7) for each cT_FH_ subset: **(A)** cT_FH_1, **(B)** cT_FH_2, **(C)** cT_FH_17 and **(D)** CT_FH_-DP. Differences are shown between the TD and NoTD groups. Symbols are individual participants. *Significant differences (p<0.05). ^¶^ Trends to show significant differences (p ≤ 0.1) between TD and NoTD groups.

To understand the contribution of cT_FH_ subsets in each phase of the study (Baseline, Ty21a immunization and wt *S*. Typhi challenge) to protection, we focused our analysis on some of the clusters that showed significant differences between TD and NoTD participants. For cT_FH_1 subsets, no significant differences in the frequencies of cluster 7 in TD and NoTD were observed at baseline (D-28) (**Figure 14A**). However, following Ty21a vaccination (D-14), cT_FH_1 cluster 3 showed a trend (p ≤ 0.1) to exhibit be present at higher levels in NoTD compared to TD participants, while cT_FH_1 cluster 4 showed a trend (p ≤ 0.1) to exhibit an increase in TD compared to NoTD participants (**Figure 14A**). In the challenge phase, cT_FH_1 cluster 4 continued to show increases in TD compared with NoTD (**Figure 14A**). However, in the challenge phase, cT_FH_1 cluster 7 emerged showing a trend (p ≤ 0.1) to exhibit higher frequencies in NoTD than in TD participants (**Figure 14A**). For cT_FH_2, we observed that cluster 7 had a trend (p ≤ 0.1) to show increases in NoTD compared to TD participants at baseline (D-28) (**Figure 14B**), which remain higher in the immunization phase (**Figure 14B**). However, in the challenge phase, three clusters emerge as important. Clusters 7 and 11 exhibited trends (p ≤ 0.1) to be higher in NoTD than TD participants, while cluster 4 exhibited a trend (p ≤ 0.1) to be higher in TD than in NoTD participants (**Figure 14B**). For cT_FH_17, again we found that cluster 7 exhibited a trend (p ≤ 0.1) to be higher in NoTD than in TD participants at baseline (D-28) (**Figure 14C**). Interestingly, in the immunization phase for cT_FH_17, there were 2 clusters of interest. Clusters 2 and 7 exhibited trends (p ≤ 0.1) to be higher in NoTD than in TD participants (**Figure 14C**). In the challenge phase, another cluster (9) appears to be important. Cluster 9 exhibited a trend (p ≤ 0.1) to be higher in NoTD than TD participants (**Figure 14C**). Finally, for cT_FH_-DP, we observed 2 clusters of importance at baseline, namely clusters 4 and 7 (**Figure 14D**). Cluster 4 exhibited a trend (p ≤ 0.1) to be higher in TD than in NoTD participants, while cluster 7 was significantly (p<0.05) higher in NoTD than in TD participants at baseline (D-28) (**Figure 14D**). In the immunization phase, we found that significantly (p<0.05) higher frequencies of cT_FH_-DP cluster 7 were present in NoTD compared to TD participants (**Figure 14D**). Finally, in the challenge phase, we observed that cT_FH_-DP cluster 5 was significantly (p<0.05) higher in TD than in NoTD participants, while for cT_FH_-DP cluster 10 we observed a trend (p ≤ 0.1) to be higher in NoTD than in TD participants (**Figure 14D**). Taken together, we observed that various clusters and cT_FH_ subsets might be of importance in each phase of the study and might play a role in the development or protection of typhoid disease.

## 4 Correlation of the frequencies of cT_FH_ subsets with *S.* Typhi-specific antibody production in TD and NoTD

**Figure 15:**
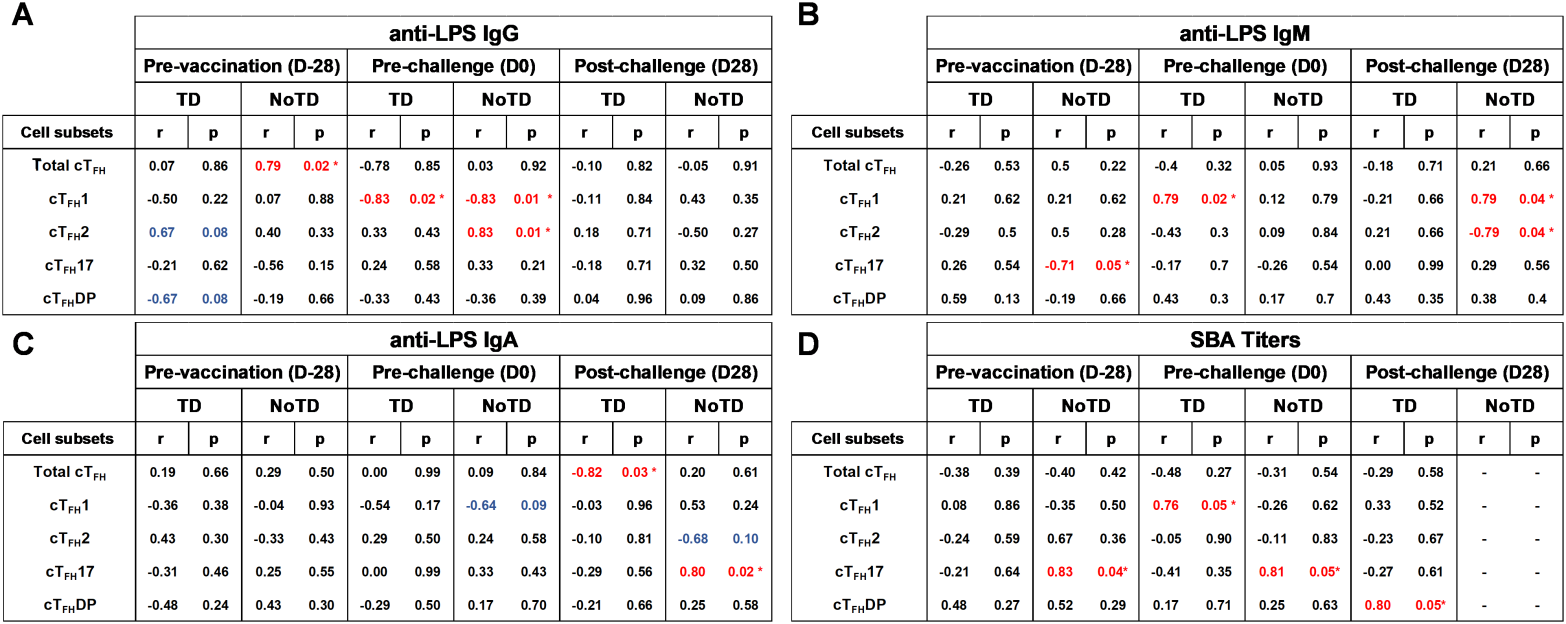
Association between the frequencies of cT_FH_ subsets and *S.* Typhi-specific anti-LPS antibodies production and functional serum bactericidal antibodies (SBA) in TD and NoTD. ELISAs and SBA assays were performed in a set of serum samples obtained at multiple time points (pre-vaccination -D-28-, pre-challenge -D0-, and post-challenge day 28 -D28-) corresponding to the participants (TD *n* = 8, NoTD *n* = 8) in whom the cT_FH_ subsets frequencies and responses were evaluated. Correlation between the frequencies of cT_FH_ subsets (cT_FH_1, cT_FH_2, cT_FH_17, cT_FH_DP) and *S*. Typhi-specific anti-LPS **(A)** IgG, **(B)** IgM and **(C)** IgA were determined using Spearman’s correlation analysis. **(D)** Correlation between bactericidal SBA titers and the frequencies of cT_FH_ subsets (cT_FH_1, cT_FH_2, cT_FH_17, cT_FH_DP) were evaluated. * Strong significant correlation (r>0.7) (p<0.05) (Red).

cT_FH_ subsets play a crucial role in coordinating the immune response, particularly in the generation of antibody responses. cT_FH_ subsets (e.g., cT_FH_ 1, cT_FH_ 2, cT_FH_17) have been found to correlate with the magnitude and quality of antibody responses (8). For example, cT_FH_1 cells have been found to be associated with the production of IgG1 antibodies; cT_FH_2 cells have been linked to the production of IgE and IgG4 antibodies and cT_FH_17 cells have been associated with the production of IgA antibodies (8). Since during primary immune responses IgM is the initial antibody produced before class switching occurs it is reasonable to hypothesize that cT_FH_ subsets might also play a role in providing help to B cells for IgM production. Whether there are correlations between cT_FH_ subsets and IgM production following *S.* Typhi vaccination and infection is unknown. Using Spearman’s correlation analysis, we correlated the frequencies of each cT_FH_ subset to anti-LPS IgG, IgM and IgA levels. Interestingly, we observed a strong significant positive correlation (r=0.79; p<0.05) between total cT_FH_ and anti-LPS IgG in NoTD participants at pre-vaccination (D-28) but not following vaccination or challenge time points (**Figure 15A**). Moreover, we observed a strong significant negative correlation (r=-0.82; p<0.05) between total cT_FH_ and anti-LPS IgA in TD participants at the post-challenge (D28) time point (**Figure 15C**). We next examined the association between cT_FH_1 and anti-LPS antibody production. We found that there was a strong significant negative correlation in TD (r=-0.83; p<0.05) and NoTD (r=-0.83; p<0.05) between cT_FH_1 and anti-LPS IgG following Ty21a vaccination (D0) time point (**Figure 15A**). In addition, there were strong significant positive correlation between cT_FH_1 and anti-LPS IgM in TD (r=0.79; p<0.05) following Ty21a vaccination (D0) and NoTD (r=0.79; p<0.05) following challenge (D28) (**Figure 15B**). However, no correlation between cT_FH_1 and anti-LPS IgA in TD and NoTD was observed at all time points except for a moderate negative trend (r=-0.64; p<0.1) in correlation between cT_FH_1 and anti-LPS IgA in NoTD following Ty21a (D0) (**Figure 15C**). Next, we evaluated the association between cT_FH_2 and anti-LPS antibody production. We found that there was strong significant positive correlation in NoTD (r=0.83; p<0.05) between cT_FH_2 and anti-LPS IgG following Ty21a vaccination (D0) (**Figure 15A**). Moreover, there were strong significant negative correlation between cT_FH_2 and anti-LPS IgM in NoTD (r=-0.79; p<0.05) following challenge (D28) (**Figure 15B**). However, no correlations between cT_FH_2 and anti-LPS IgA in TD and NoTD were observed at any time point (**Figure 15C**). Similarly, we evaluated the association between cT_FH_17 and anti-LPS antibody production. No correlations between cT_FH_17 and anti-LPS IgG in TD and NoTD were observed at any time point (**Figure 15A**). Interestingly, there was a strong significant negative correlation between cT_FH_17 and anti-LPS IgM in NoTD (r=-0.71; p<0.05) before Ty21a vaccination (D-28) (**Figure 15B**). Furthermore, there was a strong significant positive correlation between cT_FH_17 and anti-LPS IgA in NoTD (r=0.80; p<0.05) following challenge (D28) (**Figure 15C**). Additionally, we evaluated the association between cT_FH_DP and anti-LPS antibody production. No significant correlation between cT_FH_DP and anti-LPS IgG, IgM and IgA in TD and NoTD were observed at any time point (**Figures 15A-C**).

We next determined whether cT_FH_ subsets frequencies correlated with anti-H IgG, IgM and IgA production in TD and NoTD at pre-vaccination (D-28) and post vaccination (D0) using Spearman’s correlation analysis. No significant correlations were observed between total cT_FH_ and anti-H IgG, IgM and IgA in TD and NoTD was observed at any time point (**Supplementary Figure S10A-C**). We next examined the association between cT_FH_1 and anti-H antibody production. No significant correlations between cT_FH_1 and anti-H IgG in TD and NoTD were observed at any time point (**Supplementary Figure S10A**). However, there were strong significant positive correlations between cT_FH_1 and anti-H IgM in TD pre-vaccination (D-28) (r=0.74; p<0.05) and following Ty21a vaccination (D0) (r=0.90; p<0.05) (**Supplementary Figure S10B**). In contrast, we observed a strong significant negative correlation between cT_FH_1 and anti-H IgA in NoTD following Ty21a vaccination (r=-0.90; p<0.05) (**Supplementary Figure S10C**). We next evaluated the association between cT_FH_2 and anti-H antibody production. No significant correlations between cT_FH_2 and anti-H IgG in TD and NoTD was observed at any time point (**Supplementary Figure S10A**). However, we observed a strong significant positive correlation between cT_FH_2 and anti-H IgM in NoTD following vaccination (D0) (r=0.79; p<0.05) (**Supplementary Figure S10B**). Similarly, we observed a strong significant positive correlation between cT_FH_2 and anti-H IgA in NoTD following Ty21a vaccination (r=-0.83; p<0.05) (**Supplementary Figure S10C**). We next assessed the association between cT_FH_17 and anti-H antibody production. No significant correlations between cT_FH_17 and anti-H IgG, IgM and IgA in TD and NoTD were observed at any time point (**Supplementary Figure S10A-C**). Finally, we determined the association between cT_FH_DP and anti-H antibody production. No significant correlations between cT_FH_DP and anti-H IgG and IgA in TD and NoTD were observed at any time point (**Supplementary Figure S10A, C**). However, there was a strong significant positive correlation between cT_FH_DP and anti-H IgM in TD pre-vaccination (D-28) (r=0.76; p<0.05) (**Supplementary Figure S10B**). Taken together, each cT_FH_ subset was distinctly associated with *S.* Typhi-specific antibody responses and typhoid disease.

### 4.1 Correlation of cT_FH_ frequencies with serum bactericidal antibodies

Serum bactericidal antibodies are capable of killing bacteria directly through complement activation or opsonization, leading to their clearance from the bloodstream. The correlation between cT_FH_ frequencies and serum bactericidal antibodies, a representation of functional antibody responses, might be an additional indication of the strength of the immune response against *S*. Typhi. For example, higher cT_FH_ subset frequencies may suggest robust B cell activation and antibody production, leading to increased levels of bactericidal antibodies in serum. Conversely, lower cT_FH_ subset frequencies might indicate diminished B cell help and potentially lower levels of serum bactericidal antibodies, which could compromise the ability to control the bacterial infection. Thus, we examined the association between the frequencies of cT_FH_ subsets and *S*. Typhi bactericidal antibodies (SBA) to determine whether they might participate in protection. No significant correlations were observed between total cT_FH_ and SBA in either TD or NoTD at any time point (**Figure 15D**). However, we observed a strong significant positive correlation (r=0.76; p<0.05) between cT_FH_1 and SBA in TD following Ty21a vaccination (**Figure 15D**). No significant correlations were observed between cT_FH_2 and SBA in either TD or NoTD at any time point (**Figure 15D**). In contrast, we found a strong significant positive correlation between cT_FH_17 and SBA in NoTD at pre-vaccination (r=0.83; p<0.05) and following Ty21a vaccination (r=0.81; p<0.05) (**Figure 15D**). Finally, we observed that there was a strong positive correlation between cT_FH_DP and SBA in TD following challenge (r=0.80; p<0.05) (D28). In NoTD participants bactericidal activity a month after challenge (D28) was not measured, as preliminary experiments confirmed complete bactericidal activity consistent with residual ciprofloxacin in the samples (43). This was expected based on its pharmacokinetic profile (D28 coincided with the last day of antibiotic treatment for this cohort) (59). In sum, cT_FH_ subsets may be used as surrogate’s markers to indicate either susceptibility or protection against typhoid fever.

## 5 Discussion

*S*. Typhi, the causative agent of typhoid fever, is a human host restricted bacterium that causes major health problems worldwide, especially in limited resource settings. Numerous studies have demonstrated that both humoral and cell mediated immunity, systemically and in the gut mucosa, are elicited following vaccination (e.g., Ty21a, Vi) and/or infection with *S*. Typhi (1, 40, 41, 51, 60–65). CD4^+^ T cell help (T_FH_ in particular) is essential for optimal antibody responses, including the generation of germinal centers (GC) and long-lived plasma cells responses (66, 67). In addition, recent studies have shown that circulating T follicular helper cells (cT_FH_) play an important role in infectious diseases such as HIV and Hepatitis B (68–70). Thus, identifying the role of cT_FH_ in *S*. Typhi infection and vaccination is necessary to better define the immunological correlates of protection and improve future vaccines. Here, we investigated the role of cT_FH_ and its subsets in participants who developed typhoid disease (TD) or not (NoTD) following Ty21a vaccination and *S*. Typhi challenge. We uncovered that the frequencies of total cT_FH_ and subsequently cT_FH_2 and cT_FH_17 subsets are higher in NoTD than in TD participants, particularly following *S*. Typhi challenge (D7). Interestingly, we observed that homing molecules (α4β7, CCR7) and activation markers (CD69, CD154, ICOS, PD-1) were expressed at higher levels on cT_FH_ subsets of TD than in NoTD participants, predominantly after challenge (D7). Importantly, regarding *S*. Typhi-specific cytokine responses, IL-17A was determined for each cT_FH_ subsets (cT_FH_1, cT_FH_2, cT_FH_17) and shown to be produced at higher levels by the cT_FH_17 subset in NoTD than in TD participants at baseline and following Ty21a vaccination and challenge. Unsupervised analysis revealed that there are distinct clusters for each cT_FH_ subsets that are associated with either prevention (e.g., cluster 7) or development of typhoid disease (e.g., cluster 4). These clusters displayed distinct signatures of cytokines, activation and homing markers. Importantly, we observed distinct significant correlations between cT_FH_ subsets (cT_FH_1, cT_FH_2, cT_FH_17) frequencies and levels of anti-*S.* Typhi LPS and H, as well as functional antibodies. Thus, our data reveals important differences in the responses of each cT_FH_ subset between TD and NoTD participants following Ty21a vaccination and *S*. Typhi infection. Taken together, these results contribute major novel information of the role of cT_FH_ following oral Ty21a oral vaccination and *S*. Typhi infection.

CD4^+^ T_FH_ primary function is to provide protection against pathogens by providing critical signals to B cells which allows them to undergo high-affinity selection and development of B memory cells (B_M_) against viral, bacterial, parasite and fungal infections. Due to the ease of access to blood as compared with lymph nodes (LN), circulating follicular T cells (cT_FH_) (memory counterpart of T_FH_) has been pivotal for determining the role of this important T cell subset (T_FH_) in infectious diseases. Several studies have shown that T_FH_ cells play an important role in infectious diseases such as chronic lymphocytic choriomeningitis (LCMV), HIV, hepatitis B, influenza, malaria, SARS-CoV2 and *Streptococcus pyogenes* infection (52, 66, 68–73) and during vaccination (e.g., malaria, hepatitis B) (74, 75). But to our knowledge, no report has focused on the role of cT_FH_ in *S*. Typhi infection, particularly studies involving challenge of participants with wt *S.* Typhi in which cT_FH_ levels, state of activation, cytokine production and homing potential can be associated with the development of typhoid disease. Our observations of the changes in total cT_FH_ frequencies are consistent with observations from other infectious diseases. For example, there were significant increases in total cT_FH_ in participants that did not develop typhoid disease (NoTD) than in TD participants at D7 following *S*. Typhi challenge. Similarly, total cT_FH_ frequencies were higher in acute HIV infected individuals (5-8 weeks post-infection) than in uninfected (68). During SARS-CoV2 infection, a significant increase in cT_FH_-central memory (CM) (CXCR5+ CD45RA- CCR7^hi^ PD-1-) and a significant decrease in cT_FH_-effector memory (EM) (CXCR5+ CD45RA- CCR7^low^ PD-1+) was observed in convalescent compared to healthy participants (73). Similar observations were found in malaria and influenza infections where cT_FH_ was higher in infected individuals than in healthy controls (52, 71). Thus, following infection with pathogens, the frequency of cT_FH_ is altered, a phenomenon that will ultimately reflect the quantity and quality of antibodies and B_M_ cells generated. In our study, following *S*. Typhi infection we observed lower levels of cT_FH_ in TD participants than in NoTD participants at D7 which might be indicative of poor antibody responses in TD that might prevent the control of the development of typhoid disease. This is consistent with data showing that in older people, cT_FH_ cells are lower in frequencies leading to poor antibody responses following influenza vaccination (76).

However, the type of cT_FH_ subsets, rather than measurements involving the entire cT_FH_ cell population, induced following infection and vaccination may hold the key for associating cT_FH_ responses with disease outcome or high-quality antibody responses against defined pathogens. cT_FH_ cells are heterogenous and comprise different subsets related to Th1, Th2, and Th17 cells as reported by Morita et al. (8). Based on the expression of chemokine receptors CXCR3 and CCR6, cT_FH_ can be divided into 4 subsets namely: (i) cT_FH_1 (CXCR3+ CCR6-), (ii) cT_FH_2 (CXCR3- CCR6-), (iii) cT_FH_17 (CXCR3- CCR6+) and (iv) cT_FH_-DP (CXCR3+ CCR6+) (8). The role of these cT_FH_ subsets has been examined and reported in humans following infections with influenza, malaria, COVID (52, 71, 73) and following vaccination with influenza and hepatitis B (67, 71). In our study, we observed a significant difference in the frequencies of cT_FH_ subsets between NoTD and TD groups leading to skewing of the cT_FH_ subsets following both Ty21a vaccination and *S*. Typhi infection. For example, following *S*. Typhi infection, NoTD participants exhibited a skew in cT_FH_ subsets towards cT_FH_2 and cT_FH_17 as compared to TD participants. This is of importance because it has been demonstrated that cT_FH_2 and cT_FH_17 have superior capacity than other cT_FH_ subsets (cT_FH_1 in particular) to facilitate B cell differentiation and maturation (8). Our results showing lower levels of cT_FH_2 and cT_FH_17 in TD participants, particularly following infection, may be an indication of a decrease in functional cT_FH_ subsets leading to lower high-affinity *S*. Typhi antibody responses and perhaps *S*. Typhi-specific B_M_. Of note, a skewing of cT_FH_ subsets were also observed in HIV infection where both cT_FH_2 and cT_FH_17 cells correlated with the development of broadly neutralizing antibodies to HIV (17). Moreover, following malaria infection (experimental sub-patent malaria) in adult participants, the activation of cT_FH_2 was correlated with antibody development following parasite treatment (52). However, in children infected naturally with malaria, cT_FH_ activation was skewed towards cT_FH_1 resulting in no increase in antibody responses (52). In addition, following hepatitis B vaccination, there was a profound skewing away from cT_FH_2 and cT_FH_17 towards cT_FH_1 in low vaccine responders. This skewing correlated with IL-21 production and protective antibody titers (75). Furthermore, following human papillomavirus vaccination, PD-1+ ICOS+ cT_FH_2 cells were induced (77) while following rVSV-ZEBOV Ebola vaccination, the cT_FH_17 cell subset was induced (78). In contrast, following influenza vaccination, the cT_FH_1 subset was predominant, likely indicative of suboptimal antibody responses to influenza (71, 79). In COVID infection, there was a significant increase in the frequency of cT_FH_1 cells in convalescence participants compared with healthy participants, which correlated with plasma virus-specific IgG and IgM titers (73). Based on these findings, our data suggest that in NoTD participants, cT_FH_2 and cT_FH_17 are efficiently helping B cells to generate anti-*S*. Typhi-specific antibodies that may help control typhoid disease following a wt *S*. Typhi challenge. This was not found to be the case for TD participants, who exhibited lower levels of cT_FH_2 and cT_FH_17.

Remarkably, while the frequencies of cT_FH_ were higher in NoTD than in TD participants, we observed that the homing (integrin α4β7 and CCR7) and activation (CD69, CD154, PD-1 and ICOS) markers of cT_FH_ subsets were higher in TD than NoTD participants, particularly at D7 after *S*. Typhi challenge. The expression of the gut homing molecule integrin α4β7 allows for the selective cell homing to the gut which is the site of entry for *S*. Typhi (80, 81). CC-chemokine receptor 7 (CCR7) and its ligands (CCL19 and CCL21) play a key role in lymphocyte homing to the lymph nodes and intestinal Peyer’s patches (82). Both homing markers were found to be higher on all three cT_FH_ subsets in TD than in NoTD participants. These data suggest that in NoTD participants, cT_FH_ subsets may have already migrated to the gut and lymph nodes or other extraintestinal sites where they primed cognate B cells to mature and differentiate to produce *S*. Typhi-specific antibodies following Ty21a immunization before the challenge with wt *S.* Typhi. In contrast, in TD participants, cT_FH_ subsets expressing homing markers (e.g., integrin α4β7) are still present at high levels in blood at D0 (day of challenge) and D7 (7 days after challenge). These data suggest that in TD, the capability of cT_FH_ to home to the intestine and lymph nodes to interact with B cells might be somewhat affected. If present, this alteration of homing capabilities of cT_FH_ in TD might result in a lower ability to halt or delay the development of typhoid disease. Similarly, following *S*. Typhi challenge, activation (as measured by expression of ICOS, PD-1, CD154 and CD69) of the three cT_FH_ subsets were higher in TD than NoTD participants particularly after *S*. Typhi challenge. These data suggest that cT_FH_ in TD participants are activated following the challenge while cT_FH_ in NoTD exhibited lower activation levels because they might have been activated following vaccination and/or earlier during the infection and most of the cells have already homed to the gut and lymph nodes. Similar observations of cT_FH_ expressing integrin β7 has been found in participants vaccinated with an oral inactivated enterotoxigenic *Escherichia coli* vaccine. The authors reported that there was an upregulation of integrin β7 in activated ICOS+ cT_FH_ and circulating plasmablasts following oral vaccination (83). This allowed cT_FH_ cells to migrate to GC and enter B cells follicles in the Peyer’s patches. Similarly, cT_FH_ subsets expressing CCR7 and CD62L have the capacity to migrate to the secondary lymphoid organs (e.g., lymph nodes). Thus, it appears that expression of homing markers on cT_FH_ subsets play an important role in *S*. Typhi infection, and the timing of the upregulation of homing molecules (integrin α4β7 and CCR7) and activation markers (ICOS, PD-1, CD154 and CD69) may be important indicators of whether typhoid disease will develop or not.

Antibodies are important effector molecules against pathogens. Cytokine-skewed cT_FH_ can influence the magnitude and quality of these antibody responses and, therefore, it is important to address the role of cT_FH_ subsets producing antigen-specific cytokines in infectious diseases. Here, we determined the production of net *S*. Typhi-specific cytokines for each subset namely: (i) IL-21 and IFN-γ by cT_FH_1, (ii) IL-21 and IL-2 by cT_FH_2 and (iii) IL-21 and IL-17A by cT_FH_17. IL-21 produced by cT_FH_ plays multiple crucial roles during B cells help including plasma cell differentiation, hypermutation, class switching, induction and maintenance of GCs and development of plasma cells and B_M_ (56, 84). Interestingly, in our study, we observed that the production of net *S*. Typhi-specific IL-21 by cT_FH_1, cT_FH_ 2 and cT_FH_ 17 were higher in NoTD than in TD participants at all time points except following *S*. Typhi challenge at D7. These data suggests that in NoTD, cT_FH_ subsets are more efficient in providing help for B cell functions than in TD participants to produce high affinity antibodies and B_M_. In addition, cT_FH_ subsets specific *S*. Typhi-responsive cytokines for cT_FH_1 (IFN-γ, which increases T-bet expression on GC B cells leading to switching to IgG2a and IgG2c (85)) and cT_FH_17 (IL-17A, which enhances cognate T-B interactions and induces switching to IgG2a and IgG3 (86)) were produced at higher levels in NoTD than in TD participants at all time points except at D7 following *S*. Typhi challenge. These results suggest that cytokine skewed cT_FH_ subsets were more abundant in NoTD participants than in TD participants. This may lead to optimal class switching and high affinity antibodies against *S*. Typhi in NoTD participants. The role of cytokine-skewed cT_FH_ has been examined in other infectious disease settings. For example, it has been reported that cT_FH_ and T_FH_-mediated GC responses were compromised by Type I IFN signaling during malaria, in both the *P. yoelli* and *P. chabaudi* models (87, 88). It appears that IFN-γ may limit the expression of ICOS on cT_FH_, leading to decreased GC B cell responses, parasite specific antibodies and control of parasite growth (87). However, IFN-γ blockage alone did not restore fully the T_FH_ responses in malaria. Thus, depending on the cytokine skewing of cT_FH_ and on the infection model, cT_FH_ functions can be limited or enhanced resulting in either poor or efficient antibody responses. In our model, the data herein indicate that in NoTD participants there are enhanced cT_FH_ frequencies and functions, while TD participants have limited functions which may lead to typhoid disease. Thus, our findings suggest that cytokine-skewed cT_FH_ subsets may differentially shape the quality of human humoral *S*. Typhi immunity.

In our studies herein, we have established that cT_FH_ subsets play an important role in the development or prevention of typhoid disease by applying standard supervised flow cytometry gating strategies. However, to study these responses in greater depth and to confirm the findings obtained by manual gating, we subsequently used an unsupervised/unbiased analytical approach to further dissect the high dimensional data set generated by mass cytometry and draw insights into the functions of the various cT_FH_ subsets. This approach led us to several key findings. First, we observed that cT_FH_ existed in multiple clusters (11 in total) as analyzed by UMAP in conjunction with PhenoGraph. Interestingly, we noted that the clusters containing cT_FH_ subsets were quite distinct from the clusters that contained cT_FH_2 and cT_FH_17 subsets. More importantly, we found that some of the clusters (e.g., cT_FH_1 and cT_FH_2 cluster 4, cT_FH_17 cluster 8 and cT_FH_-DP cluster 5) are more abundant in TD participants than in NoTD at all time points. In contrast, other clusters (e.g., cT_FH_1 and cT_FH_2 cluster 7, T_FH_2 cluster 11, T_FH_17 cluster 9, T_FH_-DP clusters 7 and 10) were higher in NoTD than in TD participants. These results suggest that there are cT_FH_ clusters associated with the development (TD-associated clusters) or prevention (NoTD-associated clusters) of typhoid disease. The functions of these clusters are intriguing.

Several studies have defined distinct activation subsets of cT_FH_ based on the expression of ICOS, PD-1 and CCR7 (17, 18, 89). It has been shown that ICOS+ PD-1+ cT_FH_ expressed Ki-67, a marker of active cell cycle which suggest that this subset is activated, whereas both ICOS- PD-1+ and ICOS- PD-1- do not expressed Ki67 and hence are described as in a quiescent state (17, 89). Furthermore, it has been found that CCR7 is expressed differentially on these three populations with a negative correlation with PD-1 expression (17, 89). CCR7 expression is lowest on ICOS+ PD-1+ cT_FH_ and highest on ICOS- PD-1-cT_FH_ which may reflect their distinct propensity to enter B cells follicles (90). For example, it has been shown that cT_FH_2 and cT_FH_17 cells expressing ICOS- PD-1+ CCR7^int^ induced memory B cells to become Ig-producing cells (17, 18). However, the ICOS- PD-1- CCDR7^hi^ counterparts did not induce memory B cells. In addition, ICOS- PD-1- CCR7^int^ cT_FH_2 and cT_FH_17 exhibited gene expression profiles resembling those of tonsillar T_FH_ cells (17, 18). Taken together, these data demonstrate that even quiescent cT_FH_2 and T_FH_17 display T_FH_ functions and gene profiles closer to defined tonsillar T_FH_ lineages. In our study, we observed cT_FH_ clusters that have either activated or quiescent profiles based on these three markers (ICOS, PD-1 and CCR7) but, importantly, we were able to assess simultaneously the functional properties of these clusters. For example, the phenotype of cluster 4 in NoTD is composed of cT_FH_1 (ICOS+ PD-1+ CCR7^hi^), cT_FH_2 (ICOS+ PD-1- CCR7^lo/int^), cT_FH_17 (ICOS+ PD-1- CCR7^hi^), cT_FH_-DP (ICOS+ PD-1- CCR7^lo/int^). Based on the classification resulting from the expression of these three markers, cluster 4 in NoTD seems to be in a quiescent state for all cT_FH_ subsets. Similarly, the phenotype of cluster 4 in TD participants is composed of cT_FH_1 (ICOS+ PD-1+ CCR7^hi^), cT_FH_2 (ICOS+ PD-1- CCR7^hi^), cT_FH_17 (ICOS+ PD-1+ CCR7^hi^), and cT_FH_-DP (ICOS+ PD-1+ CCR7^lo^). This suggest that cluster 4 in TD appears to be largely in a quiescent state for all cT_FH_ subsets except for cT_FH_-DP. Moreover, we observed the production of various combinations of cytokines/chemokines in cluster 4 based on the cT_FH_ subset and whether they developed, or not, typhoid disease. We observed multiple cytokine production profiles: cT_FH1_ (NoTD: IL-17A+ MIP1β+ TNFα+ IL-21+ and TD: TNFα+ IL-21+); cT_FH_2 (NoTD: IL-2^int^, MIP1β+, TNFα+, IL-21+ and TD: none): cT_FH_17 (NoTD: IL-17A^low^, MIP1β^int^ and TD: IL-2^int^, MIP1β+, TNFα+, IL-21^int^) and cT_FH_-DP (NoTD: MIP1β+ TNFα+ and TD: IL-17A+ MIP1β+ TNFα+). Additionally, the homing marker α4β7 on cluster 4 was not expressed on cT_FH_2 and cT_FH_17 in NoTD. This suggests that key subsets of cluster 4 cT_FH_ in NoTD are not homing to the gut. In contrast, cluster 4 in TD participants expressed high levels of integrin α4β7 on all the cT_FH_ subsets suggesting that they are capable of homing to the intestine. Taken together, these data indicates that in TD participants, cluster 4 is abundant and homing to the site of infection (gut) but they are not highly activated and consequently unlikely to provide appropriate help to B cells. This may contribute to typhoid disease.

On the other hand, cluster 7, which is associated with the prevention of typhoid disease is expressed highly in NoTD participants at baseline, following Ty21a vaccination and *S.* Typhi challenge, appears to be activated (CD69+ CD154+ ICOS+), express homing molecules (e.g., integrin α4β7, CCR4, CD62L, CCR7) and produce high levels of cytokines cytokines/chemokines (e.g., IL-17, IL-21, TNF-α, MIP-1β), as well as granzyme B and express high levels of CD107, particularly in NoTD participants. These data suggest that cluster 7, which is abundant in NoTD participants, is efficiently activated and therefore likely to induce the maturation and differentiation of B cells, resulting in optimal antibody production. This is consistent with other studies, such as influenza vaccination (71, 79) which has been shown to elicit an increase of ICOS+ PD-1+ CCR7^lo^ cT_FH_1 which positively correlated with the generation of protective antibody responses (71). However, *in vitro* assessment of ICOS+ PD-1+ CCR7^lo^ showed that they have limited helper capacity to induce naïve B cells to produce antibodies (71). This observation suggests that cT_FH_1 has the capacity to contribute to antibody responses, but only when they become ICOS+ PD-1+ CCR7^lo^ activated cells. Thus, cT_FH_1 function in humoral immunity is context-dependent and pathogen-specific because cT_FH_1 cells can be beneficial in viral infections, but detrimental in other infections. In our study, cluster 7 has a unique effector signature in all cT_FH_ subsets which have been correlated with efficient B cell help whereas cluster 4 effector signature seems to be less efficient in activating B cells. Finally, we have found that cluster 7 in all cT_FH_ subsets is higher in NoTD at baseline, following Ty21a vaccination and following *S*. Typhi challenge. While some clusters (2–4) are induced by Ty21a vaccination, other clusters (4, 9–11) are elicited following *S*. Typhi challenge, some in TD and some in NoTD.

From our observations in this study, defined cT_FH_ subsets and clusters are associated with protection against typhoid disease and hence contribute another important measure to the known correlates of protection to *S.* Typhi infection. Interestingly, we note that baseline frequencies of cT_FH_ subsets (cT_FH_2 and cT_FH_17) correlate with protection to typhoid disease. This adds further information regarding key immune responses that are associated with protection in addition to those that we reported regarding *S.* Typhi specific CD8^+^ T_EM_ multifunctional responses where baseline levels were associated with protection against typhoid disease and delayed disease onset (38). In contrast, baseline up-regulation of the gut homing molecule integrin α4β7 in regulatory T cells was associated with the development of TD (91). Of note, baseline activation status, homing potential and frequencies of monocytes, dendritic cells and B cells, as well as mucosal associated invariant T cells, a subset of CD8+ T cells, do not appear to correlate with clinical outcome for typhoid disease following challenge (2, 92–94). In sum, we conclude that cT_FH_ subsets/clusters contribute to the identification of correlates of protection against typhoid disease.

Given the well-established role of cT_FH_ in antibody production (8, 79, 95), we deemed important to evaluate whether there were associations between cT_FH_ subsets and specific antibody production to *S.* Typhi antigens, both by ELISA and functional antibody responses (e.g., SBA). This is the first report correlating the frequencies of defined cT_FH_ subsets with *S*. Typhi specific IgG, IgM, IgA levels measured by ELISA, as well as bactericidal SBA responses. We observed that defined cT_FH_ subsets correlate with the production of specific antibody isotypes. For example, in TD participants, cT_FH_1 frequencies were significantly positively correlated to anti-LPS IgM but significantly negatively correlated to anti-LPS IgG while showing no correlation to IgA following Ty21a vaccination. In contrast, in NoTD participants, cT_FH_1 frequencies were negatively correlated to *S*. Typhi specific anti-LPS IgG but no correlations were found with anti-LPS IgM or IgA following Ty21a vaccination. These data suggest that cT_FH_1 induces IgM B producing cells but do not favor B cells to undergo class-switch recombination (CSR) to switch to IgG or IgA isotypes. This is consistent with other observations that demonstrated that cT_FH_1 have lower efficiency than cT_FH_2 and cT_FH_17 to facilitate B cell differentiation and maturation (8, 79, 96). Interestingly, we also observed that in NoTD, but not in TD participants, that cT_FH_2 frequencies strongly significantly positively correlated with *S*. Typhi specific anti-LPS IgG but not with anti-LPS IgM and IgA following Ty21a vaccination. These data suggest that cT_FH_2 can efficiently induced B cells to class switch and produce *S.* Typhi specific anti-LPS IgG. Remarkably, in NoTD participants, cT_FH_17 strongly significantly positively correlated with *S*. Typhi specific anti-LPS IgA but not with anti-LPS IgG and IgM post-challenge. This is consistent with studies that have reported that cT_FH_17 is efficient in helping B cells to class switch and produce IgA (8). Thus, the data herein confirmed our hypothesis that both cT_FH_2 and cT_FH_17 have superior capacity than other cT_FH_ subsets (cT_FH_1 in particular) in facilitating B cell differentiation and maturation, and therefore participate in protection against typhoid disease.

Furthermore, we correlated the frequencies of cT_FH_ subsets with *S*. Typhi specific anti-H (flagellar antigen) IgG, IgM, IgA levels by ELISA. Of note, we observed that cT_FH_1 frequencies were strongly significantly positively correlated to anti-H IgM at baseline and following Ty21a vaccination in TD participants. No significant correlations were observed for IgG and IgA at baseline or following Ty21a vaccination. However, in NoTD, we observed that cT_FH_1 strongly significantly negatively correlated with anti-H IgA following Ty21a immunization. These data further supports the hypothesis that cT_FH_1 promotes IgM B producing cells but do not stimulate B cells to undergo class-switch recombination (CSR) towards IgG or IgA isotypes. Consistent to what we observed with anti-LPS antibodies, the frequencies of cT_FH_2 in NoTD, but not in TD participants, were strongly significantly positively correlated with anti-H IgM and IgA and IgG (moderately correlated; trend, p=0.06) following Ty21a vaccination. These data suggest that cT_FH_2 can efficiently induced B cells to class switch and produce IgG and/or IgA. Thus, we conclude that it is important to examine not only total cT_FH_ but also the different subsets in order to determine the fine granularity of the responses.

Additionally, in this study, we reported for the first time an association between the frequencies of cT_FH_ subsets and bactericidal activity (SBA) which has been shown to be an established correlate of protection for *Neisseria meningitidis* (97). Interestingly, we observed that cT_FH_1 frequencies strongly significantly positively correlated with SBA titers in TD participants but not in NoTD participants following Ty21a immunization. Thus, these data suggest that bactericidal antibodies associated with cT_FH_1 help were not sufficient to prevent typhoid disease. We also observed that cT_FH_DP frequencies strongly significantly positively correlated with SBA titers in TD participants post-challenge. Thus, these data suggest that bactericidal antibodies associated with increased frequencies of cT_FH_DP help were not sufficient to prevent typhoid disease. In contrast, cT_FH_17 frequencies strongly significantly positively correlated with SBA titers both at baseline and following Ty21a vaccination. These data strongly suggest that cT_FH_17 help B cells to produce bactericidal antibodies that might participate in preventing the development of typhoid disease.

There are few limitations in this study. In particular, the number of participants (e.g., n=8 for TD and n=8 for NoTD) in the study was a relatively small sample size. This may have precluded some of the trends (p ≤ 0.1) to reach statistically significant values of differences in the various cT_FH_ levels, characteristics, and function between TD and NoTD participants. This is, to a large extent, a consequence of the stringent inclusion criteria, extensive training of the investigators involved, and facilities associated with the recruitment of participants in CHIM trials and the numbers of PBMC available. Additionally, the participants of the CHIM study may not be representative of the patients in endemic countries due to many factors including the protective effect of prior exposure to *Salmonella* and other related enteric pathogens but may represent an underestimate of observations in field settings. For example, in a typhoid CHIM study it was observed that the efficacy of a typhoid conjugate vaccine was 52% (98) while in field study, the efficacy of the same vaccine was 81.6% at 1-year (99) and 79% at 2-years (100) in Nepal, 80.7% at 18-24 months (101), and 78.3% at 4-years (102) in Malawi and 85% in Bangladesh at 18 months (103).

Taken together, these data presented herein suggest that distinct clusters may play unique roles in the development or prevention of typhoid disease and that they may be present at baseline or elicited by oral Ty21a vaccination and/or *S*. Typhi challenge. These findings provide novel insights into the complex mechanisms involved in protective immunity regarding the role of cT_FH_ in *S*. Typhi infection. Taken together, the findings included in this manuscript advance our understanding of the contribution of cT_FH_ and its subsets to the immunological correlates of protection from disease in bacterial infections.

## 6 Conclusion

The role of T_FH_ is critical in the development of immune responses to vaccines and infections because of their key function in the generation of GC, antibodies, B_M_ and long live plasma cells. Here, we provide evidence of the association of cT_FH_ responses with the development of typhoid disease following Ty21a vaccination and *S*. Typhi infection using a CHIM model. These results contribute novel insights into our understanding of the role of cT_FH_ subsets as one of the correlates of protection in typhoid disease and in the generation of appropriate antibody responses. Our findings have important implications for vaccine development, suggesting that strategies to target cT_FH_ cells may improve vaccine efficacy.

## Data Availability

The original contributions presented in the study are included in the article/**Supplementary Material**. Further inquiries can be directed to the corresponding authors.

## Supplementary material

Supplementary Figure 1 Cleaning Gating Strategy and PeacoQC analysis of cT_FH_. **(A)** Following the sequential cleaning gating strategy to remove doublets, debris and calibration beads and viability check. The final gated events will be used downstream for analysis. **(B)** Peak Extraction and Cleaning Oriented Quality Control (PeacoQC) plugin (FlowJo) was used to control for the quality of the data to evaluate the sample signal for regions of irregularity.

Supplementary Figure 2 Gating Strategy for cytokines/chemokines and Multifunctionality of CD4^+^CD45RA^-^CXCR5^+^ cT_FH_ *S*. Typhi-specific responses in TD and NoTD volunteers at all time points. **(A)** The expression of cytokines/chemokines (IL-21, CD107a, Granzyme B (GzB), IFNγ, IL-2, IL-17A, MIP1β and TNFα) of activated cT_FH_ (CD4^+^CD45RA^-^CXCR5^+^CD69^+^) were assessed from *S*. Typhi-infected B-EBV and uninfected B-EBV. **(B)** Boolean gating (FlowJo) was used to assess *S*. Typhi-specific responses in total cT_FH_ to determine single producing cells (S) and multifunctional (MF) associated effectors for CD107a, Granzyme, IFNγ, IL-17A and TNFα in TD and NoTD volunteers. Trend (p=0.09) at D7 in IL-17A-associated MF responses between TD and NoTD. ** Significant differences between S and MF-associated IL-17A.

Supplementary Figure 3 cT_FH_2 and cT_FH_17 frequencies were higher in NoTD participants than in TD participants in the immunization and challenge phases. The area under the curve (AUC) for each participant was calculated for the immunization phase (D-28 to D0) and for the challenge phase (D0 to D28) for the frequencies of **(A)** cT_FH_1, **(B)** cT_FH_2 and **(C)** cT_FH_17. Significant differences between TD and NoTD groups are represented by *p<0.05. ^¶^ Trends to show significant differences (p ≤ 0.1) between TD and NoTD groups for each cT_FH_ subset.

Supplementary Figure 4 Impact of Ty21a immunization and wt *S*. Typhi exposure on the frequencies of cT_FH_ subsets. The effect of Ty21a vaccination on the frequency of cT_FH_ subsets was assessed by measuring the frequencies of **(A)** cT_FH_1, **(B)** cT_FH_2 and **(C)** T_FH_17 at D-28 (pre-vaccination) and D-14 (14 days post vaccination) in TD and NoTD participants. The ratio of cT_FH_2+cT_FH_17:cT_FH_1 was determined and compared between the TD and NoTD groups. Similarly, the effect of challenge with wt *S.* Typhi on the frequency of cT_FH_ subsets was assessed by measuring the frequencies of **(D)** cT_FH_1, **(E)** cT_FH_2 and **(F)** cT_FH_17 at D0 (pre-challenge) and D7 (7 days post challenge) in TD and NoTD participants. The ratios of cT_FH_2+cT_FH_17:cT_FH_1 during these time points were determined and compared between the TD and NoTD groups. Each symbol represents and individual participant. Significant differences between TD and NoTD are represented by *P<0.05. ^¶^Trends to show significant differences (p ≤ 0.1) between TD and NoTD groups for each cT_FH_ subset in each phase.

Supplementary Figure 5 cT_FH_ subsets exhibited increased expression of homing and activation markers in TD participants. The area under the curve (AUC) for each participant was calculated for the immunization phase (D-28 to D0) and for the challenge phase (D0 to D28) for CCR7 expression in **(A)** cT_FH_1, **(B**) cT_FH_2 and **(C)** cT_FH_17. Similarly, AUC were calculated for PD1 expression for the immunization phase (D-28 to D0) and for the challenge phase (D0 to D28) in **(D)** cT_FH_1, **(E)** cT_FH_2 and **(F)** cT_FH_17. ^¶^ Trends to show significant differences (p ≤ 0.1) between TD and NoTD groups.

Supplementary Figure 6 cT_FH_ subsets express increased activation markers in TD participants. The area under the curve (AUC) for each participant was calculated for the immunization phase (D-28 to D0) and for the challenge phase (D0 to D28) for CD69 expression in **(A)** cT_FH_1, **(B)** cT_FH_2 and **(C)** cT_FH_17. Similarly, AUC were calculated for CD154 (CD40L) expression for the immunization phase (D-28 to D0) and for the challenge phase (D0 to D28) in **(D)** cT_FH_1, **(E)** cT_FH_2 and **(F)** cT_FH_17. ^¶^ Trends to show significant differences (p ≤ 0.1) between TD and NoTD groups.

Supplementary Figure 7 Homing and activation of cT_FH_ subsets following Ty21a immunization and wt *S*. Typhi Challenge. Ex-vivo expression of **(A)** activation marker CD27 and **(B)** homing marker CD62L were measured and compared between the three cT_FH_ subsets (cT_FH_1, cT_FH_2 and cT_FH_17) in TD (blue lines) and NoTD (red lines) participants following immunization and wt *S*. Typhi challenge.

Supplementary Figure 8
*S*. Typhi-specific responses by cT_FH_ subsets following Ty21a immunization and wt *S*. Typhi Challenge. *S*. Typhi responses were determined by stimulation of cT_FH_ with (i) *S*. Typhi-infected (ST) or (ii) non-infected (NI) autologous EBV-B. The net *S*. Typhi responses were calculated by the difference of ST minus NI in both the immunization and challenge phases in the TD and NoTD groups. Net *S*. Typhi responses (MIP-1β and TNFα) were measured in **(A)** cT_FH_1, **(C)** cT_FH_2 and **(E)** cT_FH_17 in TD and NoTD participants. Similarly, net *S*. Typhi responses (CD107a and granzyme B (GzB)) were measured in **(B)** cT_FH_1, **(D)** cT_FH_2, and **(F)** cT_FH_17 in TD and NoTD participants. Significant differences between TD and NoTD groups are represented by * p<0.05. ^¶^ Trends to show significant differences (p ≤ 0.1) between TD and NoTD groups.

Supplementary Figure 9 Individual histograms showing the expression of individual markers in cluster 4. The expression of the various markers in TD (orange) and NoTD (red) from a representative cluster (cluster 4) from **Figure 8A** is shown in individual histograms.

Supplementary Figure 10 Correlation between the frequencies of cT_FH_ subsets and *S.* Typhi-specific anti-H antibodies production in TD and NoTD. ELISAs were performed in a set of serum samples obtained at two time points (pre-vaccination -D-28-, pre-challenge -D0-) corresponding to the participants (TD *n* = 8, NoTD *n* = 8) in whom the cT_FH_ subsets frequencies and responses were evaluated. Correlation between the frequencies of cT_FH_ subsets (cT_FH_1, cT_FH_2, cT_FH_17, cT_FH_DP) and *S*. Typhi-specific anti-H (Flagellar antigen) **(A)** IgG, **(B)** IgM and **(C)** IgA were determined using Spearman’s correlation analysis. * Strong significant correlation (r>0.7) (p<0.05) (Red).
