# Supplementary figures and images for: Role of circulating T follicular helper subsets following Ty21a immunization and oral challenge with wild type S. Typhi in humans
Source: Front Immunol. 2024 Sep 12;15:1384642. doi: 10.3389/fimmu.2024.1384642 (PMC11424897; doi:10.3389/fimmu.2024.1384642)

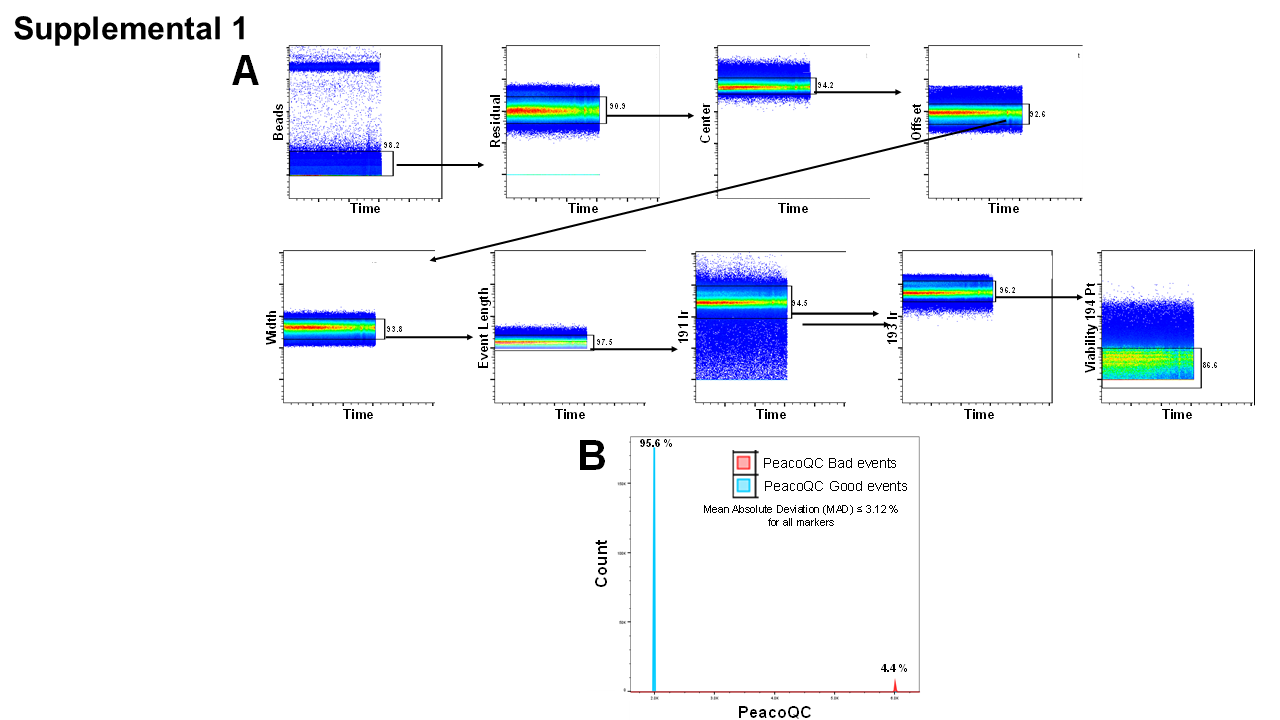

Supplement: Supplementary Figure 1 — Cleaning Gating Strategy and PeacoQC analysis of cTFH. (A) Following the sequential cleaning gating strategy to remove doublets, debris and calibration beads and viability check. The final gated events will be used downstream for analysis. (B) Peak Extraction and Cleaning Oriented Quality Control (PeacoQC) plugin (FlowJo) was used to control for the quality of the data to evaluate the sample signal for regions of irregularity. [file Image6.tif]

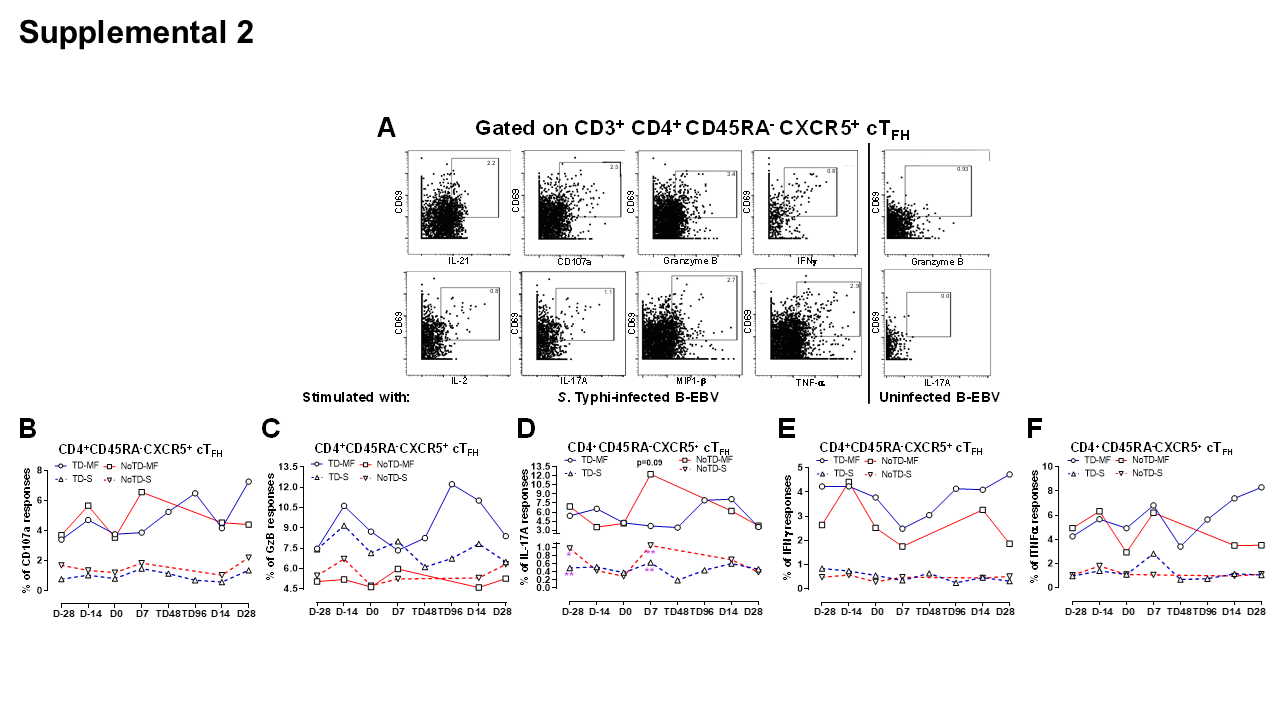

Supplement: Supplementary Figure 2 — Gating Strategy for cytokines/chemokines and Multifunctionality of CD4+CD45RA-CXCR5+ cTFH S. Typhi-specific responses in TD and NoTD volunteers at all time points. (A) The expression of cytokines/chemokines (IL-21, CD107a, Granzyme B (GzB), IFNγ, IL-2, IL-17A, MIP1β and TNFα) of activated cTFH (CD4+CD45RA-CXCR5+CD69+) were assessed from S. Typhi-infected B-EBV and uninfected B-EBV. (B) Boolean gating (FlowJo) was used to assess S. Typhi-specific responses in total cTFH to determine single producing cells (S) and multifunctional (MF) associated effectors for CD107a, Granzyme, IFNγ, IL-17A and TNFα in TD and NoTD volunteers. Trend (p=0.09) at D7 in IL-17A-associated MF responses between TD and NoTD. ** Significant differences between S and MF-associated IL-17A. [file Image7.tif]

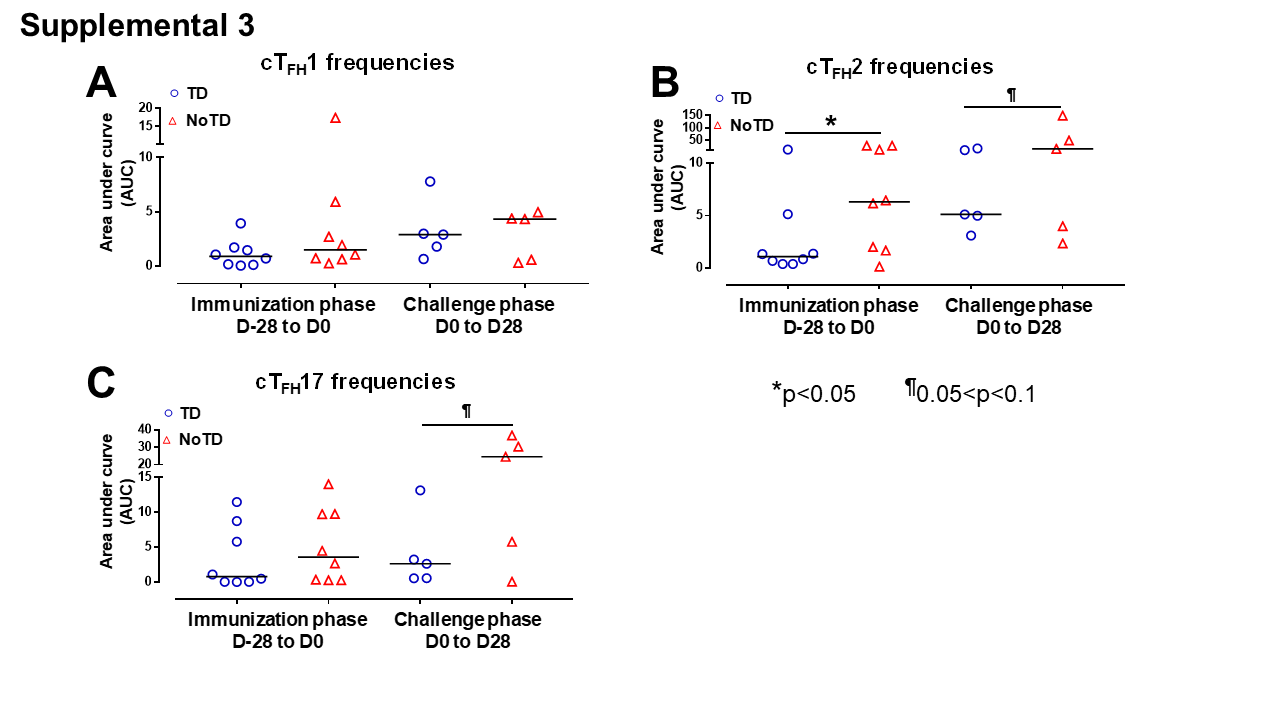

Supplement: Supplementary Figure 3 — cTFH2 and cTFH17 frequencies were higher in NoTD participants than in TD participants in the immunization and challenge phases. The area under the curve (AUC) for each participant was calculated for the immunization phase (D-28 to D0) and for the challenge phase (D0 to D28) for the frequencies of (A) cTFH1, (B) cTFH2 and (C) cTFH17. Significant differences between TD and NoTD groups are represented by *p<0.05. ¶ Trends to show significant differences (p ≤ 0.1) between TD and NoTD groups for each cTFH subset. [file Image8.tif]

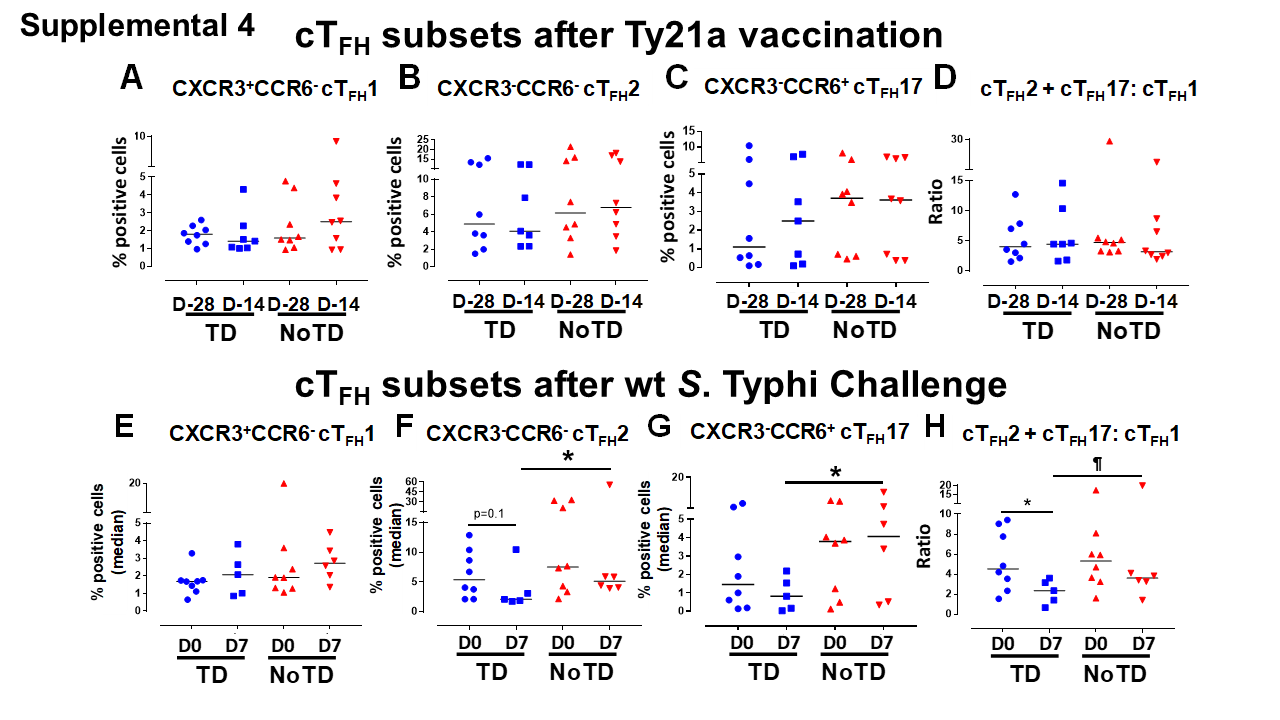

Supplement: Supplementary Figure 4 — Impact of Ty21a immunization and wt S. Typhi exposure on the frequencies of cTFH subsets. The effect of Ty21a vaccination on the frequency of cTFH subsets was assessed by measuring the frequencies of (A) cTFH1, (B) cTFH2 and (C) TFH17 at D-28 (pre-vaccination) and D-14 (14 days post vaccination) in TD and NoTD participants. The ratio of cTFH2+cTFH17:cTFH1 was determined and compared between the TD and NoTD groups. Similarly, the effect of challenge with wt S. Typhi on the frequency of cTFH subsets was assessed by measuring the frequencies of (D) cTFH1, (E) cTFH2 and (F) cTFH17 at D0 (pre-challenge) and D7 (7 days post challenge) in TD and NoTD participants. The ratios of cTFH2+cTFH17:cTFH1 during these time points were determined and compared between the TD and NoTD groups. Each symbol represents and individual participant. Significant differences between TD and NoTD are represented by *P<0.05. ¶Trends to show significant differences (p ≤ 0.1) between TD and NoTD groups for each cTFH subset in each phase. [file Image9.tif]

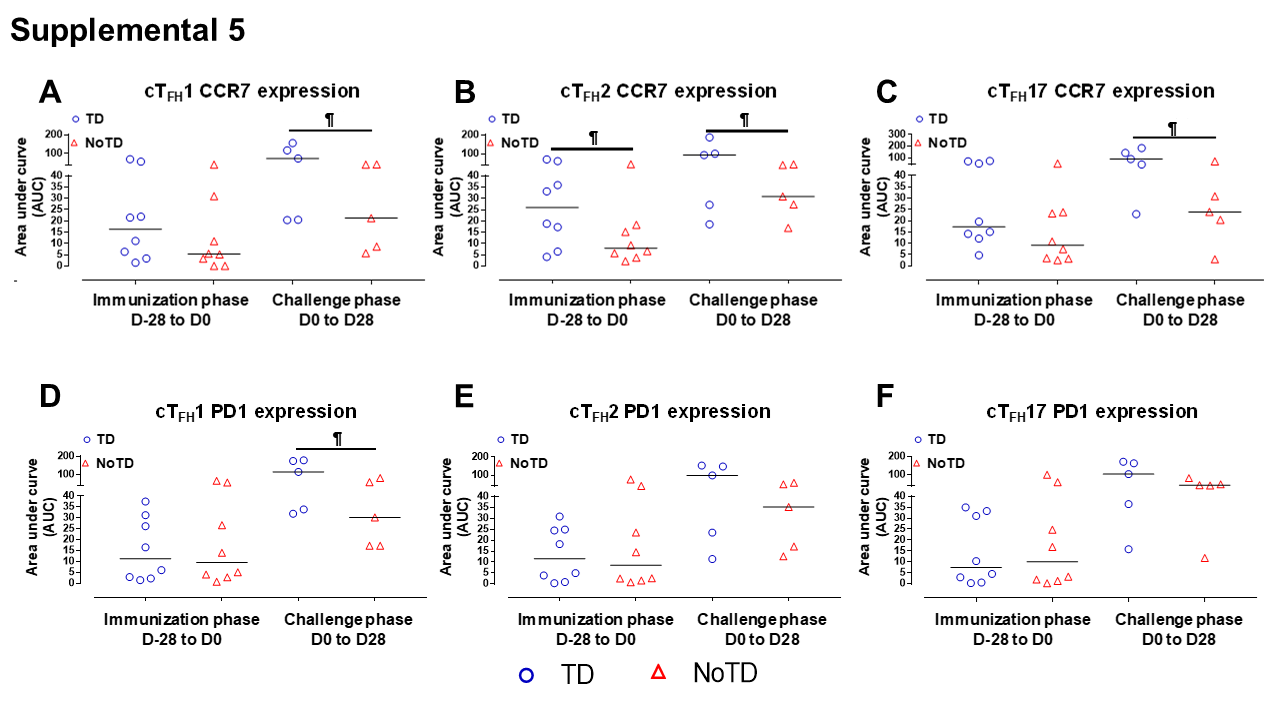

Supplement: Supplementary Figure 5 — cTFH subsets exhibited increased expression of homing and activation markers in TD participants. The area under the curve (AUC) for each participant was calculated for the immunization phase (D-28 to D0) and for the challenge phase (D0 to D28) for CCR7 expression in (A) cTFH1, (B) cTFH2 and (C) cTFH17. Similarly, AUC were calculated for PD1 expression for the immunization phase (D-28 to D0) and for the challenge phase (D0 to D28) in (D) cTFH1, (E) cTFH2 and (F) cTFH17. ¶ Trends to show significant differences (p ≤ 0.1) between TD and NoTD groups. [file Image10.tif]

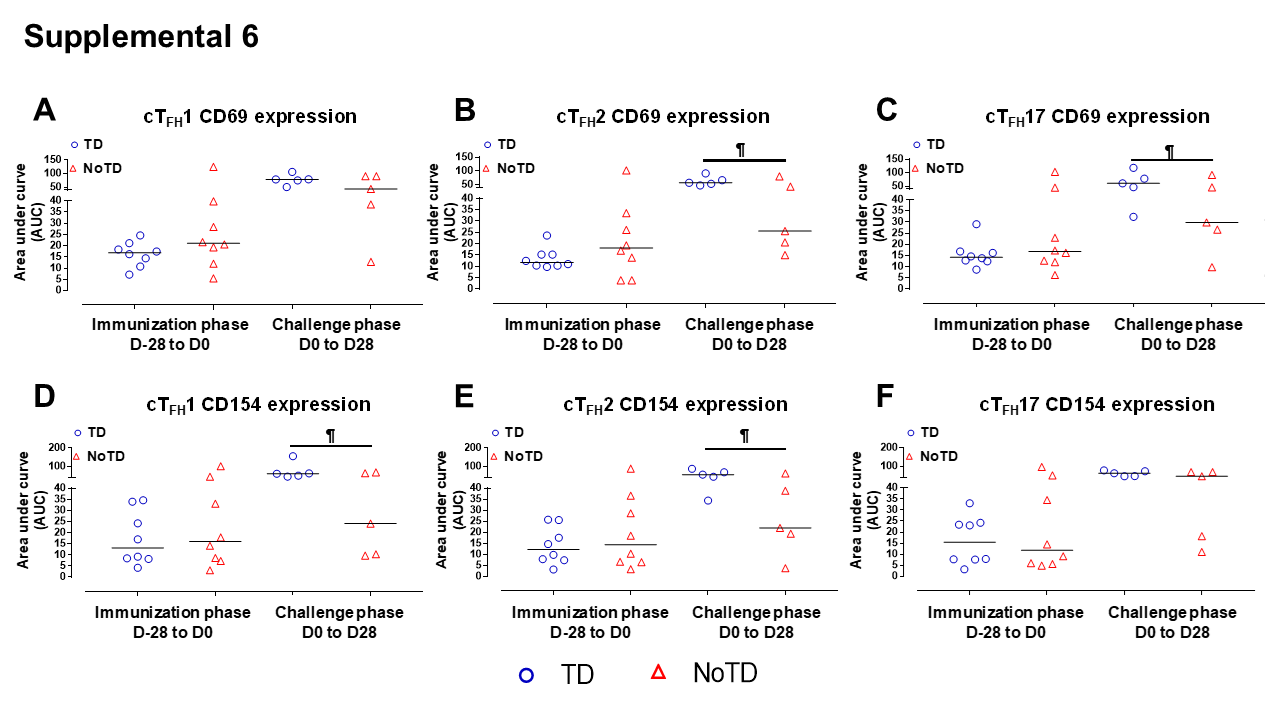

Supplement: Supplementary Figure 6 — cTFH subsets express increased activation markers in TD participants. The area under the curve (AUC) for each participant was calculated for the immunization phase (D-28 to D0) and for the challenge phase (D0 to D28) for CD69 expression in (A) cTFH1, (B) cTFH2 and (C) cTFH17. Similarly, AUC were calculated for CD154 (CD40L) expression for the immunization phase (D-28 to D0) and for the challenge phase (D0 to D28) in (D) cTFH1, (E) cTFH2 and (F) cTFH17. ¶ Trends to show significant differences (p ≤ 0.1) between TD and NoTD groups. [file Image11.tif]

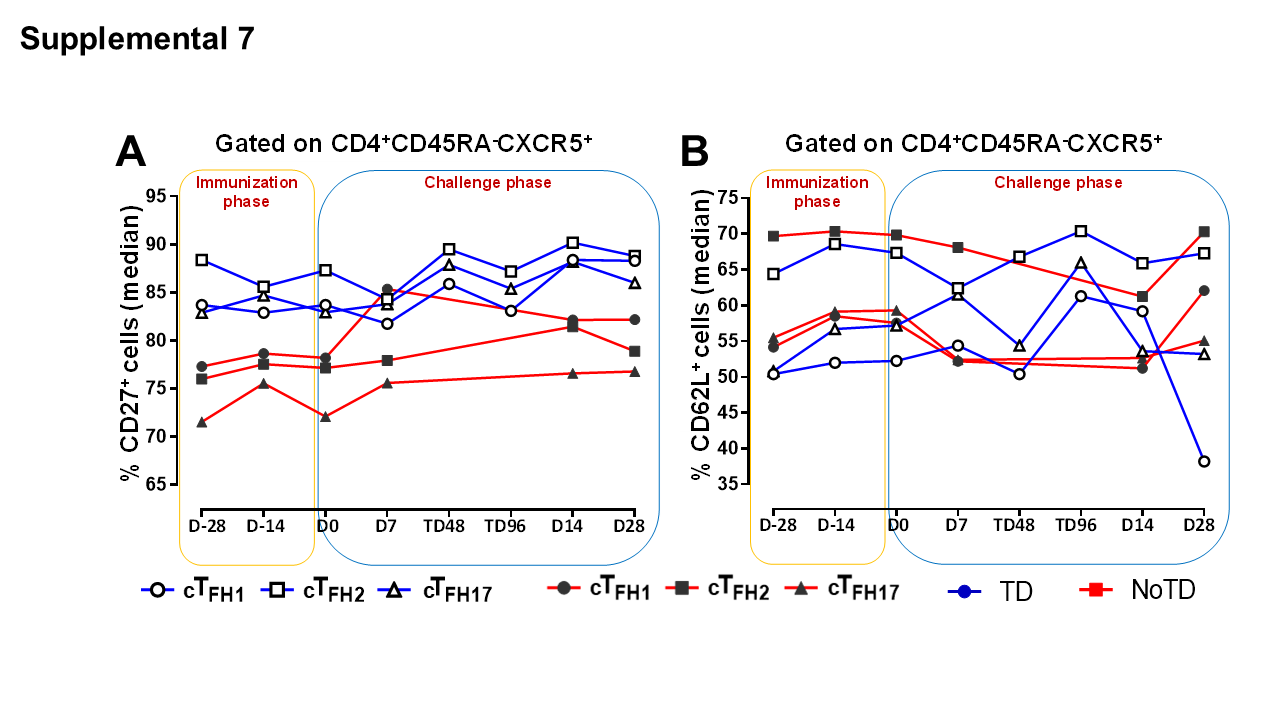

Supplement: Supplementary Figure 7 — Homing and activation of cTFH subsets following Ty21a immunization and wt S. Typhi Challenge. Ex-vivo expression of (A) activation marker CD27 and (B) homing marker CD62L were measured and compared between the three cTFH subsets (cTFH1, cTFH2 and cTFH17) in TD (blue lines) and NoTD (red lines) participants following immunization and wt S. Typhi challenge. [file Image12.tif]

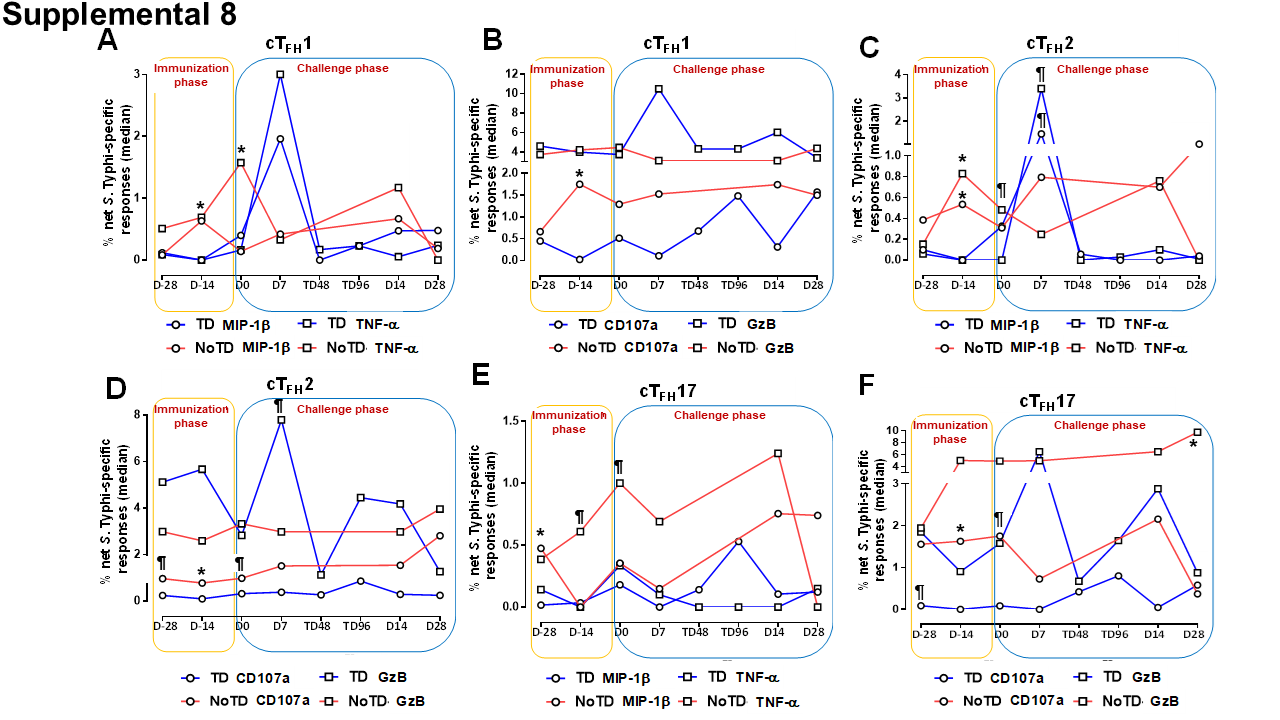

Supplement: Supplementary Figure 8 — S. Typhi-specific responses by cTFH subsets following Ty21a immunization and wt S. Typhi Challenge. S. Typhi responses were determined by stimulation of cTFH with (i) S. Typhi-infected (ST) or (ii) non-infected (NI) autologous EBV-B. The net S. Typhi responses were calculated by the difference of ST minus NI in both the immunization and challenge phases in the TD and NoTD groups. Net S. Typhi responses (MIP-1β and TNFα) were measured in (A) cTFH1, (C) cTFH2 and (E) cTFH17 in TD and NoTD participants. Similarly, net S. Typhi responses (CD107a and granzyme B (GzB)) were measured in (B) cTFH1, (D) cTFH2, and (F) cTFH17 in TD and NoTD participants. Significant differences between TD and NoTD groups are represented by * p<0.05. ¶ Trends to show significant differences (p ≤ 0.1) between TD and NoTD groups. [file Image13.tif]

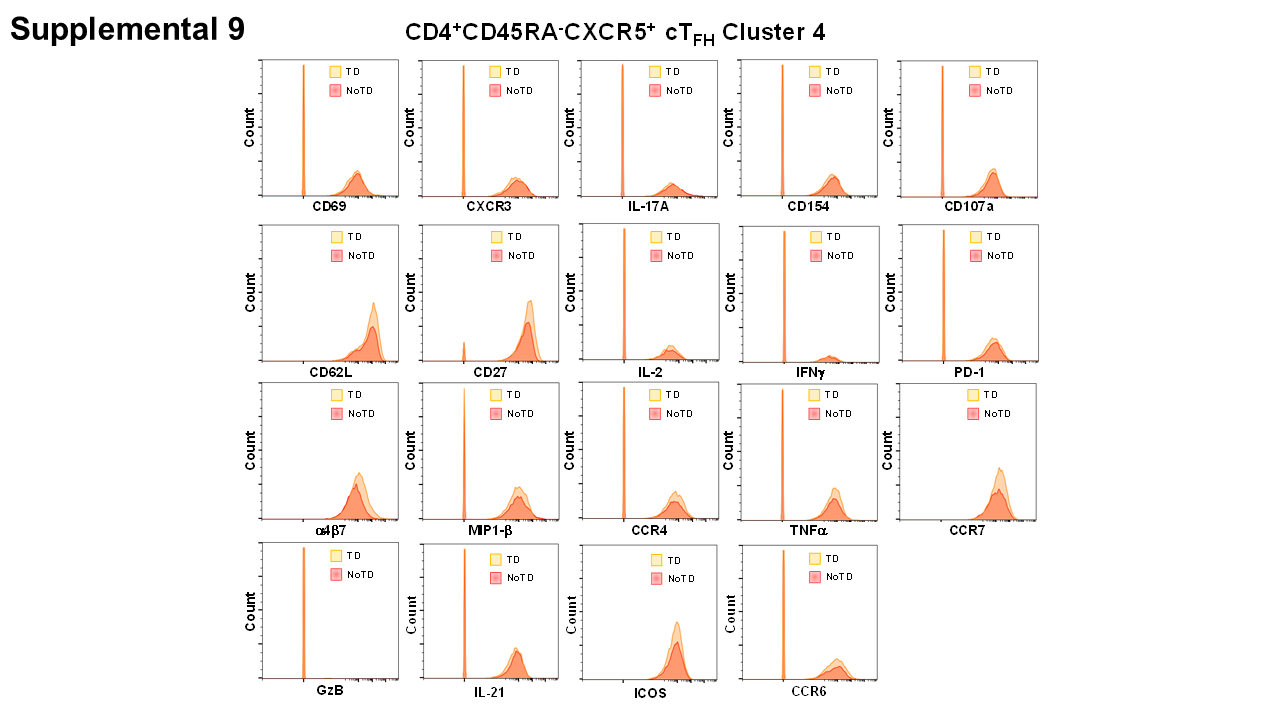

Supplement: Supplementary Figure 9 — Individual histograms showing the expression of individual markers in cluster 4. The expression of the various markers in TD (orange) and NoTD (red) from a representative cluster (cluster 4) from Figure 8A is shown in individual histograms. [file Image14.tif]

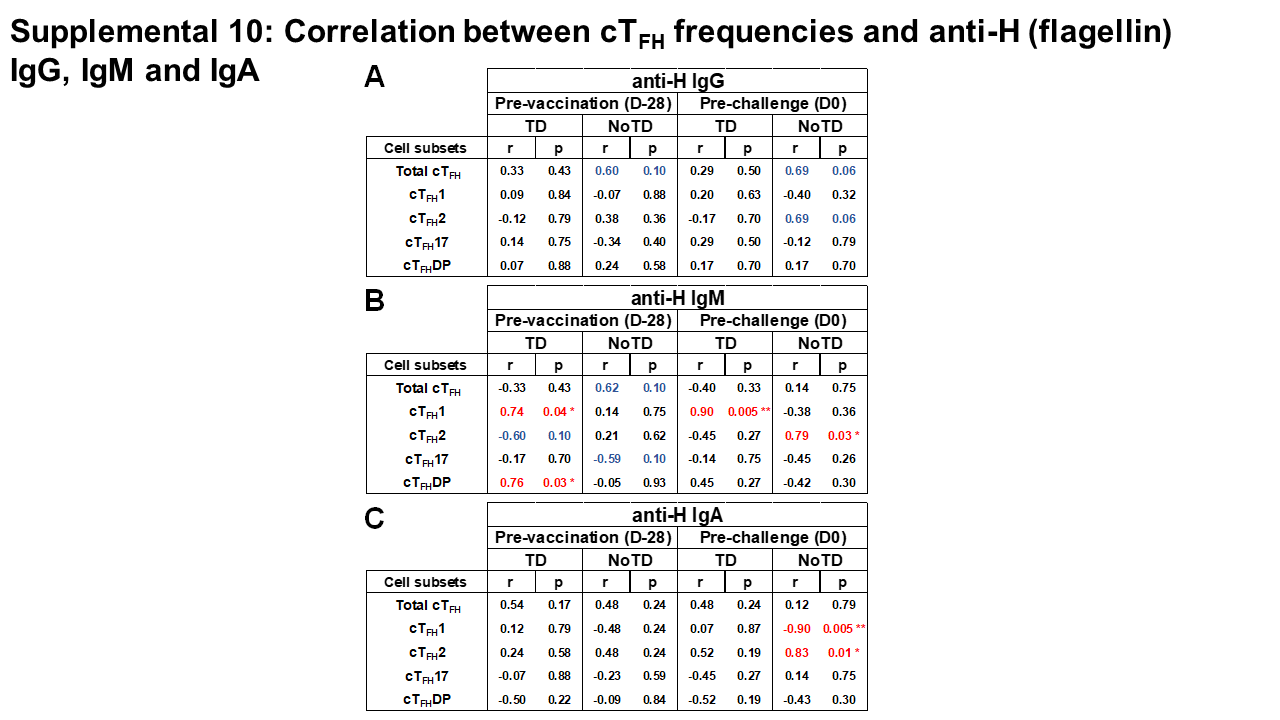

Supplement: Supplementary Figure 10 — Correlation between the frequencies of cTFH subsets and S. Typhi-specific anti-H antibodies production in TD and NoTD. ELISAs were performed in a set of serum samples obtained at two time points (pre-vaccination -D-28-, pre-challenge -D0-) corresponding to the participants (TD n = 8, NoTD n = 8) in whom the cTFH subsets frequencies and responses were evaluated. Correlation between the frequencies of cTFH subsets (cTFH1, cTFH2, cTFH17, cTFHDP) and S. Typhi-specific anti-H (Flagellar antigen) (A) IgG, (B) IgM and (C) IgA were determined using Spearman’s correlation analysis. * Strong significant correlation (r>0.7) (p<0.05) (Red). [file Image15.tif]

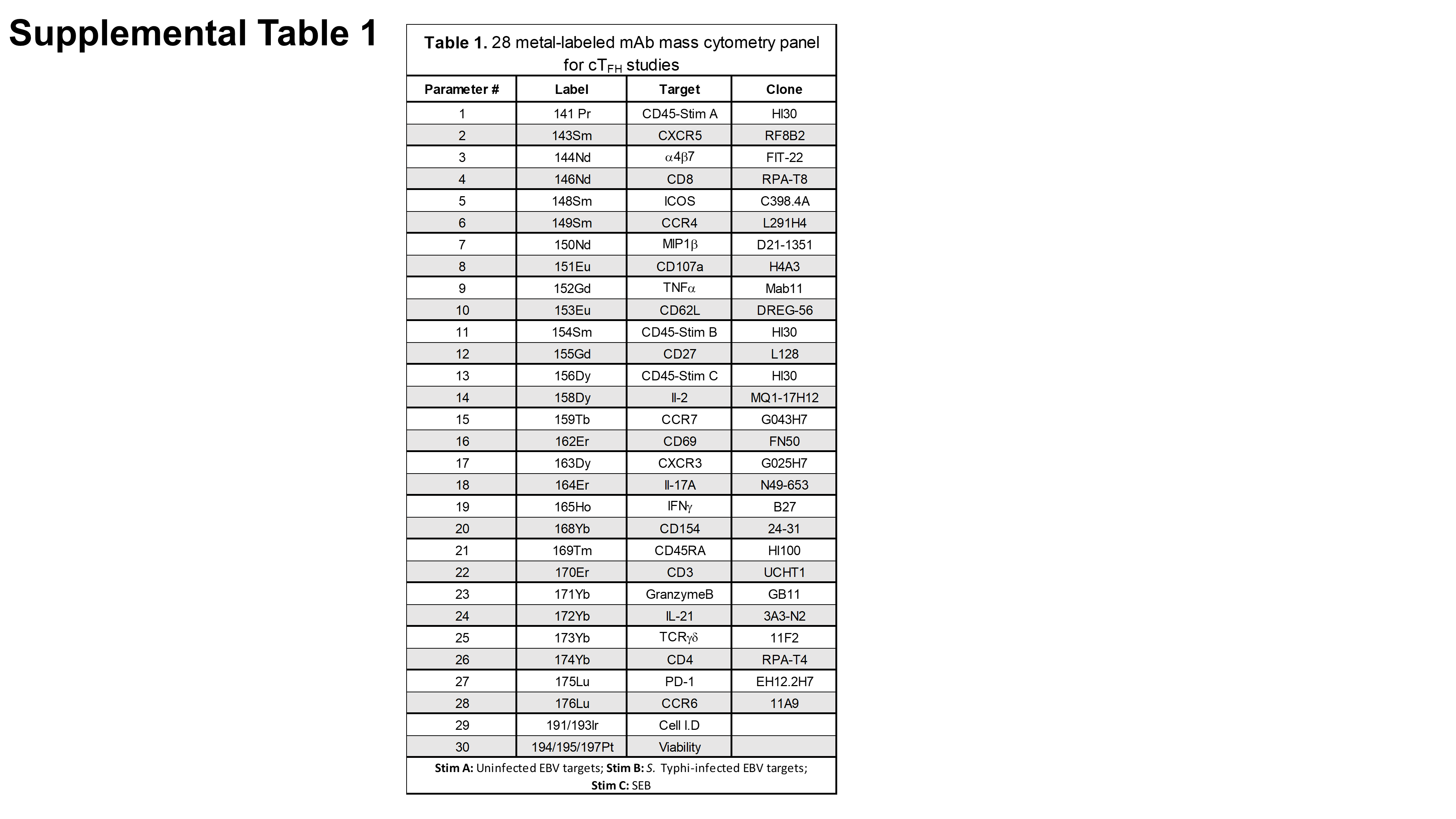

Supplement: Supplementary file 11 [file Image1.tif]

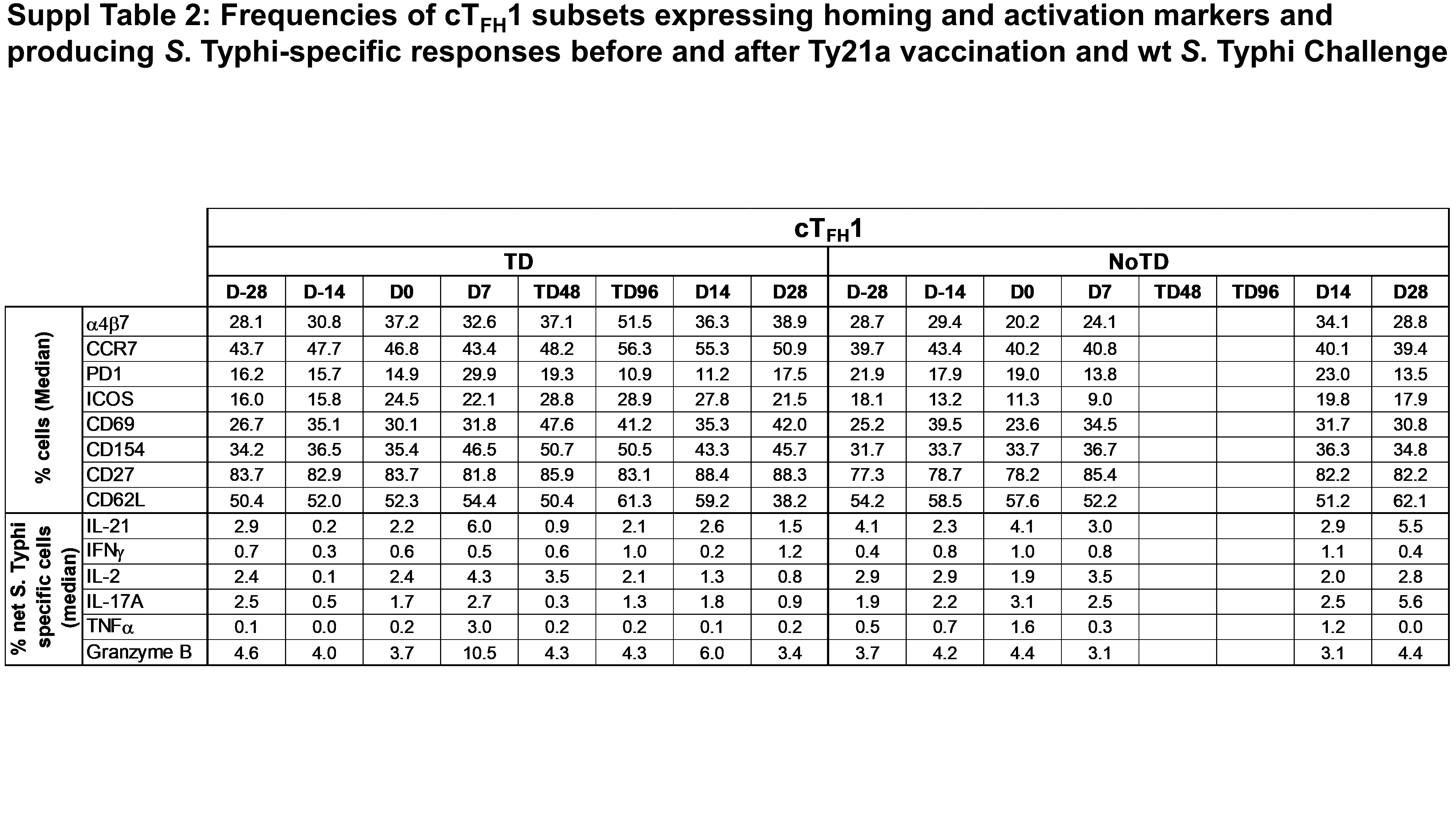

Supplement: Supplementary file 12 [file Image2.tif]

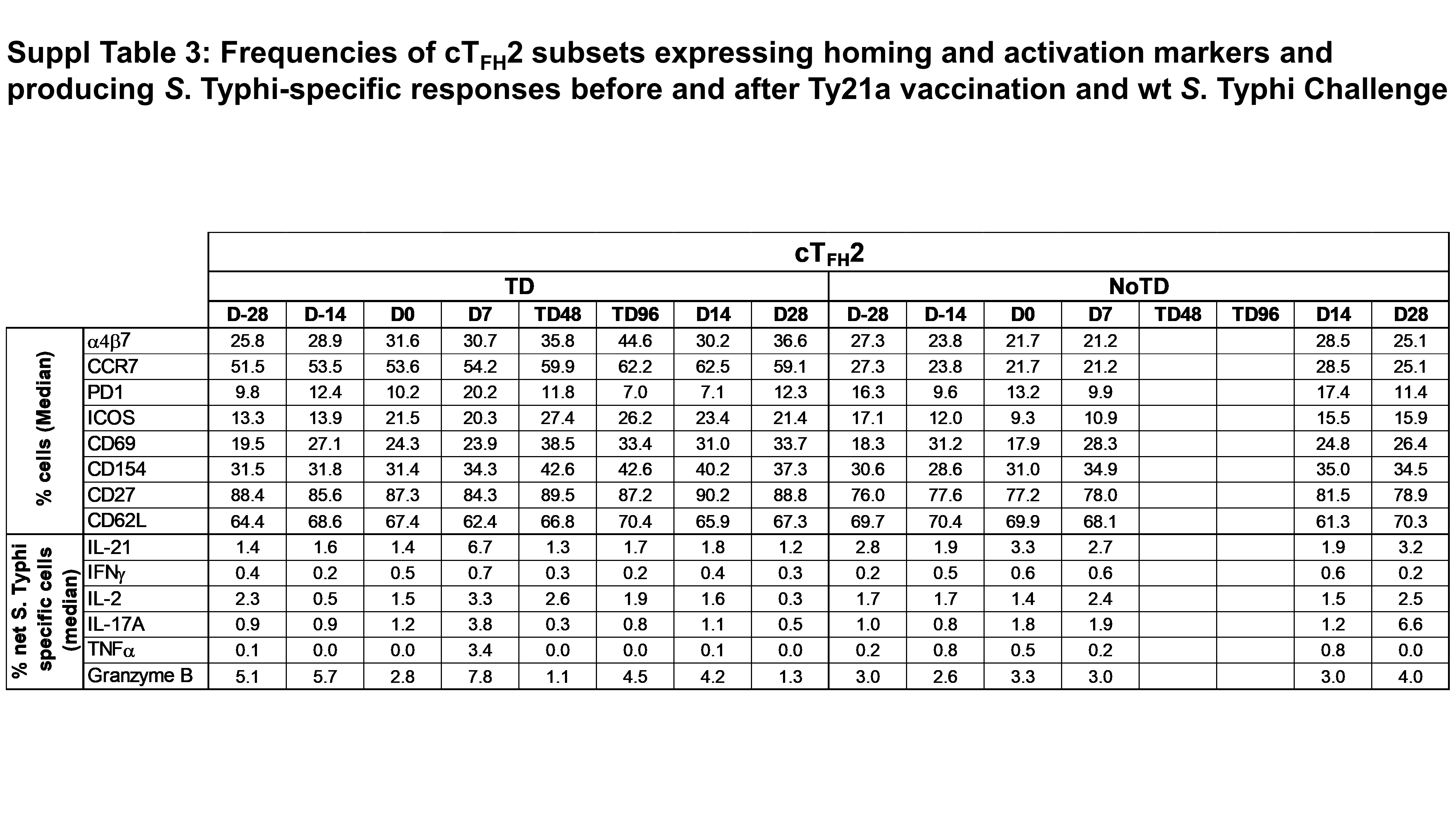

Supplement: Supplementary file 13 [file Image3.tif]

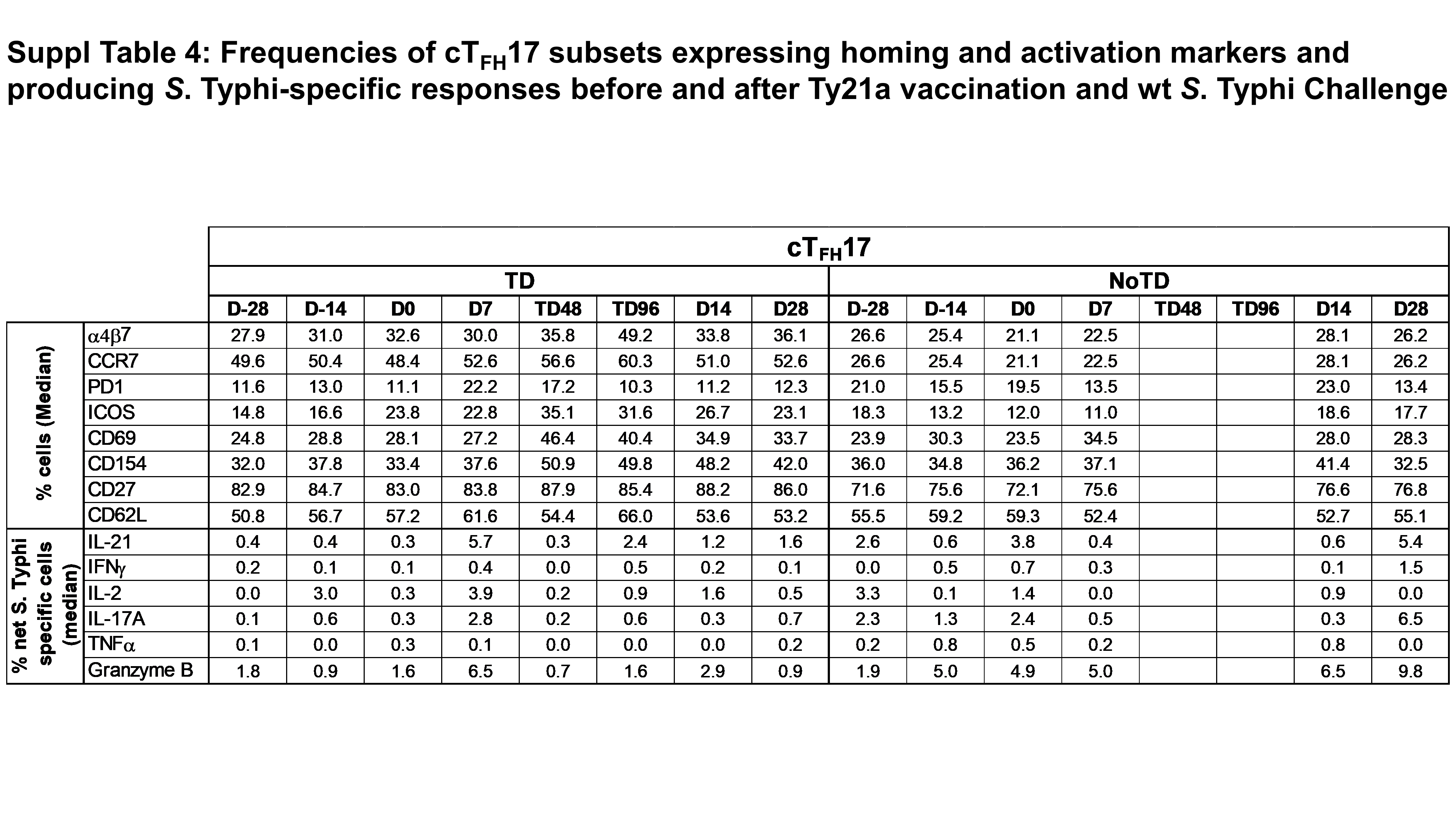

Supplement: Supplementary file 14 [file Image4.tif]

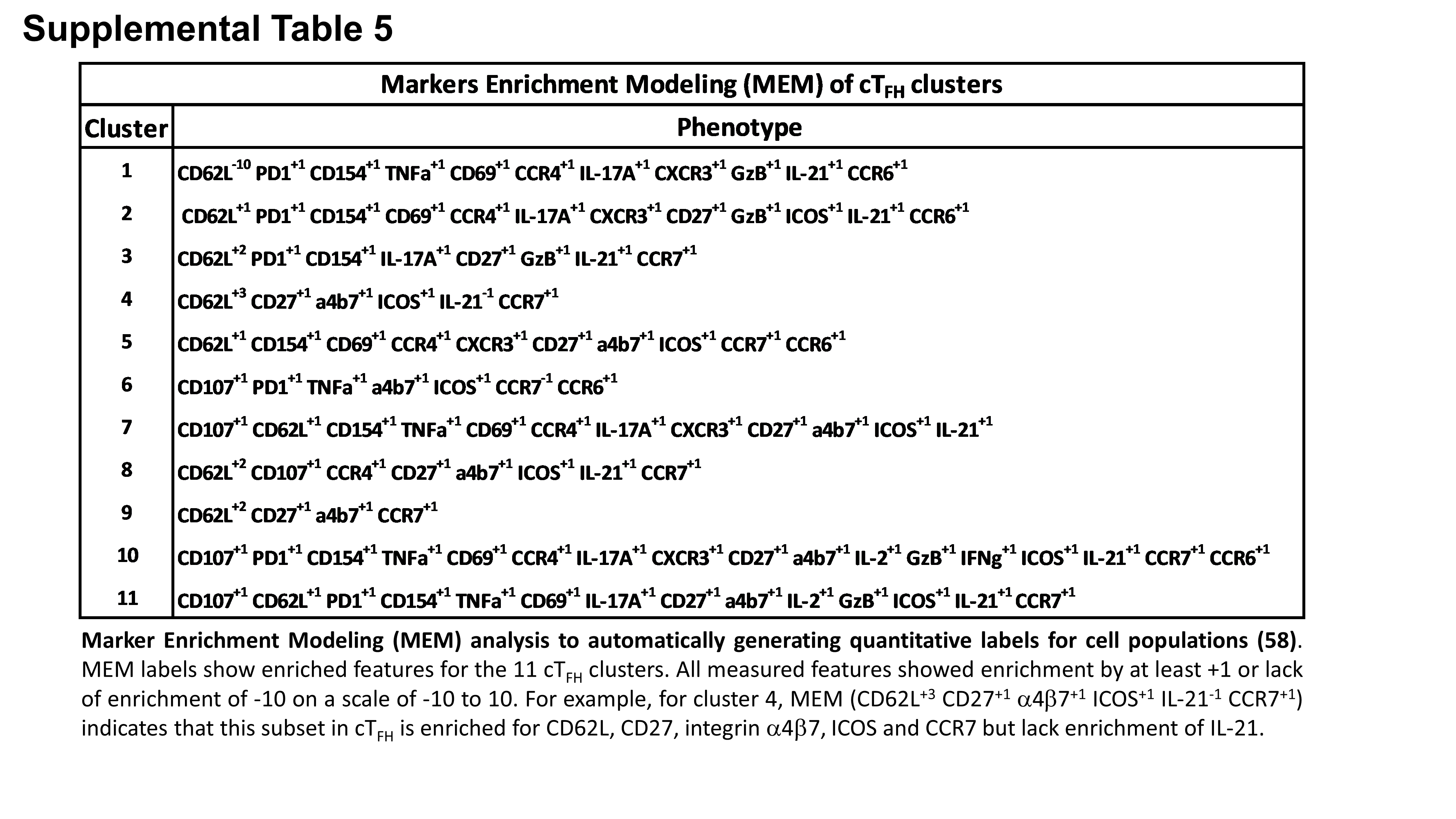

Supplement: Supplementary file 15 [file Image5.tif]
